# Clinical evidence for microbial-derived polyphenol metabolites in health and disease: a scoping review

**DOI:** 10.3389/fnut.2026.1859472

**Published:** 2026-06-17

**Authors:** Jennie Brown, Lydia Norby-Adams, Nancy Ghanem, Ayda Lewis, Joshua Z. Goldenberg, Tiffany Weir, Alexandra Adorno Vita

**Affiliations:** 1 Helfgott Research Institute, National University of Natural Medicine, Portland, OR, United States; 2 Department of Food Science and Human Nutrition, Colorado State University, Fort Collins, CO, United States; 3 Department of Cell and Molecular Biology, Colorado State University, Fort Collins, CO, United States

**Keywords:** cardiometabolic outcomes, dietary (poly)phenols, gut microbiome, metabotypes, microbial-derived (poly)phenol metabolites, precision nutrition, urolithins

## Abstract

**Background:**

Growing translational evidence indicates that the physiologically relevant effects of dietary (poly)phenols are largely mediated by gut microbial metabolism, resulting in bioactive microbial-derived (poly)phenol metabolites (MPMs). However, clinical evidence linking specific MPMs to defined health outcomes has not been comprehensively synthesized.

**Objective:**

This scoping review characterizes clinical studies assessing associations between MPMs and human health and disease.

**Methods:**

We systematically searched MEDLINE (PubMed), Embase (Elsevier), Web of Science (SCIE, ESCI), Scopus, ProQuest Health and Medical, the Cochrane Library, and ClinicalTrials.gov. Eligible studies quantified specific MPMs (e.g., urolithins, phenolic acids) in human biological specimens and assessed associations with biological or clinical outcomes. Two reviewers independently screened and extracted data using standardized forms. Findings were synthesized qualitatively.

**Results:**

Seventy studies across cardiometabolic, inflammatory/oxidative stress, neurological, gastrointestinal, cancer, musculoskeletal, epigenetic, and respiratory domains were included. Evidence was most concentrated in cardiometabolic outcomes (*n* = 38), with recurrent associations involving urolithins and phenolic acid derivatives. This was followed by inflammatory/oxidative stress (*n* = 15) and neurological outcomes (*n* = 11). Musculoskeletal, epigenetic, and respiratory outcomes were least represented. Substantial heterogeneity in study design, metabolite measurement, and outcome reporting limited cross-study comparability.

**Conclusion:**

Specific MPMs may contribute to inter-individual variability in diet-related health responses. Standardized metabolite assessment and prospective trials evaluating direct supplementation of specific microbial metabolites, particularly in individuals with limited systemic exposure, are needed to clarify their role in human health and inform precision nutrition strategies.

## 1 Introduction

(Poly)phenols are plant-derived compounds that are consumed via fruits, vegetables, nuts, beverages (e.g., tea, wine), culinary herbs and spices, and other botanicals in the diet (1). Mounting evidence suggests that these dietary phytochemicals and their microbial-derived polyphenol metabolites (MPMs) modulate clinically relevant biological outcomes in health and disease. While (poly)phenols can directly effect host cells by acting as free-radical scavengers and signaling molecules, altering gene expression and cell activity (2–4), many (poly)phenols exhibit low bioavailability in their consumed form (5). Subsequently, these dietary (poly)phenols accumulate in the colon where they are released from food matrices, deglycosylated, and further (metabolized by the gut microbiota. Accordingly, physiologically relevant bioactivities of these (poly)phenols are largely mediated through interactions with gut microbiota (6–9). Microbial metabolism of (poly)phenols results in the production of bioactive (poly)phenol metabolites that exert physiologic effects both systemically and locally at the intestinal mucosa (4, 9). The microbial metabolism of (poly)phenols and the production of microbial-derived (poly)phenol metabolites (MPMs) not only impact gut microbial community structure (10–12), but also intestinal barrier integrity (13–15), oxidative stress and inflammatory (3, 16–19), neurological (20–22), and cardiometabolic (23, 24) processes.

An emerging hypothesis in the field of (poly)phenol research is that inter-individual variability in response to (poly)phenol-based interventions and habitual dietary (poly)phenol exposure can be linked to differences in the (poly)phenol metabolizing capacity across individual microbiomes (9). Distinct metabolite profiles, also called “metabotypes” or producer phenotypes, have been described for several (poly)phenol classes (8, 9). These differences in metabolite profiles reflect inter-individual differences in the ability to convert parent (poly)phenols into downstream MPMs (8, 9). Examples of metabotypes include, but are not limited to, (1) urolithin metabotypes arising from microbial ellagitannin metabolism, including urolithin metabotype A (urolithin A producers), urolithin metabotype B (urolithin A, iso-urolithin A, and urolithin B producers), and urolithin metabotype 0 (non-producers); (2) equol producers and non-producers arising from microbial isoflavone metabolism; and (3) low-, medium-, and high-producers of other flavonoids, such as microbial flavanone and flavan-3-ol metabolites (8). These metabotypes are associated with marked differences in circulating and urinary metabolite profiles, even under comparable dietary exposure. While certain host factors, such as polymorphisms in intestinal epithelial transporters, can play a role in this variability, it is largely mediated by inter-individual differences in the gut microbiota (8).

As a consequence, individuals consuming similar amounts of (poly)phenol-rich foods may experience substantially different internal exposures to the metabolites largely responsible for downstream biological effects. Increasingly, studies suggest that this variability in microbial metabolite exposure—not dietary intake alone—may contribute to inconsistent or variable findings across (poly)phenol intervention trials (9). In response, the precision nutrition field has begun to shift toward metabolite-centric frameworks, including the direct administration of select MPMs, as a strategy to bypass differences in microbial producer status and thus standardize biologically relevant exposure. For example, randomized controlled trials supplementing urolithin A, a microbial metabolite of ellagitannins that only a subset of individuals’ microbiomes can endogenously produce (25), have demonstrated improvements in mitochondrial gene expression, muscle function, and inflammatory markers independent of baseline producer status (25). These studies illustrate a translational pathway in which direct metabolite supplementation can overcome microbiome-driven variability and provide a more uniform biological stimulus than parent compound intake alone.

This paradigm shift highlights the importance of identifying which MPMs are most consistently associated with clinically relevant outcomes in humans. Such metabolites may represent key mechanistic mediators of (poly)phenol bioactivity and serve as biomarkers of response to dietary intervention or changes in metabolic health, and as candidate targets for future precision nutrition or metabolite-based interventions designed to reduce inter-individual variability.

A preliminary search revealed that although substantial secondary research synthesizes evidence on (poly)phenol-based interventions in human health and disease—including recent comprehensive reviews of (poly)phenol metabotypes (8, 9)—comprehensive syntheses specifically examining the role of individual MPMs in relation to human health outcomes are comparatively limited. Reviews that do include MPMs often present them as a subsection within broader (poly)phenol or metabotype discussions and/or integrate *in vitro* and animal evidence alongside clinical findings, making it difficult to isolate which metabolite–outcome associations have been demonstrated in human populations.

Accordingly, this scoping review evaluates the quantity and characteristics of the existing clinical literature on MPMs in human health and disease. By focusing exclusively on studies reporting associations between specific MPMs and defined health outcomes, this review aims to clarify the current state of evidence and guide the design of future precision nutrition investigations. Additionally, this review intentionally includes not only confirmed MPMs, but also host–microbial co-metabolites and metabolites with potential mixed or uncertain origins. Although some of these metabolites may additionally reflect direct dietary intake, and/or host digestive or metabolic processes, their production, bioavailability, and bioactivity may still be substantially influenced by gut microbial composition and metabolic function. Inclusion of these broader metabolite categories therefore allows for a more comprehensive evaluation of metabolite signatures potentially shaped by host–microbe interactions relevant to human health outcomes. Such knowledge may enable targeted augmentation of key metabolites, through direct supplementation or other strategies-particularly in individuals who are low or non-producers, as a means to standardize biologically relevant exposure and reduce microbiome-driven variability in clinical responses.

## 2 Methods

### 2.1 Search strategy and study selection

**Figure 1:**
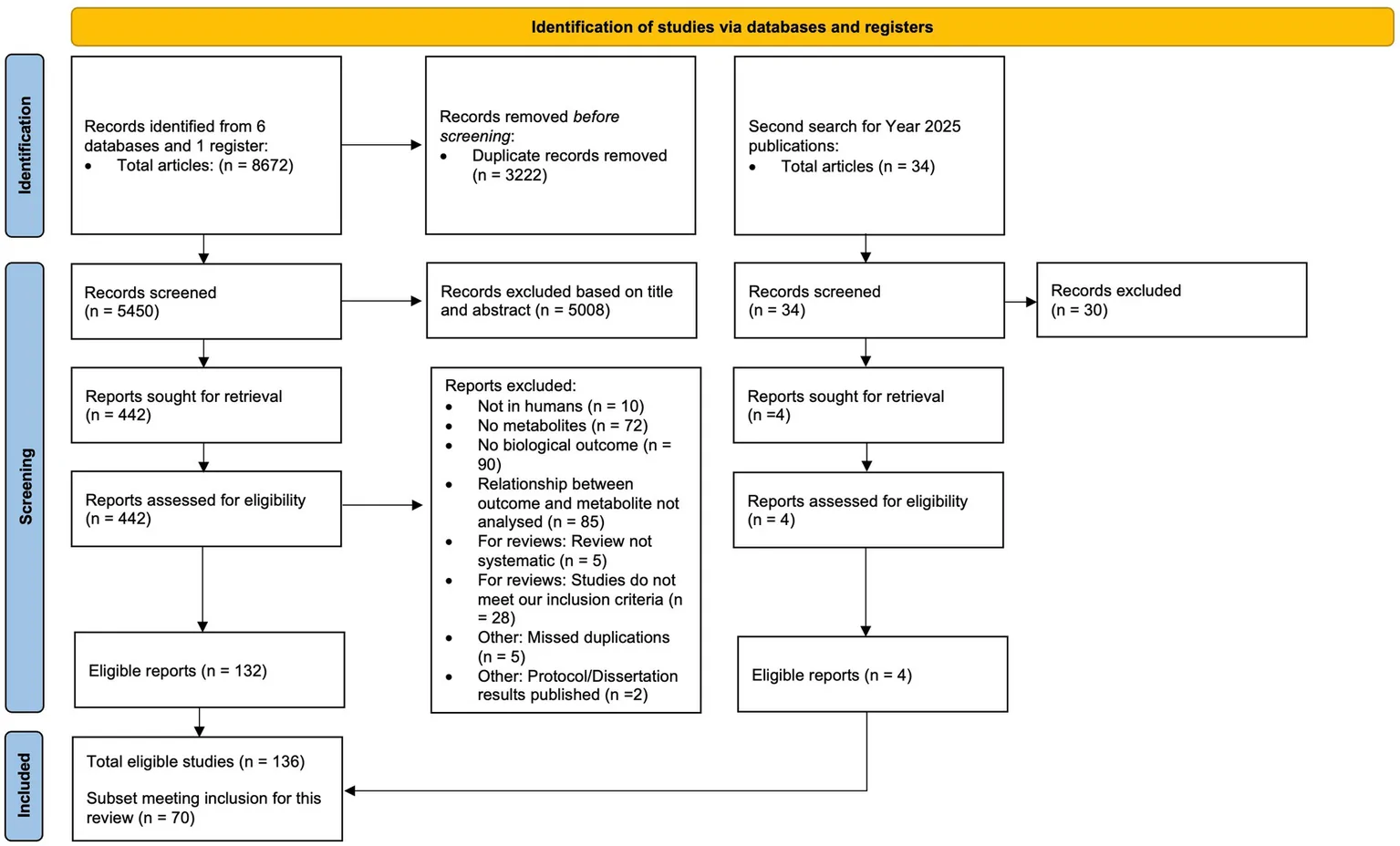
PRISMA flow diagram.

This scoping review followed the JBI methodology for scoping reviews (26) and a predefined protocol registered on Open Science Framework (27). We conducted a comprehensive search—without date, language, or publication status restrictions—of peer-reviewed and gray literature in MEDLINE (PubMed), Embase (Elsevier), Web of Science (SCIE, ESCI), Scopus, ProQuest Health and Medical, the Cochrane Library Database of Systematic Reviews and Central Register of Controlled Trials, and ClinicalTrials.gov. In consultation with content experts, we identified a set of exemplar “seed” articles (*n* = 6) relevant to the research question. Keywords and index terms from the titles and abstracts of these seed articles were used to iteratively develop a PubMed search strategy (Supplementary file 1). The final search string was tested to ensure retrieval of all seed articles prior to use as the final database search (28, 29). This strategy was translated for other databases using the Polyglot Search Translator (30) and refined in consultation with an evidence-synthesis librarian. All retrieved citations were collated in Zotero (Version 7.0.26), de-duplicated, and imported into EppiReviewer (Version 6). Two reviewers independently screened titles and abstracts against inclusion criteria, with full-text review for unclear cases. The same pair then independently assessed full texts; any disagreements were resolved by consensus or a third-party adjudicator. Exclusion reasons were recorded. The study selection process is reported in full and depicted in a PRISMA-ScR flow diagram (Figure 1).

### 2.2 Inclusion and exclusion criteria

As we aimed to summarize and synthesize existing literature regarding the role of MPMs in biological outcomes related to human health and disease, the following *a priori* criteria were applied for inclusion:

- Measurements of MPMs in any biological specimen (e.g., feces, blood, or urine). For this study, MPMs included *de novo* products of microbial (poly)phenol metabolism (e.g., urolithins or phenyl-*γ*-valerolactones), products of microbe-host co-metabolism, and products that have the potential to be microbially derived but may also come from food sources or host catabolic processes (e.g., phenolic acids);
- Measurement of one or more biological outcomes related to health and disease in human participants;
- Statistical analysis of the relationship(s) between MPMs and biological outcome(s).

We considered studies of human subjects regardless of age, sex, ethnicity, geographic location, or health status. Articles were excluded if they were animal studies (*in vivo*), cell culture studies (*in vitro*), or if they were human studies that did not meet the inclusion criteria.

### 2.3 Types of sources

We included all experimental, quasi-experimental, and observational study designs. We also included protocols and trial registries for ongoing studies, published abstracts or conference proceedings, as well as systematic reviews and meta-analyses that synthesized clinical evidence on MPMs in human health and disease. Systematic reviews were defined as those reporting (1) a clearly articulated research question, (2) a reproducible search strategy spanning at least two databases, and (3) a formal appraisal of the evidence quality (31).

### 2.4 Data extraction

Data were independently extracted by two reviewers using piloted Microsoft Excel tables, with any discrepancies resolved by consensus or, when necessary, adjudication by a third reviewer. A content expert and all reviewers agreed on the final extraction items, which included participant characteristics (e.g., age range, sex, race/ethnicity); physiological context (e.g., healthy versus specific disease); geographical location; study design; details of (poly)phenol exposure (e.g., intervention type, dose, duration, controls); metabolites measured; timing and frequency of measurements; biological sample sources (e.g., urine, stool, blood); detection methods (e.g., HPLC, mass spectrometry); biological outcomes; and reported study limitations. The extraction form was iteratively refined during data collection, with modifications implemented by reviewer consensus and expert input. Extracted data were periodically cross-checked (e.g., every 10 articles) to ensure consistency and to identify necessary adjustments to the extraction process.

### 2.5 Data presentation

Due to the substantial volume and heterogeneity of MPMs and associated biological outcomes, the evidence base was organized into three thematic synthesis categories:

- *The current review* focuses on associations between MPMs and host biological outcomes, with emphasis on metabolites derived from anthocyanins, flavanones, flavan-3-ols, flavones, flavonols, phenolic acids, and ellagitannins.
- A complementary synthesis examines associations between these metabolites and gut microbiota composition and function.
- A third synthesis framework addresses phytoestrogen MPMs (e.g., isoflavone- and lignan-derived) in relation to host outcomes.

### 2.6 Defining MPMs

For the purposes of this review, MPMs were broadly defined as: (1) metabolites primarily generated through gut microbial biotransformation of dietary polyphenols (e.g., urolithins, phenyl-g-valerolactones); (2) host–microbial co-metabolites resulting from combined microbial and host metabolic processes, including phase II conjugates of microbial metabolites and compounds such as hippuric acid; and (3) metabolites with potential mixed or uncertain origin, including compounds that may arise from not only microbial metabolism, but also potentially direct dietary intake and/or host digestive and metabolic processes (e.g., several phenolic acids and related metabolites). Given the interconnected nature of dietary, microbial, and host metabolic pathways, metabolites with potential mixed origins were included when discussed in the literature within the context of gut microbial polyphenol metabolism.

Nomenclature for (poly)phenols and their metabolites follows the standardized naming recommendations proposed by Curti et al. (32). For metabolites not included in Curti et al. (32), nomenclature follows the recommendations proposed by Kay et al. (33).

## 3 Results

### 3.1 Overview of included studies

A total of 70 articles were included, representing a wide range of study designs (Table 1). Intervention-based studies include 34 RCTs (14 parallel RCTs (including two acute interventions), six multi-arm RCTs, 11 crossover RCTs (including three acute interventions), three multi-arm crossover RCTs); and eight single-arm studies (including five acute single-arm interventions). Observational studies (21 total) include 10 cross-sectional, six prospective or longitudinal cohort, one retrospective, and four case–control. Other studies include two study protocols, three secondary analyses, and one systematic review and meta-analysis. The identified outcome domains of the included studies span cardiometabolic (38 articles), immunological and oxidative (15 articles), neurological (11 articles), gastrointestinal and digestive health (six articles), cancer (four articles), musculoskeletal (one article), epigenetic (one article), and respiratory (one article). Of the 70 articles, six are represented across more than one outcome domain.

**Table 1:** Characteristics of included studies across outcome domains.

Category	Cardiometabolic	Immunological & Oxidative	Neurological	Gastrointestinal & Digestive	Cancer	Musculoskeletal	Epigenetic	Respiratory
Studies reporting outcome*, N (%)	38 (54%)	15 (21%)	11 (16%)	6 (9%)	4 (6%)	1 (1%)	1 (1%)	1 (1%)
Sample size range (n)	8–2,222	21–6,186	40–6,186	27–54	28–1,053	499	294	30
Study Designs								
Cross-sectional	4	5	1	0	0	1	0	0
Cohort	3	1	2	0	0	0	0	0
Case–control	2	0	0	0	2	0	0	0
RCT	20	6	6	4	1	0	1	1
Acute (<14 days)	4**	0	1**	1	0	0	0	0
Single-arm	6	1	1	0	1	0	0	0
Acute (< 14 days)	4	0	1	0	0	0	0	0
Other	3	2	1	2	0	0	0	0
Locations								
USA	8	3	0	2	1	0	0	0
Europe	28	8	9	3	1	0	0	1
China	0	2	0	0	2	1	0	0
Multi-country	1	2	2	0	0	0	0	0
Israel	1	0	0	0	0	0	1	0
Brazil	0	0	0	1	0	0	0	0

Studies were categorized by outcome domain, study design, and geographic location. Study designs were grouped as randomized controlled trials (RCTs; including parallel, crossover, acute, and multi-arm designs), cross-sectional, cohort (including prospective and longitudinal), case–control (including nested), single-arm, and other (including protocol papers, secondary analyses, retrospective studies, systematic reviews/meta-analyses, and mixed-design analyses). Locations were classified as USA, Europe (including studies form UK, Spain, Italy, France, Germany, Denmark, and Slovakia), China, multi-country studies, Israel, and Brazil. Percentages are based on the total number of included studies (*N* = 70). *Studies reporting multiple outcome domains were counted in each relevant category; therefore, totals exceed 100%; **One study contains both acute and chronic phases.

Given the strong influence of diet and medication use on the gut microbiota, which may subsequently impact MPM production, we assessed the number of studies that implemented criteria to minimize potential dietary and medication-related confounding. Out of the included studies, a total of 30 excluded participants based on antibiotic use; 9 studies had restrictions and/or exclusionary criteria based on fiber, prebiotic, and/ or probiotic use; 14 studies had restrictions and/or exclusionary criteria based on general supplement or nutraceutical use (which may or may not encompass fiber, prebiotics, or probiotics); and 32 studies utilized a dietary run-in or restriction to minimize dietary confounding (Table 2).

**Table 2:** Methodological characteristics and approaches to confounding mitigation across studies included in the review.

Microbiome and MPM relationships assessed (n studies)	Observational studies	Interventional studies^†^
	*n* = 4	*n* = 19
Microbiome outcomes		
Taxa only	*n* = 3 (37, 73, 100)	*n* = 9 (35, 50, 59, 64, 74, 82, 90, 94, 95)
Taxa and diversity	*n* = 1 (43)	*n* = 10 (40, 41, 45, 46, 49, 54, 67, 81, 89, 92)
Microbiome sequencing or detection methods		
16 s rRNA gene methods	*n* = 3 (37, 43, 73)	*n* = 13 (40, 41, 45, 46, 59, 64, 67, 74, 82, 90, 92, 94, 95)
MetaG methods	*n* = 1 (100)	*n* = 3 (50, 54, 89)
qPCR	*n* = 0	*n* = 2 (35, 49)
Dietary confounding mitigation (n studies)		
Dietary run-ins or dietary restrictions	*n* = 3 (63, 76, 85)	*n* = 29 (35, 36, 39, 41, 45, 46, 49, 50, 51, 52, 56, 61, 64, 65, 66, 71, 74, 80, 81, 82, 86, 87, 88, 91, 92, 93, 94, 95, 96, 106)
Fiber, prebiotic, or probiotic exclusion or restriction	*n* = 2 (73, 83)	*n* = 7 (40, 50, 52, 64, 74, 88, 95)
Supplement/nutraceutical exclusion or restriction	*n* = 0	*n* = 14 (41, 42, 46, 49, 54, 56, 59, 65, 77, 80, 81, 89, 96, 106)
Medication confounding mitigation (n studies)		
Antibiotic exclusion or restriction	*n* = 4 (48, 73, 83, 100)	*n* = 26 (35, 36, 38, 40, 41, 46, 49, 50, 52, 54, 56, 59, 64, 65, 67, 70, 74, 81, 88, 89, 90, 92, 94, 95, 96, 106)

Values are presented as number of studies. Total number of studies that statistically assessed relationships between MPMs and microbiota outcomes were further categorized according to whether studies assessed taxonomic composition alone (“taxa only”) or taxonomic composition with diversity metrics (“taxa and diversity”). Microbiome detection methods included 16S rRNA gene-based approaches (“16s rRNA gene”), metagenomic sequencing (“MetaG”), or qPCR-based methods. Dietary confounding mitigation strategies included dietary run-ins, dietary restrictions, and restrictions on polyphenol-rich foods, fiber, prebiotics, probiotics, or supplements prior to and/or during study participation. Medication confounding mitigation strategies included exclusion or restriction of antibiotics. Studies could contribute to multiple categories where applicable. Notations †: one study counted as interventional, Cortés-Martín et al. (59), is a secondary analysis of an interventional study.

Additionally, only a subset of studies statistically assessed relationships between MPMs or metabotypes and gut microbiota (*n* = 23), and among these, the majority relied on 16S rRNA gene-based sequencing methods (Table 2).

Across included studies, well-established microbial-derived metabolites included urolithins, phenyl-*γ*-valerolactones, and dihydroresveratrol generated primarily through gut microbial biotransformation of dietary polyphenols. Host–microbial co-metabolites included hippuric acid, flavanone conjugates, and other phase II conjugated metabolites, which reflect combined microbial and host metabolic processing. Metabolites with potential mixed or uncertain origin included flavanone aglycones, catechol, and several benzoic, phenylacetic, phenylpropanoic, hydroxycinnamic, galloyl/gallotannin-derived, and related aromatic phenolic metabolites that may arise from not only microbial metabolism, but also potentially host digestive and metabolic processes or direct dietary intake. Additionally, certain metabolite classes, such as xanthohumol-derived metabolites, may span multiple categories due to contributions from dietary exposure, microbial biotransformation, and host conjugation pathways.

A detailed account of relationships between MPMs and health outcomes (Supplementary Tables 1A–H), study characteristics and design features (Supplementary Tables 2A–H), and microbiome characterization methods, outcomes assessed, and reported associations between MPMs and microbiome features (Supplementary Tables 3A–E) can be found in the Supplementary materials.

### 3.2 Cardiometabolic outcomes

Thirty-eight studies, including one systematic review and two active trials (indicated via trial registries), assess or plan to assess relationships between MPMs and cardiometabolic outcomes (Tables 3–5). Studies were stratified by population health status as follows: (1) healthy participants, defined as having no diagnosed condition or underlying cardiometabolic dysfunction, or healthy at baseline and followed over time; (2) at-risk participants, defined as having cardiometabolic risk factors without meeting the criteria for a diagnosis (e.g., overweight/obese, dyslipidemia, pre-hypertensive or stage 1 hypertension, etc.), including participants with a spectrum of cardiometabolic risk (e.g., spanning no risk to disease), or participants at-risk at baseline and followed over time; and (3) participants with existing conditions, defined as all or a subset of participants having an official diagnosis of a condition, meeting criteria for a cardiometabolic condition, or developing a cardiometabolic condition over time.

**Table 3:** Summary of studies assessing cardiometabolic outcomes—healthy populations.

Reference	Study design	Population	Sample size (and intervention, if applicable)
Istas et al. (39)	RCT—Crossover, Multi-arm	Healthy young non-obese males	Total (Red raspberry drink of different doses and placebo): 10
Istas et al. (41)	RCT—Three-arm	Healthy males	Total: 86; Aronia Extract: 23; Aronia whole fruit: 23; Control: 20
Li et al. (43)^††^	Cross-sectional	Healthy older female twins	Total: 100 Twin pairs; Monozygotic: 45 pairs; Dizygotic: 55 pairs
Wood et al. (45)	RCT—Parallel	Healthy older adults	Total: 61; WBB Powder: 32; Placebo: 29
Mostafa et al. (44)	Cohort study—Prospective	Subsample from DCH-NG MAX study	Baseline: 624; 6 months: 380; 12 months: 349
Cortés-Martín et al. (38)	Single-arm—Acute	Healthy adolescents and female adults	Total (Mixed nuts): 68; Adolescents: 40; Adults: 28
Cortés-Martín et al. (40)	Single-arm—Acute	Post-partum, breastfeeding mothers	Total (Walnuts): 40
Jamieson et al. (46)	RCT—Parallel	Healthy adults	Total: 27; Xanthohumol: 14; Placebo: 13
Selma et al. (35)^†^	Single-arm—Acute (Two Studies)	Healthy adults	Combined: 69; Study 1 (Walnuts): 20; Study 2 (Pomegranate Extract): 49
Pallister et al. (37)^††^	Retrospective	Healthy adult twins	Total: 2218
Sun et al. (34)	Case–control	Female registered nurses who did not have T2DM at baseline (NHS I and II)	Total: 2222 NHS I—Cases T2D: 456; Controls: 456 NHS II—Cases T2D: 655; Controls: 655
NCT03713164	RCT—Crossover	Healthy adult males with low fiber/(poly)phenol consumption	Total: 19 (Pomegranate juice OR ellagic acid)
Mills et al. (36)	RCT—Multi-arm Crossover (Two Studies)	Healthy adult males	Efficacy Study (low or high (poly)phenol coffee, or control): 15; Proof of Concept: 19 (caffeoylquinic or chlorogenic acid, or control)
Rodriguez et al. (42)	Multiple: Study 1 and 2: RCT—Crossover; Study 3: Single-arm; Study 4: RCT—Parallel	Healthy adult males	Total: 55; Study 1 (Anthocyanin capsules OR WBB powder): 5; Study 2 (Anthocyanin capsules): 10; Study 3 (WBB powder): 5 L; Study 4 (WBB powder): 40

Included studies span randomized controlled trials (parallel, crossover, and multi-arm), observational designs (cross-sectional, cohort, case–control, retrospective), and acute single-arm interventions conducted in generally healthy populations across the lifespan. Sample sizes are reported as total participants and, where applicable, by intervention group. Notations: † and †† indicate studies derived either partially or completely from the same participant cohort across Tables 2–4. RCT, randomized controlled trial; NHS, Nurses’ Health Study; T2DM, type 2 diabetes mellitus; WBB, wild blueberry; DCH-NG MAX, Diet, Cancer and Health—Next Generations MAX study.

**Table 4:** Summary of studies assessing cardiometabolic outcomes—at-risk populations.

Reference	Study design	Population	Sample size (and Intervention, if applicable)
González-Sarrías et al. (18)^†^	RCT—Multi-Arm Crossover	Healthy overweight-obese adults	Total (Pomegranate extract and placebo): 49
Grohmann et al. (56)	RCT—Crossover	Male and post-menopausal female participants at risk of developing T2DM, or those who were diagnosed with pre-diabetes	Total (Bilberry extract and placebo): 14
Cortés-Martín et al. (59)^†^	Secondary analysis of crossover trial	Adults with overweight/obesity	Total (Pomegranate extract and placebo): 49
Lanuza et al. (57)	Cohort Study—Longitudinal	Community-dwelling Danish adults with a range of cardiometabolic health, including healthy, elevated risk, and a subgroup (~10%) with MetS	Baseline: 676; 6 months: 380; 12 months: 348
Le Sayec et al. (54)	RCT—Parallel with acute and chronic phases	Adults with pre-hypertension	Total: 102 Acute Consumption—Aronia berry extract: 51; Placebo: 51 Chronic Consumption—Aronia berry extract: 45; Placebo: 48
Woolf et al. (58)	RCT—Parallel	Estrogen-deficient postmenopausal women, with elevated blood pressure or stage-1 hypertension	Total: 43; WBB powder: 22; Placebo: 21
Zheng et al. (48)	Cohort Study—Prospective	Black adults without hypertension at baseline from the Atherosclerosis Risk in Communities (ARIC); considered in at-risk because follows hypertension risk	Total: 896; Non-incident hypertension: 552; Incident hypertension: 344
Coelho et al. (51)	RCT—Acute Crossover	Adults with overweight/obesity	Total (Grape juice and placebo): 34
Flynn (52)	RCT—Acute Crossover	Older adults with overweight/obesity	Total (WBB powder and placebo): 8 (22 Completed at least 1 arm)
Marhuenda-Muñoz et al. (55)	Case–control	Adults at high CVD risk, but without CVD or diabetes at baseline	Total: 172; Cases: 46; Controls: 126
Zhang et al. (50)	Single-arm—Acute	Non-smoking adults with pre-diabetes and insulin resistance	Total (Red raspberry drink): 36 pre-diabetes and insulin resistance (PreDM-IR): *n* = 26 healthy Reference group: *n* = 10
Zelicha et al. (71)	RCT	Adults with abdominal obesity or dyslipidemia	Total: 286; Healthy Dietary Guidelines (HDG): 98; Mediterranean Diet (MED): 96; Green-MED: 92
NCT06347094	RCT	Adults with cardiometabolic risk factors	Total (High (poly)phenol diet): 330
Huang et al. (65)	RCT—Crossover	Overweight or obese adults with moderate hypercholesterolemia	Total (Strawberry powder and placebo): 34
Laveriano-Santos et al. (53)	Cross-sectional	Adolescents, majority metabolically healthy; crude MetS prevalence 3%	Total: 560

Included studies encompass randomized controlled trials (parallel, crossover, multi-arm, and acute/chronic designs), observational studies (cross-sectional, cohort, case–control), and single-arm acute interventions conducted in populations with elevated cardiometabolic risk (e.g., overweight/obesity, pre-diabetes, hypertension, dyslipidemia, or metabolic syndrome) or at increased risk of disease development. Sample sizes are reported as total participants and, where applicable, by intervention group or timepoint. Notations: † and †† indicate studies derived either partially or completely from the same participant cohort across Tables 2–4. RCT, randomized controlled trial; T2DM, type 2 diabetes mellitus; PreDM-IR, pre-diabetes with insulin resistance; MetS, metabolic syndrome; CVD, cardiovascular disease; ARIC, Atherosclerosis Risk in Communities study; WBB, wild blueberry; HDG, Healthy Dietary Guidelines; MED, Mediterranean Diet.

**Table 5:** Summary of studies assessing cardiometabolic outcomes–existing conditions.

Reference	Study design	Population	Sample size (and intervention, if applicable)
Cortés-Martín et al. (64)	RCT—Crossover	Poly-medicated adults with MetS	Total (Pomegranate extract and placebo): 50
Curtis et al. (66)	RCT—Acute	Overweight or obese middle-aged to older adults with MetS, and without self-reported cognitive dysfunction	Total: 45; freeze-dried BBP: 23; Placebo: 22
Domínguez-López et al. (68)	Cross-sectional	Elderly Mediterranean population with at least 3 cardiovascular risk factors: current smoking, HTN, dyslipidemia, overweight/obesity, family history of premature CVD.	Total: 200
Khan et al. (61)	RCT—Crossover	Adults with diabetes mellitus or at high risk for cardiovascular disease	Total (cocoa powder or control): 42
Rienks et al. (69)	Systematic Review and Meta-Analysis of Observational Studies	Adults with acute coronary syndrome	Total: 1179; Acute Coronary Syndrome: 393; Controls: 786
Mora-Cubillos et al. (62)	RCT—Parallel	Adults with at least three MetS risk factors	Total: 47; Nuts: 24; Control (no nuts): 23
Meroño et al. (67)	RCT—Crossover	Older adults (≥60 yr) living in a residential care setting, with increased intestinal permeability*	Total: 51
Bullón-Vela et al. (63)	Cross-sectional	Individuals with overweight/obesity and at least three MetS components	Total: 266
Cortés-Martín et al. (70)^†^	Single-Arm (Multiple studies—including current and secondary analysis)	Current study included healthy children, adolescents, and adults; Past studies included adults with MetS, prostate cancer, and colorectal cancer	Current study (walnuts, or pomegranate juice or extract)—Healthy Children (5-12y): 202; Healthy Adolescents (13-18y): 221; Healthy Adults (19-72y): 221 Secondary analysis (Pomegranate extract or walnuts)—Healthy Adults: 109; Metabolic Syndrome Adults: 23; Prostate Cancer Adults: 28; CRC Adults: 35

Included studies comprise randomized controlled trials (parallel, crossover, and acute designs), observational studies (cross-sectional), single-arm interventions, and one systematic review and meta-analysis, conducted in populations with clinically defined conditions (cardiometabolic or other). Some studies also include mixed populations with both healthy individuals and those with diagnosed conditions. Sample sizes are reported as total participants and, where applicable, by intervention group or subgroup. Notations: † and †† indicate studies derived either partially or completely from the same participant cohort across Tables 2–4. RCT, randomized controlled trial; MetS, metabolic syndrome; HTN, hypertension; CVD, cardiovascular disease; BBP, blueberry powder; CRC, colorectal cancer.

Thirteen studies, including interventional and observational studies (*n* = 10 to *n* = 2,222), assessed outcomes in healthy adults (34–46); one trial registry (NCT03713164) indicates plans to measure such associations (47). Thirteen studies, including interventional and observational studies (*n* = 8 to *n* = 896) (48–59), assessed outcomes in participants at elevated risk for cardiometabolic disease (e.g., pre-diabetic, pre-hypertensive, overweight/obese); one trial registry (NCT06347094) indicated plans to measure such associations (60). Ten studies, including interventional and observational studies (*n* = 34 to *n* = 839) (38, 61–68) and one systematic review and meta-analysis (69), assessed outcomes in populations with existing health conditions (e.g., metabolic syndrome, 3 + cardiovascular disease risk factors, diabetes, hypercholesterolemia, acute coronary syndrome, and “leaky gut” [intestinal hyperpermeability]).

Several studies in this section utilized complete or partial datasets derived from the same cohorts and are annotated as such in Tables 3–5. Despite overlap in participant populations, these publications were retained as separate studies because they reported distinct outcomes, evaluated different exposures or interventions, or used the data to address different research questions through comparisons across populations or intervention groups.

#### 3.2.1 Anthropometric measures

Of the studies examining relationships between MPMs and anthropometric measures, five were in healthy participants (35, 37, 40, 46, 70), three were in at-risk populations (53, 57, 71), and three were in participants with existing conditions (38, 62, 67).

Across healthy, at-risk, and clinical populations, urolithin metabotypes have been linked to differences in body composition and adiposity. Three studies reported that individuals classified as urolithin metabotype B displayed higher BMI and a greater prevalence of overweight and obesity (35, 38, 40), whereas urolithin metabotype A was more common among normoweight individuals (35, 40). Cortéz-Martín et al. (38) observed that urolithin metabotype distribution was correlated with BMI only between ages 5–40, with urolithin metabotype B corresponding to an increased shift in BMI compared to metabotype A (38). In a subsequent study of postpartum women, the same researchers correlated urolithin metabotype A with greater weight loss, reduced BMI, and smaller waist circumference compared to metabotype B (40).

At the individual metabolite level, increased concentrations of urolithin A conjugates inversely correlated with waist circumference, waist-to-hip ratio, and/or visceral adipose tissue in cardiometabolic at-risk and clinical (e.g., MetS) subjects (57, 62, 71). Additionally, in adolescents with a range of cardiometabolic health statuses (including healthy, MetS risk factors, and diagnosed MetS), there was an inverse relationship between urolithin B and its liver conjugates with abdominal obesity and waist circumference z-scores (53).

Beyond urolithins, hippurate (hippuric acid), 4-methylcatechol sulfate, and 3,4,5-trihydroxybenzoic acid [gallic acid] derivatives were all inversely correlated with visceral or abdominal fat volume (37, 44, 53). In contrast, 8-prenylnaringenin was elevated in overweight individuals (46). Finally, another study found no associations between proanthocyanidin metabolites (including phenyl-*γ*-valerolactones and phenylvaleric acids) and BMI (70).

#### 3.2.2 Blood pressure

Of the studies examining relationships between MPMs and blood pressure outcomes, one study was in healthy adults (45), six were in at-risk populations (48, 52–54, 56, 57), and one was in participants with 3 + CVD risk factors (grouped under existing conditions) (68). In the study assessing blood pressure in healthy adults, systolic blood pressure was consistently inversely associated with benzoic acid, phenylpropanoic acid, cinnamic acid, and hippuric acid derivatives (45).

Assessment of blood pressure associations with MPMs were more abundant in at-risk populations and demonstrates heterogeneity in relationships observed across related metabolite classes. For example, in one study, 24-h systolic and diastolic blood pressure were uniformly and positively associated with a broad range of metabolites, including phenyl-γ-valerolactone derivatives, benzoic acid derivatives (including dihydroxy-, methoxy-, and sulfated forms), phenylpropanoic acids, and hydroxyhippurate-related metabolites (54). In contrast, other at-risk cohorts spanning adult and adolescent populations demonstrated predominantly inverse associations between clinic-based SBP, DBP, or overall BP and several dihydroxybenzoic acid isomers, phenylacetic and phenylpropanoic acid derivatives, hydroxycinnamic acids, cinnamoyl-glycine, urolithins (A, B, and glucuronide), and dihydroresveratrol (53, 56, 57). Low anthocyanin metabolizer status, defined as individuals in the lowest tertile of circulating anthocyanin-derived metabolite concentrations following blueberry consumption, was also associated with higher blood pressure (52). Additionally, while hippuric acid derivatives were inversely associated with blood pressure in the study of healthy adults (45), 4-hydroxyhippurate and related metabolites were positively associated with blood pressure (54) or higher risk of hypertension in at-risk populations (48). Finally, in elderly adults with 3 + CVD risk factors, urolithin B-glucuronide was positively associated with diastolic blood pressure (68).

#### 3.2.3 Blood lipids

Out of the studies examining relationships between MPMs and blood lipids, none were in healthy populations, five were in at-risk populations (49, 52, 53, 57, 59), and five were in participants with existing conditions (e.g., MetS, T2D, 3 + CVD risk factors) (61–63, 66).

Among at-risk populations, urolithin associations with blood lipids differed by both metabotype classification and specific urolithin (species/form). González-Sarrías et al. (49) observed that although urolithin metabotype B individuals exhibited elevated cardiovascular risk factors (e.g., oxidized LDL-c, total cholesterol, LDL-c, small LDL-c, non-HDL-c, and ApoB) at baseline when compared to urolithin metabotype A, metabotype B individuals saw greater reductions in these risk factors post-intervention with pomegranate extract when compared with metabotype A (49). Consistent with this pattern, only in metabotype B individuals were concentrations of individual urolithin metabolites (e.g., urolithins A, iso-A, B, and total) also inversely correlated with cardiovascular risk factors (49). Additionally, plasma urolithin A conjugates were positively correlated with HDL-c (57) in an observational cohort of adults with a range of cardiometabolic health status (ranging healthy, at-risk, and MetS), whereas in overweight-obese adults, fecal urolithin A was inversely associated with small HDL-c and ApoA-1 (59). Beyond urolithins, 3,4,5-trihydroxybenzoic acids, dihydroxybenzoic acids, and cinnamoyl-glycine displayed the most consistent inverse associations with adverse lipid parameters in adolescent (53) and adult (57) cohorts spanning a range of cardiometabolic health status (healthy, at risk, and MetS), although associations with other select phenolic acids were more variable.

In populations with existing conditions, several MPMs were associated with favorable cardiometabolic profiles. Triglycerides were inversely associated with trans-3,5,4′-trihydroxystilbene [resveratrol]-derived metabolites (63). Higher HDL-related measures—including HDL-c and XL-HDL particle concentrations—were positively associated with phenylacetic acid derivatives, benzoyl conjugates, 4-Hydroxy-3-methoxybenzoic acid [vanillic acid] derivatives, and hippuric acid (and its sulfate) (61, 66). LDL-related markers demonstrated predominantly inverse associations, with urolithin B-glucuronide inversely associated with LDL-c, and phenylacetic and 4-hydroxy-3-methoxybenzoic acid derivatives inversely associated with oxidized LDL-c (61, 68). ApoA was positively associated with a sulfated methoxyphenylacetic acid derivative, while total cholesterol was inversely associated with N-benzoyl-L-glutamic acid and benzoic acid-4-sulfate (66). However, despite numerous significant associations in populations with existing conditions, five studies also reported null findings between select metabolites or aggregate MPM scores and lipid endpoints (62–64, 67, 68).

#### 3.2.4 Glycemic outcomes

Of the studies examining relationships between MPMs and glycemic outcomes, none were in healthy adults; five were in at-risk populations (49–52, 57), and two were in participants with existing conditions (e.g., MetS and Leaky Gut) (62, 67).

For urolithins, associations appeared to differ by metabotype (e.g., UM-A, UM-B, UM-0) versus individual metabolite concentration and by population. In subjects with metabolic syndrome, insulin and HOMA-IR were inversely associated with urolithin A glucuronide (62). Consistent with this pattern, individuals with pre-diabetes or insulin resistance exhibited lower concentrations of multiple urolithin A derivatives compared with healthy control populations (50). However, insulin and HOMA-IR did not significantly differ by urolithin metabotype in at-risk adult populations (49) or in adults with increased intestinal permeability (67), and across three studies that included at-risk or MetS populations, neither urolithin metabotypes nor urolithin A concentrations were associated with fasting blood glucose (49, 62, 67).

Inverse associations with glycemic markers were observed for several non-urolithin phenolic acid derivatives in at-risk populations, including 4′-Hydroxy-3′-methoxycinnamic acid and 3′-hydroxy-4′-methoxycinnamic acid glucuronides with blood glucose (51) and 2,6-dihydroxybenzoic acid and cinnamoyl-glycine with HbA1c (57). However, the sum of anthocyanin metabolites, including MPMs, was not associated with glucose in at-risk adults (52).

#### 3.2.5 Vascular function

Of the studies examining relationships between MPMs and vascular function, four were in healthy adults (36, 41, 42, 45), four were in at-risk populations (53, 55, 59, 66), and none were in participants with existing conditions.

The only vascular function outcome assessed in healthy populations was flow-mediated dilation, where the vast majority of assessed MPMs (54/59 metabolites), including urolithin-A conjugates, flavan-3-ol catabolites, phenolic acids (hydroxybenzoic, hydroxycinnamic), and select hippuric and phenylacetic acid derivatives were positively associated with flow-mediated dilation, suggesting greater nitric oxide–dependent vasodilation (36, 39, 41, 42, 45); only a small number (4/59) of specific hydroxyhippuric, phenylacetic, and hydroxybenzoic acid species were inversely associated with FMD.

In at-risk adults, individual MPMs demonstrated mixed associations: only three MPMs (specific 3-(4-hydroxyphenyl)propanoic acid-3-sulfate, hydroxybenzoic acid conjugates, and phenylacetic acids) were positively associated with FMD (58, 65), whereas several dihydroxybenzoic, phenylacetic, and hippuric acid derivatives were inversely associated, including metabolites observed during placebo conditions (58, 65). Resting blood vessel diameter was also inversely associated with a hippuric acid derivative (65).

Regarding markers of arterial elasticity, MPMs were uniformly associated with lower pulse wave velocity following an aronia berry intervention (54). In contrast, relationships with augmentation index (awake and 24-h AIXao and AIXbr) were mixed. While two specific benzoic acid derivatives, 2,4- and 2,6-dihydroxybenzoic acid, were consistently positively associated with AIX (indicating less favorable vascular elasticity), the majority of other MPMs, including phenyl-*γ*-valerolactone, phenylpropanoic, hydroxycinnamic, and (3/4 & 4/3) conjugated hydroxybenzoic acid derivatives, were inversely associated with AIX outcomes (54). Additionally, adults classified as low anthocyanin metabolizers also experienced increased AIX (less favorable vascular elasticity) compared to their high metabolizer counterparts (52).

Additionally, Rodriguez-Mateos et al. (70) found associations between cinnamic acids, benzoic acids, benzaldehydes, propanoic acids, and quercetin, and 15 genes that are involved in inflammation and/or are functionally linked to cardiovascular disease development. These metabolites accounted for 73–99% of the variability in gene expression (42).

#### 3.2.6 Disease risk, aggregate scores, and other composite measures

Of the studies examining relationships between MPMs and cardiometabolic risk scores and disease outcomes, two were in healthy adults (34, 43), two were in at-risk populations (53, 55), and one was in participants with 3 + CVD risk factors (grouped under existing conditions) (68) or a systematic review that reported outcomes in participants with existing conditions (69).

The aforementioned findings regarding relationships between MPMs and endothelial or cardiometabolic parameters extend to broader risk and composite measures, such as disease-specific risk and composite cardiovascular health scores. In healthy adults, several urinary MPMs—including benzoic, phenylpropanoic, cinnamic, and phenylethanol derivatives—were inversely associated with estimated atherosclerotic cardiovascular disease (ASCVD) risk and the likelihood of major adverse cardiac events (via HEART Score), particularly among individuals with lower habitual (poly)phenol intake (43).

Evidence in populations at elevated cardiometabolic risk (e.g., overweight, pre-diabetes, or multiple CVD risk factors) or existing conditions suggests broadly similar associations. Higher MPM scores, driven in part by urolithin B glucuronide and select benzoic and propanoic acid derivatives, were associated with more favorable composite cardiovascular health indices, reflecting healthier diet quality and glycemic control, although associations with individual components such as blood pressure and lipid markers were inconsistent (68). Likewise, in adolescents, higher circulating levels of urolithin B and 3,4,5-trihydroxybenzoic acid were inversely associated with metabolic syndrome scores (53).

In prospective analyses, higher excretion of specific MPMs was associated with reduced future disease risk. Based on tertiles of urinary metabolite concentrations, higher excretion of hydroxybenzoic acid glucuronide, as well as intermediate concentrations of 4-hydroxybenzoic acid, 2-(3,4-dihydroxyphenyl)ethanol sulfate (sulfation position not determined) (hydroxytyrosol sulfate), 3-methoxybenzoic acid-4-sulfate (vanillic acid sulfate), and 3′-Hydroxycinnamic acid [m-coumaric acid], were associated with lower odds of developing type 2 diabetes in at-risk adults (55). Similarly, in adults without diagnosed cardiometabolic disease, higher urinary excretion of the flavanone metabolite hesperetin was associated with a reduced risk of incident type 2 diabetes in a prospective case–control study (34).

Despite some data linking MPMs to composite cardiometabolic risk measures, a systematic review reported no consistent associations between (poly)phenol metabolites and acute coronary syndrome outcomes, including myocardial infarction and unstable angina (69).

### 3.3 Inflammatory and oxidative stress outcomes

A total of 15 studies assessed inflammatory and oxidative stress markers in relation to MPMs, and two protocols plan to assess these outcomes (Table 6). Sample sizes ranged from 21 to > 6,000, and populations spanned healthy adults to adults with obesity, metabolic syndrome, chronic obstructive pulmonary disease (COPD), pemphigus, Crohn’s disease, and increased intestinal permeability. Interventions included pomegranate extract, pomegranate juice, mango pulp, a (poly)phenol-rich diet, whole grain biscuits, or xanthohumol.

**Table 6:** Summary of studies assessing immunological or oxidative outcomes.

Reference	Study design	Population	Sample size
Cortés-Martín et al. (64)	RCT—Crossover	Adults with metabolic Syndrome (MetS) under polypharmacy treatment	Total (Pomegranate extract or placebo): 50
Barnes et al. (82)	RCT—Three arm	Healthy lean and obese adults without digestive disorders	Total: 32; Lean (BMI < 25), Control: 11; Lean, Mango: 12; Obese (BMI > 30), Mango: 9
Angelino et al. (78)	Protocol for Cohort	Community-dwelling older adults without diagnosed dementia	Total: 6186; Original TUDA: 5186; TUDA5+: 1000
Cerdá et al. (80)	RCT—Parallel	Adults with stable COPD	Total: 30; Pomegranate juice: 15; Placebo: 15
González-Sarrías et al. (81)	RCT—Crossover	Overweight-obese adults	Total (Pomegranate extract or placebo): 49
Gutiérrez-Díaz et al. (73)	Cross-sectional	Healthy, older adults	Total: 71
Guo et al. (83)	Cross-sectional	Adults with pemphigus	Total: 69; Pemphigus: 43; Controls: 26
Langley et al. (77)	Protocol for RCT	Adults with clinically active Crohn’s disease	Total: 32; Xanthohumol: 16; Control: 16
Kim et al. (72)	Single-arm	Lean and obese human adults	Total: 21; Lean (BMI < 25), Mango: 12; Obese (BMI > 30), Mango: 9
Vitaglione et al. (74)	RCT—Parallel	Healthy overweight or obese adults with low intake of fruit and vegetables and sedentary lifestyle	Total: 68; Whole grain: 36; Control snacks: 32
Meroño et al. (67)	RCT—Crossover	Older adults (≥60 yr) living in a residential care setting, with increased intestinal permeability	Total ((poly)phenol-rich snacks or control snacks): 51
Harms et al. (75)	Cross-sectional	Adults ≥35 years old from European countries, free of major chronic diseases	Total: 315
Dorjgochoo et al. (79)	Cross-Sectional	Healthy middle-aged and elderly Chinese women	Total: 845
Bullón-Vela et al. (76)	Cross-sectional	Overweight/obese adults diagnosed with MetS	Total: 267
Lanuza et al. (57)	Longitudinal Cohort	Adults from Copenhagen and their biological children, spouses, and grandchildren	Baseline: 676; 6 months: 380; 2 months: 348

Included studies encompass randomized controlled trials (parallel, crossover, and multi-arm designs), observational studies (cross-sectional and longitudinal cohorts), single-arm interventions, and study protocols conducted across a range of populations. Sample sizes are reported as total participants and, where applicable, by intervention group or subgroup. Abbreviations: RCT, randomized controlled trial; MetS, metabolic syndrome; COPD, chronic obstructive pulmonary disease; BMI, body mass index; TUDA, Trinity Ulster Department of Agriculture cohort.

#### 3.3.1 Cytokines and systemic inflammatory markers

Three studies evaluated relationships between MPMs and cytokines (67, 72, 73). Pro-inflammatory cytokines IL-8 and IL-17 were positively associated with phenylacetic acid, with IL-8 additionally associated with phthalic acid and total phenolic content (73). TGF-*β*, a pleiotropic cytokine with context-dependent inflammatory roles, was positively correlated with phenylacetic acid, phthalic acid, 3,4-dihydroxybenzoic acid [protocatechuic acid], and total phenolic content (73). In contrast, the anti-inflammatory cytokine IL-10 was positively associated with galloyl metabolites (72) and phenylacetic acid (73).

Six studies evaluated relationships between MPMs and acute phase or systemic inflammatory markers (57, 67, 73–76). C-reactive protein (CRP) was positively associated with phenylacetic acid in a cross-sectional analysis (73). In contrast, high-sensitivity CRP (hsCRP) was inversely associated with several phenolic acid derivatives, including 2,6- and 3,4-dihydroxybenzoic acid, 3,4- and 3,5-dihydroxyphenylpropionic acid, caffeic acid, cinnamoyl-glycine, 4′-hydroxy-3′-methoxycinnamic acid, and 2-(3′-4′-Dihydroxyphenyl)ethanol [hydroxytyrosol] (57, 75). Indices of systemic inflammation also showed inverse associations with flavanone metabolites: naringenin-7-O-β-D-glucuronide was inversely associated with both the aggregate index of systemic inflammation (AISI) and the systemic inflammation index (SII) (76). Additionally, plasminogen activator inhibitor-1 (PAI-1), a fibrinolytic regulator linked to cardiometabolic inflammation, was inversely associated with 3-(4′-hydroxy-3′-methoxyphenyl)propanoic acid and 4′-hydroxy-3′-methoxycinnamic acid following a whole grain dietary intervention (74).

However, not all metabolite classes demonstrated significant relationships; Meroño et al. (67) reported no association between urolithin metabotypes or circulating urolithins and CRP or measured cytokines in older adults with increased intestinal permeability.

Finally, two published protocols outlined plans to assess relationships between MPMs and inflammatory markers. The protocol for Langley et al. (77) described their planned analyses evaluating the effects of xanthohumol on cytokine concentrations (e.g., IL-1, IL-10, IL-12, IL-17, TNF-*α*) and platelets in adults with Crohn’s disease (77), while the protocol for Angelino et al. (78) outlined their planned analysis of phenyl-*γ*-valerolactones in association with concentrations of the inflammatory marker CRP and cytokines (e.g., IL-6, IL-10, TNF-α) (78).

#### 3.3.2 Oxidative stress markers

Four studies evaluated relationships between MPMs and oxidative stress markers (73, 76, 79, 80). Markers related to oxidative liver and systemic stress demonstrated metabolite-specific associations. γ-Glutamyl transferase (GGT) was inversely associated with hesperetin-3′-O-β-D-glucuronide, naringenin-4′-O-β-D-glucuronide, and naringenin-7-O-β-D-glucuronide (76), whereas phenylacetic acid was positively correlated with malondialdehyde (MDA), a marker of lipid peroxidation (73). However, in COPD patients, urolithin A and B were not associated with Trolox Equivalent Antioxidant Capacity (TEAC) or urinary 8-iso-prostaglandin F_2α_ following pomegranate juice intervention (80). Likewise, Dorjgochoo et al. (79) reported no significant associations between microbial-derived phenyl-γ-valerolactones and oxidative stress biomarkers; however, urinary quercetin (a parent (poly)phenol) was inversely associated with oxidative stress markers (79).

#### 3.3.3 Lipopolysaccharide and lipopolysaccharide-binding protein

Four studies investigated the relationship between MPMs and markers of endotoxemia, including lipopolysaccharide, lipopolysaccharide-binding protein, or both (64, 77, 81, 82). Pomegranate extract reduced lipopolysaccharide-binding protein only at the higher dose of 1800 mg/day, but the changes were not linked to urolithin metabotypes (81). Similarly, Cortés-Martín et al. (64) also reported no lipopolysaccharide-binding protein differences by urolithin metabotype (64). Following a mango intervention, decreased endotoxin levels correlated inversely with gallotannin-derived metabolites (82). Finally, Langley et al. (77), presented their study protocol to examine lipopolysaccharide and lipopolysaccharide-binding protein responses to xanthohumol supplementation (77).

#### 3.3.4 Cell adhesion molecules

Two studies evaluated relationships between MPMs and cell adhesion molecules, such as ICAM, VCAM, and/or MCP-1, in relation to urolithin metabotypes and/or urolithins. Both studies observed no associations with urolithin metabotype (64, 67), with one also noting no associations with individual urolithin metabolite concentrations (67).

#### 3.3.5 Disease-specific contexts

In a case–control study of pemphigus patients, decreased vanillin acetate, 2-hydroxy-3-(4-hydroxyphenyl) propanoic acid, cinnamic acid, benzyl sulfate, and several parent (poly)phenols were observed in pemphigus patients compared with healthy controls (83). These metabolites were also inversely correlated with bacterial taxa enriched in pemphigus (83).

### 3.4 Neurological outcomes

A total of 11 studies examined the relationship between MPMs and neurological outcomes (Table 7). These studies included randomized controlled trials (RCTs), cohort studies, cross-sectional analyses, and acute interventions (*n* = 40–6,000). Most studies enrolled older adults, often with mild cognitive impairment, metabolic syndrome, or overweight/obesity. MPMs investigated include flavan-3-ol metabolites (e.g., phenyl-*γ*-valerolactones), urolithins, and benzoic, cinnamic, phenylacetic, and hippuric acid derivatives. Studies assessed a range of neurological outcomes, including cognitive domains of episodic memory, executive function, attention, processing speed, mood, and global cognition, as well as brain oxidative metabolism and postoperative pain.

**Table 7:** Summary of studies assessing neurological outcomes.

Reference	Study design	Population	Sample size (and intervention, if applicable)
Angelino et al. (78)	Protocol for Cohort Study	Community-dwelling older adults without diagnosed dementia	Total: 6186; Original TUDA: 5186; TUDA5+: 1000
Bensalem et al. (86)	RCT—Parallel	Healthy older adults with mild memory decline	Total: 215; Combined grape and WBB extracts: 101; Placebo Group: 105
González-Domínguez et al. (84)	Cohort Study—Prospective	Cognitive decline in older adults	Bordeaux sample set (training)—Cases: 209; Controls: 209 Dijon sample set (validation)—Cases: 212; Controls: 212
Curtis et al. (87)	RCT—Multi-arm with acute and chronic phases	Overweight or obese middle-aged to older adults with metabolic syndrome, but without self-reported cognitive dysfunction	Chronic Study—Total: 115; BBP Dose 1 (26 g): 37; BBP Dose 2 (13 g): 39; Placebo: 39 Acute (Postprandial) Study—Total: 33; BBP Dose 1 (26 g): 16; Placebo: 17
Domínguez-López et al. (84)	Cross-sectional	Overweight or obese middle-aged to older adults with metabolic syndrome	Total: 400
Flanagan (89)	RCT—Parallel	Middle-aged to older adults with no subjective or objective memory complaints (cognitive impairment)	Total: 60; Cranberry: 29; Placebo: 31
Parilli-Moser et al. (88)	RCT—Three-arm	Healthy adults	Total: 63; Skin-roasted peanuts: 21; Peanut butter: 23; Control butter: 19
Rabassa et al. (85)	Cohort Study—Prospective	Dementia-free older adults	Total: 119; Non-consumers: 72; Regular consumers: 47
Romo-Vaquero et al. (90)	Single-Arm—Acute (Condition Comparison)	Patients with Parkinson’s disease	Total (Walnuts): 169; Parkinson’s disease: 52; Healthy controls: 117
Volpp et al. (91)	RCT—Parallel	Female adults with a medical indication for vaginal hysterectomy	Total: 66; Oak wood extract (Robuvit®): 33; Placebo: 33
Wood et al. (45)	RCT—Parallel	Healthy older adults	Total: 61; BBP: 32; Placebo: 29

Included studies comprise randomized controlled trials (parallel, crossover, and multi-arm designs with acute and/or chronic phases), observational studies (prospective cohorts and cross-sectional analyses), single-arm acute interventions, and study protocols conducted in those with neurological or related conditions (e.g., cognitive decline, Parkinson’s disease, and metabolic syndrome). Sample sizes are reported as total participants and, where applicable, by intervention group, study phase, or subgroup. RCT, randomized controlled trial; WBB, wild blueberry; BBP, blueberry powder; TUDA, Trinity Ulster Department of Agriculture cohort.

#### 3.4.1 Cognitive function

Three observational analyses reported associations between MPMs and cognition-related outcomes. In a cross-sectional study of adults with metabolic syndrome (n = 400), 3,4-dihydroxybenzoic acid, 3-methoxybenzoic acid-4-glucuronide [vanillic acid glucuronide], and 3-hydroxybenzoic acid were positively associated with global cognition scores (a multi-instrument composite score), with 3,4-dihydroxybenzoic acid also being associated with cognitive function/absence of cognitive decline (as measured via Mini-Mental State Examination; MMSE) scores (84). Similarly, in a three-year prospective cohort study of older adults, urinary urolithins A and B, urolithin conjugates, and 2-hydroxyphenylacetic acid were positively associated with better change in cognitive function and inversely associated with odds of cognitive decline, as measured by MMSE (85). Several multi-metabolite panels were also associated with improvements in cognitive function and reduced odds of cognitive decline (84). However, not all studies reported significant associations. In a randomized trial of older adults receiving (poly)phenol-rich grape and blueberry extracts for 6 months, microbial-derived flavan-3-ol metabolites, such as phenyl-γ-valerolactones, showed no relationship with changes in cognitive function (86).

#### 3.4.2 Episodic (visual and verbal) and working memory

Relationships between MPMs and memory-related domains, including episodic and working memory-related outcomes, were evaluated across four studies (45, 87–89). In an RCT of cranberry supplementation, hippuric acid was positively associated with improved episodic memory (89). A separate RCT evaluating peanut-based interventions identified strong correlations between several hydroxybenzoic acid derivatives (e.g., 3-hydroxybenzoic acid, 3,5-dimethoxy-4-(sulfooxy)benzoic acid [syringic acid sulfate], (3/4)-dihydroxybenzoic acid sulfate [protocatechuic acid sulfate], and others) and improvements in verbal, working, and total memory scores (88). Similarly, a multi-arm RCT of blueberry supplementation found that cinnamic, benzoic, phenylacetic, and hippuric acid derivatives were associated with enhanced episodic (total and visual recognition) and working memory (including immediate memory) in both acute and chronic intervention phases (87). Extending these findings, Wood et al. (45) reported that, following 12 weeks of freeze-dried wild blueberry supplementation, immediate verbal recall in healthy older adults was positively correlated with multiple benzoic, phenylacetic, and phenylpropanoic acid derivatives, while delayed verbal recall was associated with glycine-conjugated and methoxylated phenylpropanoid metabolites (45).

#### 3.4.3 Executive function and attention

Three studies assessed domains of executive function and/or attention in relation to MPMs (45, 87, 88). Curtis et al. (87) reported that both postprandial and chronic improvements in attention and executive function following blueberry supplementation were positively associated with several phenylacetic, benzoic, and hydroxycinnamic acid derivatives; alertness was additionally associated with 4-hydroxy-3,5-dimethoxyphenylacetic acid [homosyringic acid] (87). Additionally, Parilli-Moser et al. (88) observed that higher concentrations of hydroxybenzoic acids and their sulfated conjugates were inversely associated with cognitive flexibility scores post-peanut intervention, indicating better task-switching performance (88).

However, findings were not uniformly beneficial; in a randomized trial of freeze-dried wild blueberries, pre–post increases in dihydroxybenzoic acids were inversely associated with changes in task-switching accuracy, suggesting that greater metabolite increases were associated with smaller improvements in cognitive flexibility (45).

#### 3.4.4 Mood, arousal, and sleep-related outcomes

Two studies explicitly assessed mood-related outcomes in relation to MPMs (87, 88). In a randomized trial of healthy adults consuming peanut-based interventions, several hydroxybenzoic acid derivatives—including 3,4-dihydroxybenzoic acid sulfate (position not specified) (protocatechuic acid sulfate), 3-methoxybenzoic acid-4-sulfate, and 3,5-dimethoxy-4-(sulfooxy)benzoic acid—were inversely associated with anxiety and depression scores (88). Similarly, in a randomized blueberry intervention in healthy older adults, Curtis et al. (87) reported that postprandial calmness was positively associated with a hydroxycinnamic acid derivative, alongside broader cognitive benefits acutely and chronically (87). The same study also identified associations between MPMs and arousal-related outcomes: greater postprandial wakefulness was positively associated with 3-(4′-Hydroxy-3′-methoxyphenyl)propanoic acid and phenylacetic acid derivatives, whereas improved perceived sleep quality was associated with methoxylated phenylacetic acid metabolites (87).

#### 3.4.5 Neurodegeneration and pain

Two studies explored MPMs in relation to other neurological outcomes, such as neurodegenerative disease (90) or pain (91). In individuals with Parkinson’s disease (PD), urolithin metabotype profiles differed significantly from healthy controls, with a higher prevalence of non-producers (metabotype 0) and fewer urolithin A-only producers (metabotype A) among PD patients. These shifts were more pronounced with longer disease duration and greater severity, suggesting that gut microbial (poly)phenol metabolism may be altered during progressive neurodegeneration (90). A separate randomized controlled trial evaluated postoperative pain following supplementation with oak wood extract (Robuvit®). Urolithin metabotype A individuals experienced greater reductions in pain scores than non-producers, indicating that microbial capacity to generate urolithins may influence nociceptive processing (91).

### 3.5 Gastrointestinal and digestive health outcomes

Seven studies, including three crossover trials (67, 92, 93), two acute cross-over trials (51, 94) and one secondary analysis of an RCT (95), and one protocol for an RCT (77), assessed or expressed plans to assess gastrointestinal and digestive outcomes in relation to MPMs (Table 8). Sample sizes ranged from 27 to 54 participants. The populations for the studies ranged from healthy adults to older adults and from non-obese to overweight/obese, military, and those with active Crohn’s disease.

**Table 8:** Summary of studies assessing gastrointestinal or digestive health outcomes.

Reference	Study design	Population	Sample size (and intervention, if applicable)
Hidalgo-Liberona et al. (93)	RCT—Crossover	Older adults (≥60 yr) living in a residential care setting, with increased intestinal permeability	Total ((poly)phenol-rich snacks or control snacks): 51 Lower serum zonulin at baseline (LSZ): 26 Higher serum zonulin at baseline (HSZ): 25
Peron et al. (92)	RCT—Crossover	Older adults (≥60 yr) living in a residential care setting, with increased intestinal permeability	Total ((poly)phenol-rich snacks or control snacks): 51
Langley et al. (77)	Protocol for RCT	Adults with clinically active Crohn’s disease	Total: 32; Xanthohumol: 16; Control: 16
Karl et al. (95)	Secondary analysis of RCT	Healthy adults, including both military and civilians	Total: 54; Control diet: 27; Meal, Ready-to-Eat (MRE): 27
Meroño et al. (67)	RCT—Crossover	Older adults (≥60 yr) living in a residential care setting	Total ((poly)phenol-rich snacks or control snacks): 51
Nishioka et al. (94)	RCT—Acute Crossover	Women, obese or non-obese	Total: 27; Non-obese: 17; Obese: 10
Coelho et al. (51)	RCT—Acute Crossover	Adults with overweight/obesity	Total (Grape juice and placebo): 34

Included studies comprise randomized controlled trials (crossover and acute crossover designs), secondary analyses of randomized trials, and study protocols conducted in both healthy individuals and clinical populations. Sample sizes are reported as total participants and, where applicable, by intervention group or subgroup. RCT, randomized controlled trial; LSZ, lower serum zonulin; HSZ, higher serum zonulin; MRE, Meal, Ready-to-Eat.

Three publications included in this section were derived from the same participant cohort enrolled in the MaPLE trial (67, 92, 93) and are annotated as such in Tables 2–4. Although these studies utilized overlapping participant populations, they were retained separately because they reported distinct analyses and outcomes from separate research questions.

#### 3.5.1 Serum zonulin

Four studies assessed serum zonulin, a marker of intestinal permeability, three of which are derived from the MaPLE crossover trial. In this trial, older adults with increased intestinal permeability living in a residential care setting were given three (poly)phenol-rich snacks per day, or control snacks, for eightweeks (67, 92, 93). In Hidalgo-Liberona et al., participants were stratified by low versus high baseline serum zonulin concentrations (93). Individuals with lower baseline zonulin exhibited robust increases in MPMs following the intervention, whereas those with higher baseline zonulin showed attenuated metabolite responses despite identical dietary exposure. Although some flavan-3-ol phase II metabolites and phenyl-*γ*-valerolactone conjugates increased in the higher-zonulin group, overall metabolite changes were smaller than those observed in the lower-zonulin group. Furthermore, correlation analyses revealed serum zonulin levels were inversely correlated with several MPMs, including 3,4-dihydroxybenzoic acid-3-glucuronide and hydroxycinnamic acid-3′-glucuronide [*m*-coumaric acid glucuronide], in the lower baseline serum zonulin group; no significant correlations were observed in the higher-zonulin group (93).

Using MaPLE data, Peron et al. further reported associations between serum zonulin, age, circulating hippuric acid and 2-hydroxybenzene-1-sulfate [catechol sulfate], and gut microbiota features, including an inverse association between baseline 2-hydroxybenzene-1-sulfate and baseline zonulin (92). Meroño et al. observed reduced zonulin levels following the (poly)phenol-rich diet among individuals with urolithin metabotype B compared to metabotype A (67).

Finally, Nishioka et al. (94) conducted an acute RCT in non-obese and obese women with pasteurized orange juices obtained from *Citrus sinensis* L. cv. Pera and Moro (600 mL single dose) (94). No difference in zonulin and lipopolysaccharide was observed across metabolite excretion profiles.

#### 3.5.2 Additional intestinal permeability biomarkers

Karl et al. (95) assessed “Meals, Ready-to-Eat” (MRE) rations in healthy military and civilian adults (95), and observed an inverse association between 2-(4-hydroxyphenyl)propionate (along with caffeine alkaloids) and changes in intestinal permeability biomarkers, including claudin-3, glucagon-like peptide-2 (GLP-2), lipopolysaccharide-binding protein, and intestinal fatty acid-binding protein. These changes were also linked to shifts in microbiota composition. Langley et al. (77) published a protocol for a trial assessing xanthohumol supplementation in adults with clinically active Crohn’s disease (77). The study plans to examine intestinal permeability markers (e.g., CD14 and intestinal fatty acid-binding protein), markers of endotoxemia (i.e., LPS and LBP), and circulating xanthoumol metabolites.

#### 3.5.3 Appetite and satiety

Coelho et al. (51) presented data from two acute crossover trials in overweight/obese adults in evaluating Concord grape juice (CGJ; 355 mL), consumed either alone (Trial I) or with a standardized breakfast (Trial II), compared with phenolic-free control beverages (51). Trial 1 found that high urinary excreters of 3-hydroxyhippuric acid reported less fullness (satiety) after the Concord grape juice than low excreters. In trial 2 (beverage consumed with meal), higher urinary excretion of 4-hydroxybenzaldehyde was associated with lower desire to eat (appetite drive) after CGJ, but greater fullness (satiety) after the control beverage. Higher 3,4-dihydroxycinnamic acid sulfate (sulfation position not specified) [caffeic acid-O-sulfate] excretion was associated with reduced thirst and lower desire to eat.

### 3.6 Cancer outcomes

Four studies assessed relationships between cancer outcomes and MPMs, including two nested case–control studies, one RCT using walnuts or pomegranate juice as the intervention, and one single-arm open-label study using freeze-dried black raspberries as the intervention (n = 28-over 1700) (96–99) (Table 9). Studies included outcomes related to breast cancer, colorectal cancer, esophageal cancer, gastric cancer, or prostate cancer.

**Table 9:** Summary of studies assessing cancer outcomes.

Reference	Study design	Population	Sample size
Luo et al. (97)	Nested case–control	Adult women in China without breast cancer diagnosis at baseline	Total: 1053; Cases: 352; Controls: 701
Sun (98)	Nested case–control	Middle-aged Chinese males with and without gastric and esophageal cancers	Total: 1004; Gastric cancer: 190; Esophageal cancer: 42; Controls: 772
Pan et al. (99)	Single-arm	Patients with diagnosed colorectal cancer scheduled for surgical resection	Total (freeze-dried black raspberries): 28
González-Sarrías et al. (96)	RCT- Parallel	Adult males with prostate cancer or benign prostatic hyperplasia	Total: 63; Walnuts: 14; Pomegranate juice: 19; Controls (no intake): 30

Included studies comprise randomized controlled trials, nested case–control studies, and single-arm interventions conducted in populations with diagnosed cancers (e.g., prostate and colorectal cancer) or at risk of cancer development. Study populations include both clinical patients and observational cohorts without cancer at baseline. Sample sizes are reported as total participants and, where applicable, by case/control status or intervention group. RCT, randomized controlled trial.

#### 3.6.1 Breast cancer

A nested case–control study by Luo et al. (97) looked at associations with breast cancer. Although they did not find a direct relationship between MPMs and breast cancer risk, they found an inverse association between urinary epicatechin (a parent (poly)phenol) and breast cancer risk. The study also showed that levels of epicatechin and its microbial metabolites, phenyl-*γ*-valerolactones, were significantly higher among controls than their matched breast cancer cases. However, no statistically significant linear dose–response associations were found between urinary excretion of the metabolites and risk of breast cancer in categorized analyses.

#### 3.6.2 Colorectal cancer

Pan et al. (99) used freeze-dried black raspberries (60 g/day for 9 weeks) in colorectal cancer patients scheduled for surgical resection. Of the metabolites identified following the intervention, urinary and plasma 4-methylcatechol sulfate was positively correlated with the apoptotic marker (TUNEL) in the post-intervention tumors.

#### 3.6.3 Esophageal and gastric cancer

Sun et al. (98) conducted a large, nested case–control study in middle-aged Chinese males. The study observed no correlation between phenyl-γ-valerolactones and gastric or esophageal cancer at enrollment of the study. However, phenyl-γ-valerolactone derivatives showed a slight decrease in *H. pylori* seropositive participants, which is used to measure gastric cancer risk. Additionally, while the parent (poly)phenol epigallocatechin displayed an inverse association with the risk of gastric cancer alone and with gastric and esophageal cancer combined, this same association was not observed for MPMs.

#### 3.6.4 Prostate cancer

González-Sarrías et al. (96) conducted an RCT evaluating a walnut or pomegranate juice intervention in men with prostate cancer or benign prostatic hyperplasia. Ellagic acid and urolithin metabolites were detected in prostate tissue primarily among individuals classified as high excretors (i.e., high urinary excretion of ellagic acid and urolithin metabolites) whereas metabolites were largely undetected in low excretors and absent in control samples. However, metabolite presence in prostate tissue was not associated with clinical diagnosis, histological tissue type, pathological status (prostate cancer or benign prostatic hyperplasia), or expression of proliferation-related genes (CDKN1A, MKi-67, c-Myc).

### 3.7 Musculoskeletal outcomes

Greenbaum et al. (100) examined the relationship between MPMs and musculoskeletal health outcomes (Table 10). This cross-sectional study included 499 participants and evaluated the relationship between MPMs and bone mineral density (BMD) in healthy postmenopausal and perimenopausal women. Two significant positive correlations were reported between BMD and 3-phenylpropanoic and hippuric acid serum metabolites.

**Table 10:** Summary of study assessing epigenetic, musculoskeletal, or respiratory outcomes.

Reference	Study design	Population	Sample size
Yaskolka Meir et al. (101)	RCT—Three-arm	Adults with abdominal obesity and/or dyslipidemia	Total (Epigenetic—Li mAge): 294 Healthy Diet Guidelines: 88; MED Diet: 81; Green-MED Diet: 87
Greenbaum et al. (100)	Cross-sectional	Healthy post-menopausal and peri-menopausal females	Total (Musculoskeletal—BMD): 499; Postmenopausal: 419; Perimenopausal: 80
Cerdá et al. (80)	RCT—Parallel	Adults with stable COPD	Total (Respiratory): 30; Pomegranate juice: 15; Placebo: 15

Included studies comprise randomized controlled trials (parallel and multi-arm designs) and observational (cross-sectional) studies. Outcomes include epigenetic aging markers, bone mineral density, and respiratory function. Sample sizes are reported as total participants and, where applicable, by intervention group or subgroup. RCT, randomized controlled trial; MED, Mediterranean diet; BMD, bone mineral density; COPD, chronic obstructive pulmonary disease.

### 3.8 DNA regulation and epigenetic outcomes

Yaskolka Meir et al. (101) assessed the associations between urinary MPMs and DNA methylation-based biological aging through an 18-month RCT (Table 10). In participants following a (poly)phenol-rich, low-red/processed meat diet (Green-MED diet), urinary 2-(4′-hydroxyphenyl)ethanol (tyrosol) was independently associated with epigenetic age deceleration, as measured by Li mAge, even after adjustment for age, sex, and weight loss. 2-(3′-4′-dihydroxyphenyl)ethanol and urolithin C were inversely correlated with Li mAge in unadjusted analyses, but these associations were attenuated after multivariable adjustment.

### 3.9 Respiratory outcomes

One study, a randomized controlled trial by Cerdá et al. (80), assessed respiratory outcomes in response to a 5-week pomegranate juice intervention (400 mL/day) in patients with chronic obstructive pulmonary disease (COPD; Table 10). No significant associations were observed between urinary or plasma urolithins and respiratory function or clinical symptoms, including assessments by chest x-ray, spirometry, and arterial blood gas analysis.

## 4 Discussion

### 4.1 Overview

This scoping review mapped the current human evidence linking MPMs to host health outcomes and identified a literature base most concentrated in cardiometabolic research, followed by neurological and inflammatory domains. Comparatively fewer studies examined gastrointestinal and cancer outcomes, and only a single study each addressed musculoskeletal, epigenetic, and respiratory domains, highlighting these as areas for future research.

Across domains, MPMs were rarely associated with single isolated biomarkers; instead, recurring metabolite classes tended to align with clusters of related outcomes or phenotypes, particularly within cardiometabolic and cognitive domains. Benzoic acid, phenylpropanoic acid, phenylacetic acid, and related phenolic derivatives emerged repeatedly across studies, showing associations with adiposity, blood pressure, lipid metabolism, endothelial function, inflammatory markers, and memory-related outcomes. Urolithins also featured prominently; however, their associations were more context-dependent, often varying by metabotype classification, baseline health status, and specific endpoint assessed. The evidence base includes a mixture of randomized controlled trials, crossover interventions, single-arm studies, observational cohorts, and nested case–control analyses, reflecting both experimental and associative approaches to understanding MPM–health relationships. Collectively, the current literature suggests that microbial (poly)phenol metabolism is associated with broader physiological phenotypes or related outcomes rather than isolated biomarker effects.

### 4.2 Baseline factors impact MPM-outcome relationships

A recurring theme across multiple outcome domains was that the direction and detectability of MPM–health associations varied by baseline health status, biology, or producer phenotype. In healthy and at-risk populations within the cardiometabolic domain, urolithins and several phenolic acid derivatives more frequently aligned with favorable profiles across adiposity, blood pressure, vascular function, and composite risk measures (35, 40, 53, 57, 71). In contrast, in populations with established cardiometabolic conditions, signals were more often concentrated in lipid-related outcomes, while blood pressure, vascular, and glycemic findings were limited to fewer metabolite–outcome pairs and accompanied by more null results (61–64, 67, 68). In neurological contexts, shifts in urolithin metabotype distribution have been observed in Parkinson’s disease compared with healthy controls, with a higher prevalence of non-producers among individuals with longer disease duration and greater severity (90), suggesting that disease state itself may alter microbial (poly)phenol metabolism. Together, this pattern suggests that MPM associations may be more detectable in earlier or intermediate stages of risk, whereas advanced disease physiology and/or medication and metabolic dysregulation may attenuate, obscure, or complicate observable relationships.

Beyond health status, several studies suggest that underlying MPM producer phenotypes and baseline gut physiology may shape both metabolite exposure and observed associations. Urolithin metabotypes and related urolithins repeatedly differentiated cardiometabolic and gastrointestinal patterns more times than not, including differences in adiposity profiles across cohorts and differential changes in intestinal permeability markers following dietary intervention (35, 38, 40, 67). Notably, age-dependent variation in urolithin metabotype distribution was reported, with BMI–metabotype relationships observed only within specific age ranges (38), suggesting that life-stage biology may further modify ellagitannin metabolism. Host physiology may also influence systemic metabolite exposure: body mass index was shown to modify circulating gallotannin-derived metabolites following mango consumption (82), indicating that adiposity may shape both metabolite bioavailability and downstream associations. Similarly, in the MaPLE-derived analyses, baseline serum zonulin stratified metabolomic responses to a (poly)phenol-rich diet, with more robust microbial-derived metabolite increases and clearer inverse zonulin–metabolite relationships among individuals with lower baseline zonulin, but attenuated responses and fewer correlations among those with higher baseline zonulin (92, 93).

Multiple studies also support that the impacts of baseline producer or metabotype phenotypes extend beyond urolithins. Low anthocyanin metabolizer status was associated with higher blood pressure and less favorable arterial stiffness measures compared with higher metabolizer phenotypes in at-risk adults (52). Likewise, in an acute crossover trial in overweight/obese adults consuming Concord grape juice, appetite-related responses differed by urinary metabolite excretion strata (51). Collectively, these findings reinforce that exposure to bioactive MPMs is not determined by intake alone, and that baseline physiology and microbial metabolic capacity contribute to heterogeneity across human studies.

### 4.3 Domain-specific patterns

#### 4.3.1 Cardiometabolic outcomes

Cardiometabolic outcomes comprised the largest body of evidence, spanning 33 studies, one systematic review, and two active trials across healthy, at-risk, and clinical populations. Several consistent patterns emerged across anthropometric, vascular, lipid, and composite risk measures. First, urolithin metabotypes repeatedly stratified adiposity and metabolic profiles. Urolithin metabotype B was more frequently associated with higher BMI or overweight status (35, 38, 40) and less favorable baseline lipid profiles (49), whereas metabotype A aligned with more favorable body composition and greater weight loss in specific contexts (35, 40). At the individual metabolite level, urolithin A conjugates were inversely associated with waist circumference, visceral adiposity, insulin resistance markers, and select lipid parameters in at-risk and clinical populations (57, 62, 71), although glycemic endpoints were less consistently related (49, 62, 67).

Second, numerous phenolic acid derivatives showed consistent cross-endpoint associations, generally inverse with visceral fat (37, 44, 53), blood pressure in healthy adults (45), adverse lipid markers (53, 57), and composite cardiometabolic risk scores, including ASCVD risk and HeartScore (43, 55, 68). Although directionality varied, particularly for blood pressure in at-risk cohorts (53, 54, 56, 57), these metabolites repeatedly emerged across physiologically related metabolic and vascular domains. In healthy adults, most assessed MPMs were positively associated with flow-mediated dilation (36, 39, 41, 42, 45), suggesting enhanced nitric-oxide–dependent vasodilation, whereas associations in at-risk populations were more heterogeneous (54, 58, 65).

Finally, prospective and composite risk analyses suggest potential predictive value. Higher urinary excretion of select MPMs was associated with lower future type 2 diabetes risk (34, 55), while several urinary MPMs were inversely associated with estimated ASCVD risk and HeartScore in healthy adults (43). In addition, higher MPM scores aligned with more favorable composite cardiovascular health indices in clinical populations (68).

Mechanistically, the cardiometabolic associations observed for urolithins and phenolic acid derivatives are biologically plausible. Experimental evidence indicates that these metabolites modulate oxidative stress, enhance endothelial nitric oxide bioavailability, and influence lipid metabolism (102–106). Urolithins have also been linked to mitochondrial and lipid-related pathways (107, 108), supporting their recurring alignment with adiposity and lipid measures across human studies. Overall, MPMs—particularly urolithins and select phenolic acid derivatives—most consistently associate with adiposity, lipid metabolism, vascular function, and composite risk metrics, whereas glycemic endpoints and advanced clinical outcomes show greater heterogeneity, suggesting that associations may be most detectable in earlier or intermediate stages of cardiometabolic risk.

#### 4.3.2 Neurological outcomes

Within the neurological domain, associations were most consistent for cognitive endpoints—particularly episodic memory, executive function, and global cognition—across both observational and interventional designs (45, 87, 88). Urolithins, hydroxybenzoic acids, phenylacetic acids, and related derivatives were repeatedly linked to favorable cognitive performance, although not all trials demonstrated significant effects (86). Mood- and arousal-related outcomes were supported by fewer studies but suggested inverse relationships between hydroxybenzoic derivatives and anxiety or depression scores (88), alongside metabolite associations with calmness and wakefulness following blueberry supplementation (87). In Parkinson’s disease, altered urolithin metabotype distributions were observed compared with healthy controls, with greater prevalence of non-producers among those with longer disease duration (90).

These findings align with emerging evidence that microbial (poly)phenol metabolism may influence neuropsychological and psychophysiological processes via gut–brain axis pathways, potentially involving anti-inflammatory signaling, neurotransmitter modulation, or circadian regulation (109–112). However, ongoing studies—such as a recent trial assessing brain oxidative metabolism following urolithin A supplementation (113)—and future investigations incorporating neuroimaging, inflammatory markers, sleep assessments, and metabolomics alongside gut microbiota profiling will be critical for clarifying causal pathways.

#### 4.3.3 Inflammatory and oxidative stress outcomes

MPMs’ associations with inflammatory outcomes appeared more consistent for systemic inflammatory indices than for individual cytokines. Several phenolic acid derivatives were inversely associated with CRP and related composite inflammation indices (57, 75, 76), suggesting that higher circulating levels of certain MPMs may correspond to lower systemic inflammatory burden. In contrast, cytokine associations were metabolite or pathway-specific, with some MPMs positively associating with pro-inflammatory (73) and others with anti-inflammatory cytokines (72), and no associations being observed with urolithin metabotypes or circulating urolithins (67).

Both oxidative stress biomarkers and endotoxin-related markers (LPS and LBP) yielded mixed results. Whereas gallotannin-derived metabolites were inversely correlated with endotoxin following mango intervention (82), urolithins or urolithin metabotypes were not associated with LPS or LBP reductions (64, 81). While some flavanone-derived metabolites aligned with lower markers of oxidative liver stress (76), certain phenolic acids were positively associated with lipid peroxidation markers. In contrast, urolithins (114) and phenyl-*γ*-valerolactones (79) showed largely null associations across commonly measured systemic oxidative stress endpoints.

Although inflammatory associations were heterogeneous across endpoints, the observed patterns are biologically plausible given established evidence that several MPMs modulate central inflammatory and redox-sensitive signaling pathways. Mechanistic studies indicate that these metabolites can inhibit NF-κB and activate Nrf2, thereby influencing cytokine production and endogenous antioxidant defenses (104, 115–119). Although studies evaluating urolithin metabotypes within this review did not identify direct associations with circulating cytokines, controlled supplementation trials of urolithin A have demonstrated dose-dependent reductions in inflammatory markers, including IL-1β, CRP, IFN-γ, and TNF-*α* (25, 120). Together, these findings suggest that while endogenous metabolite exposure may variably associate with inflammatory endpoints in observational contexts, targeted modulation of specific metabolites could influence inflammatory signaling under controlled conditions.

#### 4.3.4 Gastrointestinal and digestive health outcomes

Evidence within gastrointestinal contexts was limited in volume but mechanistically coherent. Across multiple analyses, microbial phenolic metabolite profiles were differentially associated with markers of intestinal permeability, with stronger inverse relationships observed among individuals with lower baseline zonulin and attenuated associations in those with higher baseline permeability (67, 92, 93). Additional findings in military and civilian cohorts similarly linked specific MPMs to favorable changes in intestinal barrier–related biomarkers (95), while acute crossover studies indicated that postprandial appetite responses varied by urinary metabolite excretion phenotype (51). Collectively, these data suggest that inter-individual differences in microbial metabolite production may relate to intestinal barrier regulation and gastrointestinal signaling. This interpretation is supported by mechanistic evidence demonstrating that several (poly)phenol-derived metabolites enhance tight junction protein expression (e.g., occludin, claudin-1, ZO-1) and improve epithelial barrier integrity (121–123). Although human studies remain small and heterogeneous, the convergence of clinical and mechanistic data supports a plausible role for MPMs in modulating gut barrier function and postprandial physiology.

#### 4.3.5 Cancer outcomes

Evidence linkingMPMs to cancer outcomes was limited and heterogeneous across four studies (96–99). Across nested case–control analyses of breast, gastric, and esophageal cancers, MPMs were not consistently associated with cancer risk, and no clear dose–response relationships were observed (97, 98). In contrast, intervention data indicate that select microbial metabolites are bioavailable within tumor-relevant contexts: 4-methylcatechol sulfate correlated in colorectal cancer patients with tumor apoptotic activity following black raspberry supplementation (99), and urolithins were detectable in prostate tissue among high excretors following an ellagitannin-rich intervention (96), although without associations to clinical diagnosis, pathological status, or proliferation markers. Collectively, current human evidence does not demonstrate consistent relationships between MPMs and cancer incidence or progression; however, tissue-level detection and reported associations suggest that microbial metabolites may exert context-dependent biological effects in the tumor microenvironment that warrant further investigation.

#### 4.3.6 Other outcomes

Evidence in musculoskeletal, epigenetic, and respiratory domains remains limited to one study each, and thus patterns across studies were not elucidated. Notably, although only one study met our inclusion criteria within the musculoskeletal domain, several additional studies have investigated changes in musculoskeletal outcomes following direct supplementation with the microbial-derived metabolite urolithin A. These studies were not included in this review, as they did not assess relationships between circulating or excreted metabolite concentrations and health outcomes, but instead evaluated the effects of administered compounds. Nevertheless, they provide important insight into the translational potential of MPMs. Specifically, urolithin A supplementation has shown promise for enhancing mitochondrial and muscular function by upregulating key mitochondrial biogenesis and autophagy-related genes (e.g., CPT1B, GABA-RAPL1, FABP3, BECN1, PPGC1α, and PARK2) while reducing acylcarnitines, ceramides, and the systemic inflammatory marker CRP (120, 124). Urolithin A supplementation also increased muscle strength and endurance, although measures such as VO₂max (maximal aerobic capacity) and quadriceps strength were largely unchanged (25, 124).

#### 4.3.7 Methodological and analytical considerations

Several methodological and analytical considerations across the included studies should be considered when interpreting the current evidence base. First, many studies were exploratory, cross-sectional, secondary analyses, acute feeding studies, or small pilot trials, which may limit causal inference and increase susceptibility to reverse causation, residual dietary confounding, medication effects, lifestyle-related confounding, and microbiome-related confounding. However, some studies attempted to mitigate these factors through participant exclusions based on usage of antibiotics, fiber, pre/probiotics, and/or nutraceuticals, or dietary standardization strategies (Table 2). Nevertheless, the inconsistent implementation of these approaches across studies may still complicate interpretation of observed metabolite-health relationships.

Second, analytical heterogeneity was also evident across studies and may contribute to variability in reported findings. Included studies differed substantially with respect to metabolomics platforms, targeted versus untargeted analytical approaches, biological matrices, fasting versus postprandial sampling strategies, metabolite annotation methods, and quantification of conjugated versus unconjugated metabolites. Differences in normalization procedures, annotation confidence, analytical sensitivity, and availability of validated reference standards may further influence comparability and reproducibility across studies. However, one notable example of increasing methodological harmonization can be observed within the urolithin literature, where many studies have adopted similar dietary run-in procedures, intervention approaches, and analytical detection methods, thereby improving comparability across investigations. Development of similar standardized methodological frameworks for characterization of other microbial-derived polyphenol metabolites and food matrices may help strengthen reproducibility and translational applicability across future nutritional metabolomics research.

Third, an additional methodological limitation of included studies relates to the depth and consistency of microbiome characterization across studies. Although many studies discussed gut microbiota as a potential determinant of MPM production or metabotype status, only a subset formally assessed relationships between MPMs and microbiome features, and these analyses were most often limited to taxonomic profiling using 16S rRNA gene sequencing. Functional characterization through metagenomic approaches was comparatively uncommon. Consequently, the specific microbial taxa, metabolic pathways responsible for MPM production, and the impact of broader community dynamics remain incompletely characterized for many MPMs, limiting mechanistic interpretation and the development of microbiome-informed precision nutrition strategies.

Finally, many studies evaluated large numbers of metabolite-outcome associations simultaneously, which is an inherent challenge within metabolomics research and may increase susceptibility to false positive findings and overfitting, particularly in exploratory analyses. Although many studies applied multiplicity adjustment or false discovery rate correction procedures and the current review preferentially reported corrected findings when available, differences in statistical approaches and reporting practices may still complicate interpretation across studies. Accordingly, while the reviewed literature supports potential associations between microbial-derived polyphenol metabolites and health outcomes, many findings should currently be interpreted as exploratory or hypothesis-generating.

#### 4.3.8 Strengths and limitations

To our knowledge, this is among the first reviews to systematically evaluate human studies linking specific MPMs to health and disease outcomes across multiple clinical domains. All articles included in this review were conducted in humans, eliminating indirect inference from animal or *in vitro* models and ensuring direct translational relevance for clinical practice and precision nutrition. Another notable strength is that we included studies of all sample sizes, durations (including acute and single administration), and age groups, providing insights into the role of these metabolites under various conditions. However, while appropriate for a scoping review, it also presents limitations. The highly heterogeneous evidence base with respect to metabolite panels, biological matrices, analytical platforms, intervention doses and durations, and participant health status, complicated direct comparison across studies. Additionally, reporting of metabotypes, metabolite cutoffs, background diet, and microbiome composition is inconsistent in many studies, limiting the ability to disentangle diet–microbiome–host interactions or to generalize across populations. Furthermore, as previously highlighted by Hazim et al. (125) it remains uncertain whether the reported health outcomes are mediated by individual phenolic metabolites directly or are indicative of broader host-specific phenolic metabotypes shaped by gut microbial composition and function.

Another important consideration is that this review intentionally included not only confirmed MPMs, but also host–microbial co-metabolites and metabolites with potential mixed or uncertain origins. This broader framework allowed for a more comprehensive assessment of metabolite signatures potentially influenced by gut microbial composition and metabolic function, including metabolites whose bioavailability, transformation, or biological activity may still depend on host–microbe interactions despite additional contributions from diet or endogenous host metabolism. However, this approach also represents a limitation, as the biological origin of several included metabolites cannot always be definitively attributed to gut microbial metabolism alone. Consequently, some reported associations may reflect overlapping contributions from dietary exposure, host digestion and metabolism, microbial biotransformation, and/or broader lifestyle factors, thereby complicating mechanistic interpretation and causal inference.

#### 4.3.9 Gaps and future directions

A major gap remains in the ability to compare findings across metabolite classes and study designs, particularly for those that do not use a reproducible metabotype framework. While the results that were most comparable across studies included in this review were those reporting metabotype–outcome relationships, these approaches were largely limited to well-characterized metabotypes such as urolithin and equol producer status. In contrast, many studies evaluating flavonoids and phenolic acids reported associations using individual metabolite concentrations at single timepoints or as pre–post changes, or stratified participants using study-specific quantiles (e.g., tertiles of circulating or urinary concentrations), limiting comparability across studies. As emerging evidence suggests that metabotypes themselves, beyond concentrations of individual metabolites, relate to health outcomes, future research should prioritize the development of reproducible and standardized metabotyping frameworks for a broader range of MPMs, including phenolic acids and flavonoids.

An additional gap is the limited integration of baseline gut microbiome composition and functional capacity with metabolite measurements. Few studies concurrently assess baseline microbiome features, functional potential, and metabolite output, restricting the ability to link metabolite production to underlying microbial ecology or to identify microbial signatures that predict high- or low-producer phenotypes and associated health outcomes. To date, such integrated analyses have been most extensively studied in the context of well-characterized urolithin and equol metabotypes, with comparatively limited investigation across other (poly)phenol classes. Addressing this gap will be essential for advancing prediction models of metabolite exposure and for developing targeted interventions aimed at modulating or overcoming inter-individual differences in gut microbial metabolism to achieve consistent physiological responses.

From a translational perspective, a key question is whether direct supplementation with specific MPMs can overcome inter-individual variability in microbial metabolic capacity and achieve health outcomes in individuals with low endogenous production. This approach has begun to be explored for select metabolites, most notably urolithin A and equol, which also represent metabolites from the most well-established metabotype frameworks. While early findings are promising, they remain limited, and these examples highlight a broader challenge: advancing this strategy for other (poly)phenol classes will require first addressing the upstream gaps in defining *reproducible* metabolite-based phenotypes and linking them to underlying microbiome features and clinical outcomes. Even within these established models, key uncertainties remain that should be evaluated in future studies, including whether supplementation can reproducibly replace endogenous microbial production, shift the gut microbiome toward a producer phenotype, or recapitulate the complex and dynamic mixture of metabolites generated *in vivo*.

## 5 Conclusion

This scoping review systematically mapped the current human literature evaluating associations between MPMs and health- and disease-related outcomes, with the goal of clarifying whether specific downstream metabolites, rather than parent (poly)phenols alone, relate to measurable host physiology. Although the number of human studies is expanding, evidence remains unevenly distributed across domains, with the strongest concentration in cardiometabolic outcomes and more limited data in inflammatory, neurological, gastrointestinal, and cancer contexts, and even more limited data in musculoskeletal, epigenetic, and respiratory contexts. Across domains, recurrent associations involving urolithins and select phenolic acid derivatives suggest that specific microbial metabolites may underlie inter-individual differences in diet–health responses. Importantly, these findings highlight a central translational implication: identifying which specific MPMs reproducibly associate with defined clinical endpoints may help disentangle variability arising from producer phenotype or microbial metabolic capacity. Future research should determine whether targeted augmentation of specific metabolites, particularly in individuals who are low or non-producers, can reproduce associations observed in high-producer phenotypes. Prospective and controlled intervention studies incorporating standardized metabolite quantification, repeated sampling, baseline microbiome assessment, and phenotype stratification will be essential to determine how variability in microbial metabolite production can be overcome to achieve consistent physiological responses across individuals. Such work may ultimately inform precision nutrition strategies aimed at bypassing inter-individual variability in microbial metabolism to optimize health outcomes.
