# Correction: Clinical evidence for microbial-derived polyphenol metabolites in health and disease: a scoping review

**DOI:** 10.3389/fnut.2026.1993867

**Published:** 2026-09-24

**Authors:** Jennie Brown, Lydia Norby-Adams, Nancy Ghanem, Ayda Lewis, Joshua Z. Goldenberg, Tiffany Weir, Alexandra Adorno Vita

**Affiliations:** 1 Helfgott Research Institute, National University of Natural Medicine, Portland, OR, United States; 2 Department of Food Science and Human Nutrition, Colorado State University, Fort Collins, CO, United States; 3 Department of Cell and Molecular Biology, Colorado State University, Fort Collins, CO, United States

**Keywords:** cardiometabolic outcomes, dietary (poly)phenols, gut microbiome, metabotypes, microbial-derived (poly)phenol metabolites, precision nutrition, urolithins

The funder “American Heart Association” to Nancy Ghanem was erroneously omitted. The correct **Funding** statement reads:

“The author(s) declared that financial support was received for this work and/or its publication. AV and TW are currently supported by the National Institutes of Health via grant 1RM1AT013318–01 (NCCIH); AV received additional support via 5R90AT008924–09 (NCCIH) and Colorado State University's College of Health and Human Sciences Seed Grant Program. JG is supported by the National Institutes of Health via grants 5R90AT008924–09 (NCCIH) and 1R25DK130848–01 (NIDDK) and a RAND REACH Center pilot research grant. NG was supported by American Heart Association Grant #25PRE1374125 / 2025–2026”

The original version of this article has been updated.

