# Supplementary material for: Clinical evidence for microbial-derived polyphenol metabolites in health and disease: a scoping review
Source: Front Nutr. 2026 Jun 17;13:1859472. doi: 10.3389/fnut.2026.1859472 (PMC13319019; doi:10.3389/fnut.2026.1859472)
Supplement: Supplementary file 1 [file Supplementary_file_1.DOCX]

Supplemental File 1. PubMed Search Strategy

(coumarins[Mesh:NoExp] OR flavonoids[Mesh:NoExp] OR "hydrolyzable tannins"[Mesh:NoExp] OR lignans[Mesh:NoExp] OR polyphenols[Mesh] OR polyphenols/metabolism[Mesh:NoExp] OR stilbenes[Mesh:NoExp] OR tannins[Mesh:NoExp] OR bioflavonoid[tiab] OR bioflavonoids[tiab] OR coumarin[tiab] OR coumarine[tiab] OR coumarines[tiab] OR coumarins[tiab] OR "ellagi tannins"[tiab] OR ellagitannins[tiab] OR flavonoid[tiab] OR flavonoids[tiab] OR "gallo tannins"[tiab] OR gallotannins[tiab] OR "hydrolysable tannins"[tiab] OR "hydrolyzable tannins"[tiab] OR lignan[tiab] OR lignans[tiab] OR polyphenol[tiab] OR polyphenols[tiab] OR "pyrogallol tannins"[tiab] OR resveratrol[tiab] OR stilbene[tiab] OR stilbenes[tiab] OR stilbenoid[tiab] OR stilbenoids[tiab] OR tannin[tiab] OR tannins[tiab] OR urolithin[tiab] OR urolithins[tiab]) AND (microbiota[Mesh:NoExp] OR bacteria[Mesh:NoExp] OR biofilms[Mesh:NoExp] OR "gastrointestinal microbiome"[Mesh:NoExp] OR "microbial consortia"[Mesh:NoExp] OR bacteria[tiab] OR bacterial[tiab] OR microbe[tiab] OR microbes[tiab] OR microbial[tiab] OR microbiome[tiab] OR microbiomes[tiab] OR microbiota[tiab] OR "microbial metabolites"[all] OR "microbial-derived metabolites"[all] OR "bacterial metabolites"[all] OR "bacterial-derived metabolites"[all] OR "microbial metabolism"[tiab] OR feces/microbiology[Mesh:NoExp])AND (metabolism[Mesh:NoExp] OR "Metabolism"[Mesh] OR metabolic[tiab] OR metabolism[tiab] OR metabolisms[tiab] OR metabolite[tiab] OR metabolites[tiab]) AND(humans[Mesh])
