# Supplementary material for: Clinical evidence for microbial-derived polyphenol metabolites in health and disease: a scoping review
Source: Front Nutr. 2026 Jun 17;13:1859472. doi: 10.3389/fnut.2026.1859472 (PMC13319019; doi:10.3389/fnut.2026.1859472)
Supplement: Supplementary file 2 [file Table_1.DOCX]

**Table of Contents for Supplementary Tables S1A-H**

Supplementary Table S1A. Relationships between cardiometabolic outcomes and MPMs – Healthy Populations……………..Page 2

Supplementary Table S1B. Relationships between cardiometabolic outcomes and MPMs – At-Risk Populations…………….Page 4

Supplementary Table S1C. Relationships between cardiometabolic outcomes and MPMs – Existing Conditions……….….…Page 8

Supplementary Table S1D. Relationships between immunological or oxidative outcomes and MPMs ………………………..Page 9

Supplementary Table S1E. Relationships between neurological outcomes and MPMs …………………………………….…Page 11

Supplementary Table S1F. Relationships between gastrointestinal or digestive health outcomes and MPMs ………….……..Page 13

Supplementary Table S1G. Relationships between cancer outcomes and MPMs ……………………………………………...Page 14

Supplementary Table S1H. Relationships between epigenetic, musculoskeletal, or respiratory outcomes and MPMs …...…..Page 14

Supplementary Table S1A. Relationships between cardiometabolic outcomes and MPMs – Healthy Populations

|  | **Healthy** |  |
| --- | --- | --- |
| **Outcome** | **Result** | **Reference** |
| **Vascular Function** | |  |
| FMD | ↑ (4R)-5-(3′-hydroxyphenyl)-γ-valerolactone-4′-O-sulfate ↑ 1-methylpyrogallol-O-sulfate ↑ 2,3-dihydroxybenzene-1-sulfate (pyrogallol-O-sulfate) ↑ 2,4-dihydroxybenzoic acid ↑ 2-hydroxybenzoic acid ↑ 2-hydroxyhippuric acid ↑ 3,4-dihydroxyphenylacetic acid ↑ 3-caffeoylquinic acid ↑ 3-feruloylquinic acid ↑ 3-hydroxy-4'-methoxycinnamic acid (isoferulic acid) ↑ 3-hydroxybenzoic acid ↑ 3-hydroxyhippuric acid ↑ 3-hydroxyphenyl acetic acid ↑ 4-caffeoylquinic acid ↑ 4-feruloylqunic acid ↑ 4-hydroxybenzaldehyde ↑ 4-hydroxybenzoic acid ↑ 4-hydroxyphenyl acetic acid ↑ 4-methoxycinnamic acid ↑ 4-methylcatechol-2-O-sulfate ↑ 4-methylgallic acid-3-O-sulfate ↑ 5-feruloylquinic acid ↑ Caffeic-3'-O-sulfate ↑ Caffeic-4'-O-sulfate ↑ Catechol-O-sulfate ↑ Chlorogenic acid ↑ Cinnamic acid ↑ Dihydrocaffeic acid-3-O-sulfate ↑ Dihydroferulic acid ↑ Dihydroferulic acid-4′-O-β-d-glucuronide ↑ Dihydro-isoferulic acid ↑ Dihydro-isoferulic acid-3′-O-sulfate ↑ Dihydro-isoferulic acid-3′-O-β-d-glucuronide ↑ Ferulic acid ↑ Ferulic acid-4′-O-sulfate ↑ Ferulic acid-4′-O-β-glucuronide ↑ Gallic acid ↑ Hippuric acid ↑ Homovanillic acid ↑ Isoferulic acid ↑ Isoferulic acid-3′-O-sulfate ↑ Isoferulic acid-3′-O-β-d-glucuronide ↑ Isovanillic acid ↑ m-coumaric acid-3'-O-sulfate ↑ Methylferulic acid ↑ o-coumaric acid ↑ p-coumaric acid ↑ Phenylacetic acid ↑ Protocatechuic acid ↑ Syringic acid ↑ UA-sulfate ↑ Urolithin A (UA)-gucuronide ↑ Vanillic acid ↑ Vanillic acid-4′-O-sulfate ↓ 2,3,4-trihydroxybenzoic acid ↓ 2-hydroxybenzoic acid ↓ 2'-hydroxyhippuric acid ↓ Phenylacetic acid | Istas et al. 2019 Rodriguez-Mateos et al. 2019 Wood et al. 2023 Rodriguez-Mateos et al. 2019 Istas et al. 2019 Istas et al. 2019 Istas et al. 2019 Mills et al. 2017 Mills et al. 2017 Wood et al. 2023 Istas et al. 2019 Rodriguez-Mateos et al. 2019 Rodriguez-Mateos et al. 2019 Mills et al. 2017 Mills et al. 2017 Istas et al. 2019 Istas et al. 2019, Rodriguez-Mateos et al. 2019 Rodriguez-Mateos et al. 2019 Mills et al. 2017 Rodriguez-Mateos et al. 2019 Rodriguez-Mateos et al. 2019 Mills et al. 2017 Mills et al. 2017 Mills et al. 2017 Istas et al. 2019 Rodriguez-Mateos et al. 2019 Rodriguez-Mateos et al. 2019 Rodriguez-Mateos et al. 2019 Istas et al. 2019, Rodriguez-Mateos et al. 2019 Istas et al. 2019, Rodriguez-Mateos et al. 2019 Istas et al. 2019 Istas et al. 2019 Istas et al. 2019, Rodriguez-Mateos et al. 2019 Mills et al. 2017, Rodriguez-Mateos et al. 2019 Istas et al. 2019, Mills et al. 2017, Rodriguez-Mateos et al. 2019 Mills et al. 2017, Rodriguez-Mateos et al. 2019 Istas et al. 2019 Rodriguez-Mateos et al. 2019 Rodriguez-Mateos et al. 2019 Mills et al. 2017 Mills et al. 2017 Istas et al. 2019, Mills et al. 2017, Rodriguez-Mateos et al. 2019 Istas et al. 2019 Mills et al. 2017 Mills et al. 2017,  Istas et al. 2019 Istas et al. 2019, Rodriguez-Mateos et al. 2019 Istas et al. 2019 Istas et al. 2019, Rodriguez-Mateos et al. 2019 Rodriguez-Mateos et al. 2019 Istas et al. 2018 Istas et al. 2018 Rodriguez-Mateos et al. 2019 Istas et al. 2019 Wood et al. 2023 Wood et al. 2023 Wood et al. 2023 Wood et al. 2023 |
| **Adiposity/Body Composition** | | |
| VAT | ↓ 4-methylcatechol sulfate ↓ Hippurate ↓ UMA vs. UM-B, UM-0 | Mostafa et al. 2023 Pallister et al. 2017 Cortés-Martín et al. 2019 |
| WC | ↓ UM-A vs. UM-B, UM-0 | Cortés-Martín et al. 2019 |
| BMI | ↑ 8-prenylnaringen ↑ UM-B vs. UM-A ↓ UM-A vs. UM-B, UM-0 | Jamieson et al. 2024  Selma et al. 2016  Cortés-Martín et al. 2019 |
| **Blood Pressure/Hemodynamics** | | |
| SBP | ↓ 2-hydroxy-4-methylbenzene-1-sulfate  ↓ 3(2′,4′-dihydroxyphenyl)propanoic acid ↓ 3-(2′-hydroxyphenyl)propanoic acid ↓ 3-(3′,5′-dihydroxyphenyl)propanoic acid ↓ 3′-hydroxyhippuric acid ↓ 3-methoxybenzoic acid-4-sulfate (vanillic acid-4-O-sulfate) ↓ 4′-hydroxyhippuric acid ↓ 4′-methoxycinnamic acid-3′-sulfate (isoferulic acid 3-O-sulfate) ↓ Benzoic acid ↓ Cinnamic acid ↓ Hippuric acid | Wood et al. 2023 Wood et al. 2023 Wood et al. 2023 Wood et al. 2023 Wood et al. 2023 Wood et al. 2023 Wood et al. 2023 Wood et al. 2023 Wood et al. 2023 Wood et al. 2023 Wood et al. 2023 |
| **Disease Risk, Aggregate Risk Scores, and other Composite Measures** | | |
| T2DM Risk | ↓ Caffeic acid ↓ Hesperetin ↓ Naringenin | Sun et al. 2015 Sun et al. 2015 Sun et al. 2015 |
| ASCVD risk score | ↓ 2-hydroxycinnamic acid ↓ 3-(2,4-dihydroxyphenyl)propanoic acid ↓ 3-(3,4-dihydroxyphenyl)propanoic acid ↓ 3-(3,5-dihydroxyphenyl)propanoic acid ↓ 3,5-dihydroxybenzoic acid ↓ 3-hydroxyphenylethanol-4-sulfate ↓ 4-hydroxyphenylethanol-3-sulfate ↓ Total phenylpropanoic acids | Li et al. 2023 Li et al. 2023 Li et al. 2023 Li et al. 2023 Li et al. 2023 Li et al. 2023 Li et al. 2023 Li et al. 2023 |
| Heartscore | ↑ 3-(3,4-Dihydroxyphenyl)propanoic acid ↑ 3-(3,5-Dihydroxyphenyl)propanoic acid ↑ 3-(4-Methoxypehnyl)propanoic acid-3-sulfate ↑ 3,5-dihydroxybenzoic acid ↑ 3-hydroxybenzoic acid ↑ 4-Hydroxyhippuric acid ↓ 2,4-Dihydroxybenzoic acid ↓ 2,6-Dihydroxybenzene-1-sulfate ↓ 2,6-Dihydroxybenzoic acid ↓ 2-hydroxycinnamic acid ↓ 3,4,5-trihydroxybenzoic acid | Li et al. 2023 Li et al. 2023 Li et al. 2023 Li et al. 2023 Li et al. 2023 Li et al. 2023 Li et al. 2023 Li et al. 2023 Li et al. 2023 Li et al. 2023 Li et al. 2023 |
| **Null Findings or Planned Assessments** | |  |
| Null Findings | × Iso-Urolithin-A (glucuronide) ⊥ AIX × Iso-Urolithin-A (glucuronide) ⊥ PWV × Proanthocyanidin metabolites ⊥ BMI  × Total Urolithins ⊥ AIX × Total Urolithins ⊥ PWV × Urolithin-A (glucuronide, sulfate)⊥ AIX × Urolithin-A metabotype⊥ BMI × Urolithin-B (glucuronide, sulfate)⊥ AIX × Urolithin-B (glucuronide, sulfate)⊥ PWV | Istas et al. 2018 Istas et al. 2018 Cortés-Martín et al., 2018a Istas et al. 2018 Istas et al. 2018 Istas et al. 2018 Cortés-Martín et al., 2018a  Istas et al. 2018 Istas et al. 2018 |
| Planned assessment | Urolithin formation ↔ Cholesterol status Urolithin formation ↔ Lipids | NCT03713164 NCT03713164 |
| **Key:**  Outcome abbreviations: (FMD) Flow-mediated dilation, (D₀) Pre-occlusion vessel diameter, (PWV) Pulse wave velocity, (AIX) Aortic augmentation index, (VAT) Visceral adipose tissue (visceral fat mass), (WC) Waist circumference, (BP) Blood pressure, (SBP) Systolic blood pressure, (DBP) Diastolic blood pressure, (MAP) Mean arterial pressure, (BG) Blood glucose, (TG) Triglycerides, (HDL-c) High-density lipoprotein cholesterol, (XL-HDL-P) Extra-large high-density lipoprotein particles, (LDL-c) Low-density lipoprotein cholesterol, (HbA1c) Hemoglobin A1c, (PreDM-IR) Prediabetes with insulin resistance, (T2DM) Type 2 diabetes mellitus, (ICVH) Ideal cardiovascular health, (ASCVD) Atherosclerotic cardiovascular disease  Low Anthocyanin Metabolizers: Measured by metabolism of hippuric acid, 3-hydroxyhippuric acid, 4-hydroxy-3-methoxyphenylacetic acid, and 3,5dihydroxyphenylpropionic acid  ↑ Shows positive correlation ↓ Shows inverse correlation × Shows no correlation with ⊥ showing statistical independence ↔ Relationship to be investigated: upcoming or ongoing trial | | |

Supplementary Table S1B. Relationships between cardiometabolic outcomes and MPMs – At-risk Populations

| **At-Risk** |  |  |
| --- | --- | --- |
| **Outcome** | **Result** | **Reference** |
| **Vascular Function** | |  |
| FMD | ↑ 3-(4′-hydroxyphenyl)propanoic acid-3′-glucuronide ↑ 3-hydroxybenzoic acid-4-sulfate ↓ 2-hydroxy-3-(4′-hydroxyphenyl)propanoic acid ↓ 3-(2′,4′-dihydroxyphenyl)propanoic acid ↓ 3,5-dihydroxybenzoic acid ↓ 3′-hydroxyphenylacetic acid ↓ 3-hydroxy-4-methoxybenzoic acid-5-sulfate ↓ 3-hydroxybenzoic acid (placebo) ↓ 4-hydroxybenzoic acid (placebo) ↓ 4-hydroxyhippuric acid (placebo) ↑ 3-(4-methoxyphenyl)propanoic acid 3-O-glucuronide | Woolf et al. 2023 Woolf et al. 2023 Woolf et al. 2023 Woolf et al. 2023 Woolf et al. 2023 Woolf et al. 2023 Woolf et al. 2023 Woolf et al. 2023 Woolf et al. 2023 Woolf et al. 2023 Huang et al. 2021 |
| D₀ | ↓ 3-methylhippuric acid | Huang et al. 2021 |
| PWV | ↑ 3-hydroxy-2-methoxybenzene-1-sulfate ↓ (4R)-5-(3’-hydroxyphenyl)-γ-valerolactone-4’-sulfate ↓ 2-(4’-hydroxyphenoxy)propanoic acid ↓ 2,4-dihydroxybenzoic acid ↓ 2,6-dihydroxybenzene-1-sulfate ↓ 2,6-dihydroxybenzoic acid ↓ 2’-hydroxyhippuric acid ↓ 2-hydroxy-4-methylbenzene-1-sulfate ↓ 2-hydroxy-6-methoxybenzene-1-sulfate ↓ 2-hydroxybenzene-1-glucuronide ↓ 2-hydroxybenzoic acid ↓ 3-(2’,3’-dihydroxyphenyl)propanoic acid ↓ 3-(2’-hydroxyphenyl)propanoic acid ↓ 3-(3’,4’-dihydroxyphenyl)propanoic acid ↓ 3,4-dihydroxybenzaldehyde ↓ 3,4-dihydroxybenzoic acid ↓ 3’,4’-dihydroxycinnamic acid ↓ 3’-hydroxycinnamic acid-4’-glucuronide ↓ 3’-hydroxyhippuric acid ↓ 3’-hydroxyphenylacetic acid ↓ 3’-methoxycinnamic acid-4’-glucuronide ↓ 3’-methoxycinnamic acid-4’-sulfate ↓ 3-hydroxybenzoic acid-4-sulfate ↓ 3-O-feruloylquinic acid ↓ 4’-hydroxycinnamic acid ↓ 4’-hydroxycinnamic acid-3’-glucuronide ↓ 4’-hydroxycinnamic acid-3’-sulfate ↓ 4’-hydroxycinnamic acid-4’-sulfate ↓ 4’-methoxycinnamic acid-3’-glucuronide ↓ 4-hydroxybenzoic acid ↓ 4-hydroxybenzoic acid-3-glucuronide ↓ 4-hydroxybenzoic acid-3-sulfate ↓ 4-O-Caffeoylquinic acid ↓ 5-O-Caffeoylquinic acid ↓ Hippuric acid | Le Sayec et al. 2022 Le Sayec et al. 2022 Le Sayec et al. 2022 Le Sayec et al. 2022 Le Sayec et al. 2022 Le Sayec et al. 2022 Le Sayec et al. 2022 Le Sayec et al. 2022 Le Sayec et al. 2022 Le Sayec et al. 2022 Le Sayec et al. 2022 Le Sayec et al. 2022 Le Sayec et al. 2022 Le Sayec et al. 2022 Le Sayec et al. 2022 Le Sayec et al. 2022 Le Sayec et al. 2022 Le Sayec et al. 2022 Le Sayec et al. 2022 Le Sayec et al. 2022 Le Sayec et al. 2022 Le Sayec et al. 2022 Le Sayec et al. 2022 Le Sayec et al. 2022 Le Sayec et al. 2022 Le Sayec et al. 2022 Le Sayec et al. 2022 Le Sayec et al. 2022 Le Sayec et al. 2022 Le Sayec et al. 2022 Le Sayec et al. 2022 Le Sayec et al. 2022 Le Sayec et al. 2022 Le Sayec et al. 2022 Le Sayec et al. 2022 |
| **AIX** | ↑ Low Anthocyanin Metabolisers | Flynn 2021 |
| awake AIXao | ↑ 2,4-dihydroxybenzoic acid ↑ 2,6-dihydroxybenzoic acid ↑ 2-hydroxybenzoic acid ↑ 3,4-dihydroxybenzaldehyde ↓ (4R)-5-(3'-hydroxyphenyl)-ϒ-valerolactone-4'-sulfate ↓ 2,6-dihydroxybenzene-1-sulfate ↓ 2-hydroxybenzene-1-glucuronide ↓ 3-(3'-methoxyphenyl)propanoic acid-4'-glucuronide ↓ 3-(3'-methoxyphenyl)propanoic acid-4'-sulfate ↓ 3-(4'-hydroxyphenyl)propanoic acid-3'-sulfate ↓ 3-(4'-Methoxyphenyl)propanoic acid-3'-sulfate ↓ 3,4,5-trihydroxybenzene ethyl ester ↓ 3,4,5-trihydroxybenzoic acid ↓ 3,4-dihydroxybenzoic acid ↓ 3-O-Feruloylquinic acid ↓ 4-hydroxybenzoic acid-3-glucuronide ↓ 4-hydroxybenzoic acid-3-sulfate | Le Sayec et al. 2022 Le Sayec et al. 2022 Le Sayec et al. 2022 Le Sayec et al. 2022 Le Sayec et al. 2022 Le Sayec et al. 2022 Le Sayec et al. 2022 Le Sayec et al. 2022 Le Sayec et al. 2022 Le Sayec et al. 2022 Le Sayec et al. 2022 Le Sayec et al. 2022 Le Sayec et al. 2022 Le Sayec et al. 2022 Le Sayec et al. 2022 Le Sayec et al. 2022 Le Sayec et al. 2022 |
| 24hr AIXao | ↑ 2,4-dihydroxybenzoic acid ↑ 2,6-dihydroxybenzoic acid ↑ 3,4-dihydroxybenzaldehyde ↓ (4R)5(3'-hydroxyphenyl)-y-valerolactone-4'-sulfate ↓ 3,4,5-trihydroxybenzene ethyl ester ↓ 4-hydroxy-3,5-dimethoxybenzoic acid ↓ 4-hydroxybenzoic acid-3-glucuronide ↓ 4-hydroxybenzoic acid-3-sulfate ↓ 4-methoxybenzoic acid-3-sulfate | Le Sayec et al. 2022 Le Sayec et al. 2022 Le Sayec et al. 2022 Le Sayec et al. 2022 Le Sayec et al. 2022 Le Sayec et al. 2022 Le Sayec et al. 2022 Le Sayec et al. 2022 Le Sayec et al. 2022 |
| awake AIXbr | ↑ 2,4-dihydroxybenzoic acid ↑ 2,6-dihydroxybenzoic acid ↑ 2-hydroxybenzoic acid ↑ 3,5-dihyroxybenzoic acid ↓ 2-hydroxybenzene-1-glucuronide ↓ 3-(4'-hydroxyphenyl)propanoic acid-3'-sulfate ↓ 3-(4'-Methoxyphenyl)propanoic acid-3'-sulfate ↓ 3,4-dihydroxybenzoic acid ↓ 3-O-Feruloylquinic acid ↓ 4-hydroxybenzoic acid-3-glucuronide ↓ 4-hydroxybenzoic acid-3-sulfate | Le Sayec et al. 2022 Le Sayec et al. 2022 Le Sayec et al. 2022 Le Sayec et al. 2022 Le Sayec et al. 2022 Le Sayec et al. 2022 Le Sayec et al. 2022 Le Sayec et al. 2022 Le Sayec et al. 2022 Le Sayec et al. 2022 Le Sayec et al. 2022 |
| 24hr AIXbr | ↑ 2,4-dihydroxybenzoic acid ↑ 2,6-dihydroxybenzoic acid ↓ 3,4,5-trihydroxybenzene ethyl ester ↓ 4-hydroxy-3,5-dimethoxybenzoic acid ↓ 4-hydroxybenzoic acid-3-glucuronide ↓ 4-methoxybenzoic acid-3-sulfate | Le Sayec et al. 2022 Le Sayec et al. 2022 Le Sayec et al. 2022 Le Sayec et al. 2022 Le Sayec et al. 2022 Le Sayec et al. 2022 |
| **Adiposity/Body Composition** | | |
| VAT | ↓ Urolithin A  ↓ Hippuric acid ↓ Gallic acid ↓ Gallic acid aglycone ↓ Gallic acid glucuronide ↓ Gallic acid sulfate ↓ Urolithin B ↓ Urolithin B aglycone ↓ Urolithin B glucuronide ↓ Urolithin B sulfate | Zelicha et al. 2022  Zelicha et al. 2022 Laveriano-Santos et al. 2022 Laveriano-Santos et al. 2022 Laveriano-Santos et al. 2022 Laveriano-Santos et al. 2022 Laveriano-Santos et al. 2022 Laveriano-Santos et al. 2022 Laveriano-Santos et al. 2022 Laveriano-Santos et al. 2022 |
| Abdominal Obesity (WC ≥ 90th percentile) | ↓ Gallic acid ↓ Gallic acid aglycone ↓ Gallic acid glucuronide ↓ Gallic acid sulfate | Laveriano-Santos et al. 2022 Laveriano-Santos et al. 2022 Laveriano-Santos et al. 2022 Laveriano-Santos et al. 2022 |
| WC | ↓ 2,6-dihydroxybenzoic acid ↓ 2-hydroxyphenylacetic acid ↓ 3-(2-hydroxyphenyl)propionic acid ↓ 3,4-dihydroxybenzoic acid ↓ Cinnamoylglycine ↓ Pyrogallol sulfate 1 ↓ Urolithin A glucuronide ↓ Urolithin A sulfate ↓ Gallic acid ↓ Gallic acid aglycone ↓ Gallic acid glucuronide ↓ Gallic acid sulfate ↓ Urolithin B ↓ Urolithin B aglycone ↓ Urolithin B glucuronide ↓ Urolithin B sulfate | Lanuza et al. 2023 Lanuza et al. 2023 Lanuza et al. 2023 Lanuza et al. 2023 Lanuza et al. 2023 Lanuza et al. 2023 Lanuza et al. 2023 Lanuza et al. 2023 Laveriano-Santos et al. 2022 Laveriano-Santos et al. 2022 Laveriano-Santos et al. 2022 Laveriano-Santos et al. 2022 Laveriano-Santos et al. 2022 Laveriano-Santos et al. 2022 Laveriano-Santos et al. 2022 Laveriano-Santos et al. 2022 |
| **Blood Pressure/Hemodynamics** | | |
| 24h SBP | ↑ (4R)-5(3’hydroxyphenyl)-γ-valerolactone-4’-sulfate ↑ 2,3-dihydroxybenzoic acid ↑ 2,5-dihydroxybenzoic acid ↑ 2,6-dihydroxybenzene-1-sulfate ↑ 2-hydroxy-6-methoxybenzene-1-sulfate ↑ 3-hydroxy-4-methoxybenzoic acid-5-sulfate ↑ 3(4’-hydroxy-3’-methoxyphenyl)propanoic acid ↑ 4’-hydroxyhippuric acid ↑ 4-hydroxybenzoic acid ↑ 4-hydroxy-3-methoxybenzoic acid ↑ 4-hydroxy-3,5-dimethoxybenzoic acid | Le Sayec et al. 2022 Le Sayec et al. 2022 Le Sayec et al. 2022 Le Sayec et al. 2022 Le Sayec et al. 2022 Le Sayec et al. 2022 Le Sayec et al. 2022 Le Sayec et al. 2022 Le Sayec et al. 2022  Le Sayec et al. 2022 Le Sayec et al. 2022 |
| 24h DBP | ↑ 3,5-dihydroxybenzoic acid ↑ 3-(3’,5’-dihydroxyphenyl)propanoic acid ↑ 3(4’-hydroxy-3’-methoxyphenyl)propanoic acid ↑ 4-hydroxy-3-methoxybenzoic acid (vanillic acid) ↑ 3-methoxybenzoic acid-4-sulfate ↑ 3-hydroxy-4-methoxybenzoic acid-5-sulfate ↑ 3’-hydroxyphenylacetic acid ↑ 3’,4’-dihydroxyphenylacetic acid ↑ 3’-hydroxyhippuric acid ↑ 3’-hydroxycinnamic acid-4’-glucuronide ↑ 3’,4’-dihydroxycinnamic acid ↑ 4’-hydroxycinnamic acid-3’-glucuronide ↑ 4’-methoxycinnamic acid-3’-glucuronide | Le Sayec et al. 2022 Le Sayec et al. 2022 Le Sayec et al. 2022 Le Sayec et al. 2022 Le Sayec et al. 2022 Le Sayec et al. 2022 Le Sayec et al. 2022 Le Sayec et al. 2022 Le Sayec et al. 2022 |
| SBP | ↑ (4R)-5(3’hydroxyphenyl)-γ-valerolactone-4’-sulfate ↑ 2,3-Dihydroxyphenyl-1-sulfate ↑ 3-hydroxy-2-methoxybenzene-1-sulfate ↑ 3-hydroxy-2-methoxybenzoic acid-5-sulfate ↑ 4-hydroxy-3-methoxybenzaldehyde ↑ 4-hydroxy-3-methoxybenzoic acid ↑ Low Anthocyanin Metabolisers ↓ 2,6-dihydroxybenzoic acid ↓ 2-hydroxyphenylacetic acid ↓ 3-(2-hydroxyphenyl)propionic acid ↓ 3-(4’hydroxyphenyl)propanoic acid-3’glucuronide ↓ 3,4-dihydroxybenzoic acid ↓ 3’-hydroxycinnamic acid,  ↓ 3(3’-hydroxyphenyl)propanoic acid ↓ 3-methoxybenzoic acid-4-sulfate ↓ 4-hydroxy-3-methoxybenzoic acid ↓ 4ʹ-methoxycinnamic acid-3ʹ-sulfate ↓ Cinnamoylglycine ↓ Dihydroesveratrol ↓ Dihydroxyphenylpropanoic acid | Le Sayec et al. 2022 Le Sayec et al. 2022 Le Sayec et al. 2022 Le Sayec et al. 2022 Le Sayec et al. 2022 Le Sayec et al. 2022 Flynn 2021 Lanuza et al. 2023 Lanuza et al. 2023 Lanuza et al. 2023 Le Sayec et al. 2022 Lanuza et al. 2023 Le Sayec et al. 2022 Le Sayec et al. 2022 Le Sayec et al. 2022 Le Sayec et al. 2022 Lanuza et al. 2023, Le Sayec et al. 2022 Lanuza et al. 2023 Laveriano-Santos et al. 2022 Laveriano-Santos et al. 2022 |
| DBP | ↑ Low Anthocyanin Metabolisers ↓ 2,6-dihydroxybenzoic acid ↓ 3,4-dihydroxybenzoic acid ↓ Cinnamoylglycine ↓ Urolithin A ↓ Urolithin A aglycone ↓ Urolithin A glucuronide ↓ Urolithin A sulfate ↓ Urolithin B ↓ Urolithin B aglycone ↓ Urolithin B glucuronide ↓ Urolithin B sulfate | Flynn 2021 Lanuza et al. 2023 Lanuza et al. 2023 Lanuza et al. 2023 Laveriano-Santos et al. 2022 Laveriano-Santos et al. 2022 Laveriano-Santos et al. 2022 Laveriano-Santos et al. 2022 Laveriano-Santos et al. 2022 Laveriano-Santos et al. 2022 Laveriano-Santos et al. 2022 Laveriano-Santos et al. 2022 |
| BP (SBP/DBP) | ↑ 2,6-hydroxybenzoic acid ↑ 4-hydroxyhippurate ↓ (4R)5(3'-hydroxyphenyl)-y-valerolactone-4'-sulfate ↓ 4-hydroxyphenylacetic acid ↓ 4-hydroxyphenylpropionic acid | Grohmann et al. 2023 Zheng et al. 2013 Le Sayec et al. 2022 Grohmann et al. 2023 Grohmann et al. 2023 |
| **Glycemic Outcomes** | |  |
| Blood glucose | ↓ Isoferulic-3′-O-glucuronide ↓ Ferulic-3′-O-glucuronide | Coelho et al. 2021 Coelho et al. 2021 |
| HbA1c | ↓ 2,6-dihydroxybenzoic acid ↓ Cinnamoylglycine | Lanuza et al. 2023 Lanuza et al. 2023 |
| PreDM-IR Cohort vs. Healthy Control | ↓ 3,8-dihydroxy-urolithin (urolithin A) derivatives ↓ Benzoic acids ↓ Dihydroxycinnamic acids ↓ Hippuric acid  ↓ Hydroxyhippuric acids derivatives ↓ Phenyl-γ-valerolactones | Zhang et al. 2020 Zhang et al. 2020 Zhang et al. 2020 Zhang et al. 2020 Zhang et al. 2020 Zhang et al. 2020 |
| **Lipids/Lipoproteins** | |  |
| TG | ↑ 3-hydroxyphenylacetic acid ↓ 2,6-dihydroxybenzoic acid ↓ 2-hydroxyphenylacetic acid ↓ 3-(2-hydroxyphenyl)propionic acid ↓ 3,4-dihydroxybenzoic acid ↓ Cinnamoylglycine ↑ Coumaric acids ↓ Gallic acid ↓ Gallic acid aglycone ↓ Gallic acid glucuronide ↓ Gallic acid sulfate | Lanuza et al. 2023 Lanuza et al. 2023 Lanuza et al. 2023 Lanuza et al. 2023 Lanuza et al. 2023 Lanuza et al. 2023 Laveriano-Santos et al. 2022 Laveriano-Santos et al. 2022 Laveriano-Santos et al. 2022 Laveriano-Santos et al. 2022 Laveriano-Santos et al. 2022 |
| HDLc | ↑ 2-hydroxyphenylacetic acid ↑ 3-(2-hydroxyphenyl)propionic acid ↑ Urolithin A glucuronide ↑ Urolithin A sulfate ↓ 3-hydroxyphenylacetic acid | Lanuza et al. 2023 Lanuza et al. 2023 Lanuza et al. 2023 Lanuza et al. 2023 Lanuza et al. 2023 |
| small-HDLc | ↓ Urolithin A | Cortés-Martín et al. 2024 |
| non-HDLc | ↓ Urolithin A (in UM-B individuals only) ↑ UM-B vs. UM-A (at baseline)  ↓ UM-B only (post-intervention) | González-Sarrías et al. 2017 González-Sarrías et al. 2017  González-Sarrías et al. 2017 |
| LDLc | ↓ Isourolithin A + Urolithin B (in UM-B individuals only) ↓ Urolithin A (in UM-B individuals only)  ↓ Total urolithins (in UM-B individuals only) ↓ 2,6-dihydroxybenzoic acid ↓ 3,4-dihydroxybenzoic acid ↓ Cinnamoylglycine ↑ UM-B vs. UM-A (at baseline)  ↓ UM-B only (post-intervention) | Gonzalez-Sarrias et al. 2017 Gonzalez-Sarrias et al. 2017 Gonzalez-Sarrias et al. 2017 Lanuza et al. 2023 Lanuza et al. 2023 Lanuza et al. 2023 Gonzalez-Sarrias et al. 2017  González-Sarrías et al. 2017 |
| small-LDLc | ↑ UM-B vs. UM-A (at baseline)  ↓ UM-B only (post-intervention) | González-Sarrías et al. 2017  González-Sarrías et al. 2017 |
| oxLDLc | ↑ UM-B vs. UM-A (at baseline)  ↓ UM-B only (post-intervention) | González-Sarrías et al. 2017  González-Sarrías et al. 2017 |
| ApoA | ↓ Urolithin A | Cortés-Martín et al. 2024 |
| VLDLc | ↑ UM-B vs. UM-A (at baseline) | González-Sarrías et al. 2017 |
| IDLc | ↑ UM-B vs. UM-A (at baseline) | González-Sarrías et al. 2017 |
| LDLc/HDLc | ↑ UM-B vs. UM-A (at baseline) | González-Sarrías et al. 2017 |
| ApoB | ↑ UM-B vs. UM-A (at baseline)  ↓ UM-B only (post-intervention) | González-Sarrías et al. 2017  González-Sarrías et al. 2017 |
| ApoA-1 | ↓ Urolithin A | Cortés-Martín et al. 2024 |
| Total Cholesterol | ↓ Low Anthocyanin Metabolizers  ↓ Urolithin A (in UM-B individuals only)  ↓ Total urolithins (in UM-B individuals only)  ↑ UM-B vs. UM-A (at baseline)  ↓ UM-B only (post-intervention) | Flynn 2021  González-Sarrías et al. 2017  González-Sarrías et al. 2017  González-Sarrías et al. 2017  González-Sarrías et al. 2017 |
| **Disease Risk, Aggregate Risk Scores, and other Composite Measures** | | |
| T2DM Risk | ↑ m-coumaric acid ↓ 4-hydroxybenzoic acid ↓ Hydroxybenzoic acid glucuronide ↓ Hydroxytyrosol sulphate ↓ Vanillic acid sulphate | Marhuenda-Muñoz et al. 2022 Marhuenda-Muñoz et al. 2022 Marhuenda-Muñoz et al. 2022 Marhuenda-Muñoz et al. 2022 Marhuenda-Muñoz et al. 2022 |
| MetS score | ↓ Gallic acid ↓ Gallic acid aglycone ↓ Gallic acid glucuronide ↓ Gallic acid sulfate ↓ Urolithin B ↓ Urolithin B aglycone ↓ Urolithin B glucuronide ↓ Urolithin B sulfate | Laveriano-Santos et al. 2022 Laveriano-Santos et al. 2022 Laveriano-Santos et al. 2022 Laveriano-Santos et al. 2022 Laveriano-Santos et al. 2022 Laveriano-Santos et al. 2022 Laveriano-Santos et al. 2022 Laveriano-Santos et al. 2022 |
| **Null Findings or Planned Assessments** | |  |
| Null Findings | × Anthocyanins ⊥ glucose × Urolithins (individual metabolites) ⊥ ApoB  × Urolithin Metabotypes ⊥ HOMA-IR, glucose, insulin, TG, IDLc, ApoA, HDLc | Flynn 2021 González-Sarrías et al. 2017  González-Sarrías et al. 2017 |
| Planned assessment | (poly)phenols ↔ Anthropomorphic measures (poly)phenols ↔ Adipose and body composition (poly)phenols ↔ Biomarkers of Cardiometabolic health (poly)phenols ↔ BP (poly)phenols ↔ CVD risk (poly)phenols ↔ Genetic differences (poly)phenols ↔ Heart rate (poly)phenols ↔ Inflammatory biomarkers (poly)phenols ↔ Risk prediction scores | NCT06347094 NCT06347094 NCT06347094 NCT06347094 NCT06347094 NCT06347094 NCT06347094 NCT06347094 NCT06347094 |
| **Key:**  Outcome abbreviations: (FMD) Flow-mediated dilation, (D₀) Pre-occlusion vessel diameter, (PWV) Pulse wave velocity, (AIX) Aortic augmentation index, (VAT) Visceral adipose tissue (visceral fat mass), (WC) Waist circumference, (BP) Blood pressure, (SBP) Systolic blood pressure, (DBP) Diastolic blood pressure, (MAP) Mean arterial pressure, (BG) Blood glucose, (TG) Triglycerides, (HDL-c) High-density lipoprotein cholesterol, (XL-HDL-P) Extra-large high-density lipoprotein particles, (LDL-c) Low-density lipoprotein cholesterol, (HbA1c) Hemoglobin A1c, (PreDM-IR) Prediabetes with insulin resistance, (T2DM) Type 2 diabetes mellitus, (ICVH) Ideal cardiovascular health, (ASCVD) Atherosclerotic cardiovascular disease, (MetS) Metabolic syndrome (score)  Low Anthocyanin Metabolizers: Measured by metabolism of hippuric acid, 3-hydroxyhippuric acid, 4-hydroxy-3-methoxyphenylacetic acid, and 3,5dihydroxyphenylpropionic acid  ↑ Shows positive correlation ↓ Shows inverse correlation × Shows no correlation with ⊥ showing statistical independence ↔ Relationship to be investigated: upcoming or ongoing trial | | |

Supplementary Table S1C. Relationships between cardiometabolic outcomes and MPMs – Existing Conditions

|  | **Existing Condition** | |
| --- | --- | --- |
| **Outcome** | **Result** | **Reference** |
| **Adiposity/Body Composition** | | |
| Change in Body fat (%) | ↑ Change in Urolithin A glucuronide (post-intervention) | Mora-Cubillos et al. 2015 |
| WC | ↓ Urolithin A glucuronide | Mora-Cubillos et al. 2015 |
| Wait-to-hip ratio | ↓ Urolithin A glucuronide | Mora-Cubillos et al. 2015 |
| BMI | ↑ UM-B vs. UM-A | Cortés-Martín et al. 2018b |
| **Blood Pressure/Hemodynamics** | | |
| DBP | ↑ Urolithin B-glucuronide | Domínguez-López et al. 2023 |
| **Glycemic Control** | | |
| Fasting Insulin | ↓ Urolithin A glucuronide | Mora-Cubillos et al. 2013 |
| HOMA-IR | ↓ Urolithin A glucuronide | Mora-Cubillos et al. 2014 |
| **Lipids/Lipoproteins** | | |
| TG | ↓ Resveratrol microbe-host co-metabolites | Bullón-Vela et al. 2020 |
| HDLc | ↑ 3-hydroxyphenylacetic ↑ Benzoylglutamic acid ↑ Vanillic acids | Khana et al. 2012 Curtis et al. 2022 Khana et al. 2012 |
| XL-HDLP | ↑ Hippuric acid  ↑ Hippuric acid-sulfate | Curtis et al. 2022 Curtis et al. 2022 |
| LDLc | ↓ Urolithin B-glucuronide | Domínguez-López et al. 2023 |
| oxLDLc | ↓ 3-hydroxyphenylacetic ↓ Vanillic acids | Khana et al. 2012 Khana et al. 2012 |
| Apo-A1 | ↑ 3-methoxyphenylacetic acid-4-sulfate | Curtis et al. 2022 |
| Cholesterol | ↓ Benzoylglutamic acid ↓ Benzoic acid-4-sulfate | Curtis et al. 2022 Curtis et al. 2022 |
| **Disease Risk, Aggregate Risk Scores, and other Composite Measures** | | |
| ICVH | ↑ Microbial polyphenol metabolite score | Domínguez-López et al. 2023 |
| **Null Findings** | | |
| Null Findings | × Hesperetin ⊥ ACS × Microbial polyphenol metabolite score ⊥ HDLc × Microbial polyphenol metabolite score ⊥ SBP × Naringenin ⊥ ACS × resveratrol microbe-host co-metabolites ⊥ glucose metabolism markers  × resveratrol microbe-host co-metabolites ⊥ LDL-c, triglyceride/HDL ratio  × Tamarixetin ⊥ ACS × UM ⊥ CVD risk factors*  × UM ⊥ TG, TC, LDLc, blood pressure, insulin, glucose, HOMA-IR, weight × Urolithin A glucuronide ⊥ blood lipids × Urolithin A glucuronide ⊥ blood pressure  × Urolithin A glucuronide ⊥ BMI  × Urolithin A glucuronide ⊥ Fasting glucose × Vanillic acid glucuronide ⊥ TG | Rienks et al. 2017 Domínguez-López et al. 2023 Domínguez-López et al. 2023 Rienks et al. 2017 Bullón-Vela et al. 2020 Bullón-Vela et al. 2020 Rienks et al. 2017 Cortés-Martín et al. 2021  Meroño et al. 2022 Mora-Cubillos et al. 2015 Mora-Cubillos et al. 2015  Mora-Cubillos et al. 2015  Mora-Cubillos et al. 2015 Domínguez-López et al. 2023 |
| **Key:**  Outcome abbreviations: (FMD) Flow-mediated dilation, (D₀) Pre-occlusion vessel diameter, (PWV) Pulse wave velocity, (AIX) Aortic augmentation index, (VAT) Visceral adipose tissue (visceral fat mass), (WC) Waist circumference, (BP) Blood pressure, (SBP) Systolic blood pressure, (DBP) Diastolic blood pressure, (MAP) Mean arterial pressure, (BG) Blood glucose, (TG) Triglycerides, (HDL-c) High-density lipoprotein cholesterol, (XL-HDL-P) Extra-large high-density lipoprotein particles, (LDL-c) Low-density lipoprotein cholesterol, (HbA1c) Hemoglobin A1c, (PreDM-IR) Prediabetes with insulin resistance, (T2DM) Type 2 diabetes mellitus, (ICVH) Ideal cardiovascular health, (ASCVD) Atherosclerotic cardiovascular disease, (ACS) Acute coronary syndrome  Glucose metabolism markers- glucose, HbA1c, insulin, HOMA-IR, HOMA-%B, FGIR, FIRI  Low Anthocyanin Metabolizers: Measured by metabolism of hippuric acid, 3-hydroxyhippuric acid, 4-hydroxy-3-methoxyphenylacetic acid, and 3,5dihydroxyphenylpropionic acid  *study dos not specify  ↑ Shows positive correlation ↓ Shows inverse correlation × Shows no correlation with ⊥ showing statistical independence ↔ Relationship to be investigated: upcoming or ongoing trial | | |

Supplementary Table S1D. Relationships between immunological or oxidative outcomes and MPMs

| **Immune or Oxidative-related Outcome** | **Result** | **Reference** |  |
| --- | --- | --- | --- |
| **Endotoxin** | | |  |
| Endotoxin | ↓ Sum of Gallotannin metabolites¹ | Barnes et al. 2019 |  |
| **Cytokines** | | |  |
| IL-8 | ↑ Phthalic acid ↑ Phenylacetic acid ↑ Total Phenolic content | Gutiérrez-Díaz et al. 2018 Gutiérrez-Díaz et al. 2018 Gutiérrez-Díaz et al. 2018 |  |
| IL-17 | ↑ Phenylacetic acid ↑ Total Phenolic content | Gutiérrez-Díaz et al. 2018 Gutiérrez-Díaz et al. 2018 |  |
| IL-10 | ↑ Galloyl metabolites ↑ Phenylacetic acid | Kim et al. 2017 Gutiérrez-Díaz et al. 2018 |  |
| TGF-β | ↑ Phenylacetic acid ↑ Phthalic acid ↑ Protocatechuic acid ↑ Total Phenolic content | Gutiérrez-Díaz et al. 2018 Gutiérrez-Díaz et al. 2018 Gutiérrez-Díaz et al. 2018 Gutiérrez-Díaz et al. 2018 |  |
| **Acute Phase/General Inflammatory Markers** | | |  |
| CRP | ↑ Phenylacetic acid | Gutiérrez-Díaz et al. 2018 |  |
| hsCRP | ↓ 2,6-dihydroxybenzoic acid ↓ 3,4-dihydroxybenzoic acid ↓ 3,4-dihydroxyphenylpropionic acid ↓ 3,5-dihydroxybenzoic acid ↓ Caffeic acid ↓ Cinnamoylglycine ↓ Ferulic acid ↓ Hydroxytyrosol | Lanuza et al. 2023 Lanuza et al. 2023 Harms et al. 2020 Harms et al. 2020 Harms et al. 2020 Lanuza et al. 2023 Harms et al. 2020 Harms et al. 2020 |  |
| AISI | ↓ Naringenin-7-O-β-D-glucuronide | Bullón-Vela et al., 2023 |  |
| SII | ↓ Naringenin-7-O-β-D-glucuronide | Bullón-Vela et al., 2023 |  |
| PAI-1 | ↓ Dihydroferulic acid ↓ Ferulic acid | Vitaglione et al. 2015 Vitaglione et al. 2015 |  |
| **Oxidative Stress** | | |  |
| GGT | ↓ Hesperetin-3′-O-β-D-glucuronide ↓ Naringenin-4′-O-β-D-glucuronide ↓ Naringenin-7-O-β-D-glucuronide | Bullón-Vela et al., 2023 Bullón-Vela et al., 2023 Bullón-Vela et al., 2023 |  |
| MDA | ↑ Phenylacetic acid | Gutiérrez-Díaz et al. 2018 |  |
| **Pemphigus Specific study** | | |  |
| Pemphigus vs. Control | ↓ 2-hydroxy-3-(4-hydroxyphenyl)propanoic acid ↓ Cinnamic acid ↓ Pinocembrin  ↓ Vanillin acetate | Guo et al., 2023 Guo et al., 2023 Guo et al., 2023 Guo et al., 2023 |  |
| **Null Findings** | | |  |
| Null Findings | × Urolithin Metabotype ⊥ IL-6 × Urolithin Metabotype ⊥ TNF-α × Urolithin Metabotypes ⊥ CRP × Urolithin Metabotypes ⊥ LBP × Urolithin Metabotypes ⊥ LBP × Urolithin Metabotypes ⊥ sICAM-1 × Urolithins ⊥ CRP × Urolithins ⊥ ICAM-1 × Urolithins ⊥ IL-6 × Urolithins ⊥ LBP × Urolithins ⊥ TNF-α × Urolithins ⊥ VCAM-1 × Urolithins ⊥TEAC  ×Urolithins ⊥ Urinary 8-iso-PGF2 | Meroño et al. 2022 Meroño et al. 2022 Meroño et al. 2022 González‐Sarrías et al. 2018 Cortés-Martín et al. 2021 Meroño et al. 2022 Meroño et al. 2022 Meroño et al. 2022 Meroño et al. 2022 González‐Sarrías et al. 2018 Meroño et al. 2022 Meroño et al. 2022 Cerdá et al. 2006 Cerdá et al. 2006 |  |
| Planned assessments (from protocols) | phenyl-γ-valerolactones ↔ CRP phenyl-γ-valerolactones ↔ IL-6 phenyl-γ-valerolactones ↔ IL-10 phenyl-γ-valerolactones ↔ TNF-α Xanthohumol ↔ IL-1  Xanthohumol ↔ IL-10  Xanthohumol ↔ IL-12  Xanthohumol ↔ IL-17 Xanthohumol ↔ LBP Xanthohumol ↔ LPS Xanthohumol ↔ Platelet function Xanthohumol ↔ TNF-α | Angelino et al. 2020 Angelino et al. 2020 Angelino et al. 2020 Angelino et al. 2020 Langley et al. 2022 Langley et al. 2022 Langley et al. 2022 Langley et al. 2022 Langley et al. 2022 Langley et al. 2022 Langley et al. 2022 Langley et al. 2022 |  |
| **Key:**  Outcomes Abbreviations: (IL-#) Interleukin-#, (TNF-α) tumor necrosis factor-α, (TGF-β) Transforming growth factor-β, (CRP) c-reactive protein, (ICAM-I) Intercellular adhesion molecule-1, (GGT), Gamma-Glutamyl Transferase, (AISI) Aggregate index of systemic inflammation, (SII)Systemic inflammation index, (PAI-1) Plasminogen Activator Inhibitor-1, (15-F2t-IsoP-M) 2,3-dinor-5,6-dihydro-15-F2t-isoprostane, (F2-IsoPs) F2-isoprostanes, (MDA) Malondialdehyde, (LBP) Lipopolysaccharide binding protein*  *Used as a measure of inflammation ¹Gallic acid, 4-O-methylgallic acid, 4-O-methylgallic acid-3-O-sulfate, Pyrogallol, Pyrogallol-O-sulfate, Methylpryogallol-O-sulfate, Catechol-O-sulfate ↑ Shows positive correlation ↓ Shows inverse correlation × Shows no correlation with ⊥ showing statistical independence ↔ ↔ Relationship to be investigated: upcoming or ongoing trial | | |  |
|  |  |  |  |

Supplementary Table S1E. Relationships between neurological outcomes and MPMs

| **Neurological-Related Outcome** | **Result** | **Reference** |
| --- | --- | --- |
| **Attention** | | |
| Attention | ↑ 3,4-dihydroxyphenylacetic acid ↑ 3-hydroxybenzoic acid ↑ Benzoic acid-4-sulfate ↑ Hydroxymethoxybenzoic acid-sulfate ↑ Trans-3-hydroxycinnamic acid (m-Coumaric acid) | Curtis et al., 2024 Curtis et al., 2024 Curtis et al., 2024 Curtis et al., 2024 Curtis et al., 2024 |
| Alertness | ↑ 4-hydroxy-3,5-dimethoxyphenylacetic acid (homosyringic acid) | Curtis et al., 2024 |
| **Mood** | | |
| Anxiety score | ↓ 3-hydroxybenzoic acid ↓ Hydroxybenzoic acid sulfate ↓ Protocatechuic acid sulfate ↓ Syringic acid sulfate | Parilli-Moser et al., 2023 Parilli-Moser et al., 2023 Parilli-Moser et al., 2023 Parilli-Moser et al., 2023 |
| Depression score | ↓ 3-hydroxybenzoic acid ↓ 4-hydroxybenzoic acid ↓ Protocatechuic acid sulfate ↓ Syringic acid glucuronide I ↓ Syringic acid sulfate | Parilli-Moser et al., 2023 Parilli-Moser et al., 2023 Parilli-Moser et al., 2023 Parilli-Moser et al., 2023 Parilli-Moser et al., 2023 |
| Calmness | ↑ 3-hydroxy-4-methoxycinnamic acid | Curtis et al., 2024 |
| **Episodic memory** | | |
| Episodic memory | ↑ 3-methoxyphenylacetic acid-4-sulfate ↑ 3-hydroxyhippuric acid ↑ Hippuric acid | Curtis et al., 2024 Curtis et al., 2024 Flanagan, 2021 |
| Total memory score | ↑ 3-hydroxybenzoic acid  ↑ 4-hydroxybenzoic acid  ↑ hydroxybenzoic acid sulfate  ↑ Syringic acid sulfate  ↑ Total hydroxybenzoic acids  ↑ Vanillic acid sulfate | Parilli-Moser et al., 2023 Parilli-Moser et al., 2023 Parilli-Moser et al., 2023 Parilli-Moser et al., 2023 Parilli-Moser et al., 2023 Parilli-Moser et al., 2023 |
| Verbal episodic memory | - | - |
| Immediate verbal recall | ↑ 2,5-dihydroxybenzoic acid ↑ 2,6- dihydroxybenzoic acid ↑ 2,6-dihydroxybenzene-1-sulfate ↑ 2-hydroxy-3-methoxybenzene-1-sulfate  ↑ 3-(2′,3′-dihydroxyphenyl)propanoic acid (dihydrocaffeic acid) ↑ Benzoic acid ↑ Phenylacetic acid ↓ Isoferulic acid | Wood et al., 2023 Wood et al., 2023 Wood et al., 2023 Wood et al., 2023 Wood et al., 2023 Wood et al., 2023 Wood et al., 2023 Wood et al., 2023 |
| Delayed verbal recall | ↑ 2-hydroxyhippuric acid. ↑ 3-(4′-methoxyphenyl)propanoic acid-3′-sulfate  ↑ 3′-hydroxy-4′-methoxyphenyl)propanoic acid-3′-glucuronide (dihydroisoferulic acid 3-glucuronide) | Wood et al., 2023 Wood et al., 2023 Wood et al., 2023 |
| Total verbal memory | ↑ 3-hydroxybenzoic acid  ↑ Hydroxybenzoic acid sulfate  ↑ Protocatechuic acid sulfate  ↑ Syringic acid sulfate  ↑ Total hydroxybenzoic acids  ↑ Vanillic acid sulfate | Parilli-Moser et al., 2023 Parilli-Moser et al., 2023 Parilli-Moser et al., 2023 Parilli-Moser et al., 2023 Parilli-Moser et al., 2023 Parilli-Moser et al., 2023 |
| Visual episodic memory | - | - |
| Visual recognition memory | ↑ 3-hydroxyhippuric acid ↑ Hippuric acid | Curtis et al., 2024 Curtis et al., 2024 |
| **Executive function** | | |
| Executive function | ↑ 4-hydroxybenzoic acid ↑ Benzoylglutamic acid ↓ 2,4-dihydroxybenzoic acid ↓ 2,6-dihydroxybenzoic acid | Curtis et al., 2024 Curtis et al., 2024 Wood et al., 2023 Wood et al., 2023 |
| Cognitive flexibility score (lower score = better task switching) | ↓ 3-hydroxybenzoic acid  ↓ 4-hydroxybenzoic acid  ↓ Hydroxybenzoic acid sulfate  ↓ Protocatechuic acid sulfate  ↓ Syringic acid sulfate  ↓ Total hydroxybenzoic acids  ↓ Vanillic acid sulfate | Parilli-Moser et al., 2023 Parilli-Moser et al., 2023 Parilli-Moser et al., 2023 Parilli-Moser et al., 2023 Parilli-Moser et al., 2023 Parilli-Moser et al., 2023 Parilli-Moser et al., 2023 |
| **Cognition** | | |
| Cognitive function (as measured via MMSE scores) | ↑ Hydroxyphenylacetic acid ↑ Protochatechuic acid ↑ Urolithin A ↑ Urolithin A sulfate ↑ Urolithin A sulphoglucuronide ↑ Urolithin B ↑ Urolithin B glucuronide | Rabassa et al., 2020 Domínguez-López et al., 2024 Rabassa et al., 2020 Rabassa et al., 2020 Rabassa et al., 2020 Rabassa et al., 2020 Rabassa et al., 2020 |
| Odds of cognitive decline | ↑ 4-hydroxyphenylacetic acid-glucuronide ↓ 3-hydroxybenzoic acid sulfate ↓ 3-hydroxyphenylacetic acid-sulfate ↓ Hydroxyphenylacetic acid ↓ Urolithin A ↓ Urolithin A sulfate ↓ Urolithin B ↓ Urolithin B glucuronide | González-Domínguez et al., 2020 González-Domínguez et al., 2020 González-Domínguez et al., 2020 Rabassa et al., 2020 Rabassa et al., 2020 Rabassa et al., 2020 Rabassa et al., 2020 Rabassa et al., 2020 |
| Global Cognition | ↑ 3-hydroxybenzoic acid ↑ Protochatechuic acid ↑ Vanillic acid glucuronide | Domínguez-López et al., 2024 Domínguez-López et al., 2024 Domínguez-López et al., 2024 |
| **Working memory** | | |
| Working memory | ↑ 3-hydroxybenzoic acid ↑ 3-methoxybenzoic acid-4-sulfate ↑ 4-hydroxybenzoic acid ↑ 4-methoxybenzoic acid-3-sulfate ↑ Hydroxybenzoic acid sulfate ↑ Protocatechuic acid glucuronide II ↑ Total hydroxybenzoic acids ↑ Vanillic acid sulfate | Parilli-Moser et al., 2023 Curtis et al., 2024 Parilli-Moser et al., 2023 Curtis et al., 2024 Parilli-Moser et al., 2023 Parilli-Moser et al., 2023 Parilli-Moser et al., 2023 Parilli-Moser et al., 2023 |
| Immediate memory | ↑ 3,4-dihydroxyphenylacetic acid ↑ Hippuric acid | Curtis et al., 2024 Curtis et al., 2024 |
| **Motor/Disease status** | | |
| Parkinson's duration | ↑ UM-0 vs. UM-A & UM-B ↓ UM-A vs. UM-0 ↓ UM-B vs. UM-0 | Romo-Vaquero et al., 2022 Romo-Vaquero et al., 2022 Romo-Vaquero et al., 2022 |
| Parkinson's stage | ↑ UM-0 vs. UM-A & UM-B | Romo-Vaquero et al., 2022 |
| **Pain** | | |
| Post-operative pain score | ↓ UM-A vs. UM-0 ↓ Urolithin A ↓ UM-B vs. UM-0 | Volpp et al., 2020 Volpp et al., 2020 Volpp et al., 2020 |
| **Sleep and Arousal** | | |
| Wakefulness | ↑ 3-(4-hydroxy-3-methoxyphenyl)propionic acid (dihydroferulic acid) ↑ 4-hydroxy-3,5-dimethoxyphenylacetic acid | Curtis et al., 2024 Curtis et al., 2024 |
| Quality of sleep | ↑ 3-Hydroxy-4-methoxyphenylacetic acid (homovanillic acid) ↑ 3-Methoxyphenylacetic acid 4-sulfate | Curtis et al., 2024 Curtis et al., 2024 |
| **Null Findings** | | |
| Null Findings | × y-valerolactones ⊥ VRM immediate free recall total correct, PP × y-valerolactones ⊥ VRM immediate free recall total correct, Decliners × y-valerolactones ⊥ VRM-delayed recognition total correct, Decliners | Bensalem et al., 2018 Bensalem et al., 2018 Bensalem et al., 2018 |
| Planned assessment | PVLs ↔ cognitive function  PVLs ↔ health outcomes in ageing | Angelino et al., 2020 Angelino et al., 2020 |
| **Key:**  Outcome Abbreviations: (VRM) Visual Reproduction Memory, (UM-A) Urolithin metabotype A, (UM-B) Urolithin metabotype B, (UM-0) Urolithin metabotype 0 (non-producer), (PVLs) Phenyl-γ-valerolactones, (MMSE) Mini-Mental State Examination  ↑ Shows positive correlation ↓ Shows inverse correlation × Shows no correlation with ⊥ showing statistical independence ↔ Relationship to be investigated: upcoming or ongoing trial | | |

Supplementary Table S1F. Relationships between gastrointestinal or digestive health outcomes and MPMs

| **GI and Digestive-related Outcome** | **Result** | **Reference** |
| --- | --- | --- |
| **Appetite & Satiety** | | |
| Thirst | ↓ Caffeic acid-O-sulfate | Coelho et al., 2021 |
| Fullness | ↓ 3-hydroxyhippuric acid | Coelho et al., 2021 |
| Desire to eat | ↓ 4-OH-benzaldehyde | Coelho et al., 2021 |
| **Intestinal Permeability** | | |
| Zonulin | ↑ 2-hydroxybenzoic acid glucuronide ↑ 3-hydroxyphenylacetic acid ↑ 5-(3',4',5'-trihydroxyphenyl)-γ-Valerolactone 3-sulfate ↑ 5-(4'-hydroxy-3'-methoxyphenyl)-γ-valerolactone glucuronide ↑ Catechol sulfate ↑ Hippuric acid ↑ Isovanillic acid glucuronide ↑ Vanillic acid glucuronide ↓ 3,4-dihydroxybenzoic acid ↓ 3,4-dihydroxybenzoic acid 3-glucuronide ↓ 3,4-dihydroxyphenylacetic acid glucuronide ↓ 3-hydroxybenzoic acid sulfate ↓ 4-methylgallic acid ↓ 5-(3',4'-dihydroxyphenyl)-4-hydroxyvaleric acid 3'-glucurnoide ↓ 5-(3',4'-dihydroxyphenyl)-4-hydroxyvaleric acid 3'-sulfate ↓ 5-(3',4'-dihydroxyphenyl)-γ-valerolactone 3'-glucuronide ↓ 5-(3',4'-dihydroxyphenyl)-γ-valerolactone 3'-sulfate ↓ 5-(3',4'-dihydroxyphenyl)-γ-valerolactone 4'-glucuronide ↓ 5-(3',4'-dihydroxyphenyl)-γ-valerolactone 4'-sulfate ↓ 5-(4'-hydroxy-3'-methoxyphenyl)-γ-valerolactone ↓ 5-(4'-hydroxy-3'-methoxyphenyl)-γ-valerolactone sulfate ↓ Caffeic acid 3-glucuronide ↓ m-coumaric acid glucuronide ↓ m-Coumaric acid glucuronide ↓ m-coumaric acid sulfate ↓ Methylgallic acid glucuronide ↓ Methylpyrogallol sulfate ↓ Naringenin glucuronide ↓ o-Coumaric acid ↓ o-Coumaric acid glucuronide ↓ p-Coumaric acid glucuronide ↓ Syringic acid ↓ Urolithin metabotype B vs. Urolithin metabotype A ↓ Urolithin A glucuronide ↓ Urolithin A sulfate ↓3,4-dihydroxybenzoic acid 4-glucuronide ↓3-hydroxybenzoic acid glucuronide | Hidalgo-Liberona et al., 2020 Hidalgo-Liberona et al., 2020 Hidalgo-Liberona et al., 2020 Hidalgo-Liberona et al., 2020 Peron et al., 2021 Peron et al., 2021 Hidalgo-Liberona et al., 2020 Hidalgo-Liberona et al., 2020 Hidalgo-Liberona et al., 2020 Hidalgo-Liberona et al., 2020 Hidalgo-Liberona et al., 2020 Hidalgo-Liberona et al., 2020 Hidalgo-Liberona et al., 2020 Hidalgo-Liberona et al., 2020 Hidalgo-Liberona et al., 2020 Hidalgo-Liberona et al., 2020 Hidalgo-Liberona et al., 2020 Hidalgo-Liberona et al., 2020 Hidalgo-Liberona et al., 2020 Hidalgo-Liberona et al., 2020 Hidalgo-Liberona et al., 2020 Hidalgo-Liberona et al., 2020 Hidalgo-Liberona et al., 2020 Hidalgo-Liberona et al., 2020 Hidalgo-Liberona et al., 2020 Hidalgo-Liberona et al., 2020 Hidalgo-Liberona et al., 2020 Hidalgo-Liberona et al., 2020 Hidalgo-Liberona et al., 2020 Hidalgo-Liberona et al., 2020 Hidalgo-Liberona et al., 2020 Hidalgo-Liberona et al., 2020 Meroño et al., 2022 Hidalgo-Liberona et al., 2020 Hidalgo-Liberona et al., 2020 Hidalgo-Liberona et al., 2020 Hidalgo-Liberona et al., 2020 |
| IFABP | ↓ 2-(4-hydroxyphenyl)propionate | Karl et al. 2022 |
| Claudin-3 | ↓ 2-(4-hydroxyphenyl)propionate | Karl et al. 2022 |
| LBP* | ↓ 2-(4-hydroxyphenyl)propionate | Karl et al. 2022 |
| **Gut Hormones** | | |
| GLP-2 | ↓ 2-(4-hydroxyphenyl)propionate | Karl et al. 2022 |
| **Null Findings** | | |
| Null Findings | × Intestinal permeability ⊥ Metabotypes × Serum zonulin ⊥ MPMs × Zonulin ⊥ Food-derived metabolites in HSZ group | Nishioka et al., 2021  Peron et al., 2021 Hidalgo-Liberona et al., 2020 |
| Relationship to be explored | CD14 ↔ Endotoxemia Intestinal fatty acid binding protein↔ Endotoxemia | Langley et al., 2022  Langley et al., 2022 |
| **Key:**  Outcome Abbreviations: (IFABP) intestinal fatty acid binding protein, (LBP) lipopolysaccharide binding protein, (GLP-2) Glucagon-like peptide-2  ↑ Shows positive correlation  ↓ Shows inverse correlation × Shows no correlation with ⊥ showing statistical independence ↔ Relationship to be investigated: upcoming or ongoing trial *LBP used as a measure for intestinal permeability | | |

Supplementary Table S1G. Relationships between cancer outcomes and MPMs

| **Cancer-Related Outcome** | **Result** | **Reference** |  |
| --- | --- | --- | --- |
| **Tissue Genetics** | | |  |
| Amino acid metabolism* | ↑ 3-[3-(sulfooxy) phenyl] propanoic acid  ↑ 3-hydroxyphenylacetate ↑ 4-methylcatechol sulfate ↑ Catechol sulfate ↑ Hippurate | Pan et al., 2015 Pan et al., 2015 Pan et al., 2015 Pan et al., 2015 |  |
| **Occurrence in Cases / Controls** | | |  |
| Controls vs. Breast cancer cases | ↑ 5-(3′,4′,5′-trihydroxyphenyl)-γ-valerolactone | Luo et al., 2009 |  |
| **Biomarkers** | | |  |
| H pylori antibodies** | ↓ 5-(3′,4′,5′-trihydroxyphenyl)-γ-valerolactone  ↓ 5-(3′,4′-dihydroxyphenyl)-γ-valerolactone | Sun et al., 2002 Sun et al., 2002 |  |
| Apoptotic marker (TUNEL) | ↑ 4-methylcatechol sulfate | Pan et al., 2015 |  |
| **Null Findings** | | |  |
| Null Findings | × Gastric/esophageal cancer risk ⊥ 5-(3',4'-dihydroxyphenyl)-γ-valerolactone × Gastric/esophageal cancer risk ⊥ 5-(3′,4′,5′-trihydroxyphenyl)-γ-valerolactone  × Gene expression (CDKN1A, MKi-67, c-Myc)*** ⊥ Urolithins × Histological type (glandular, parenchymal or stromal) ⊥ Urolithins  × Pathology (Prostate cancer or benign prostatic hyperplasia) ⊥ Urolithins | Sun et al., 2002 Sun et al., 2002 González-Sarrías et al., 2010 González-Sarrías et al., 2010 González-Sarrías et al., 2010 |  |
| **Key:**  ↑ shows a positive correlation ↓ shows an inverse correlation × Shows no correlation with ⊥ showing statistical independence ↔ Relationship to be investigated: upcoming or ongoing trial *Results from colorectal cancer patients **Helicobacter pylori infection is the strongest known gastric cancer risk, antibody testing is used as a risk factor for gastric cancer testing  *** CDKN1A, MKi-67, c-Myc are cancer-related genes | | |  |
|  |  |  |  |

Supplementary Table S1H. Relationships between epigenetic, musculoskeletal, or respiratory outcomes and MPMs

| **Epigenetics-Related Outcome** | **Result** | **Reference** |
| --- | --- | --- |
| **Epigenetic** | |  |
| Li mAge | ↓ Tyrosol | Yaskolka Meir et al., 2023 |
| **Musculoskeletal - Bone Health** |  |  |
| Bone Mineral Density | ↓ 3-phenylpropanoic acid ↓ Hippuric acid | Greenbaum et al. 2022 Greenbaum et al. 2022 |
| **Null Findings** | No relationship between MPMs and respiratory or clinical outcomes in COPD | Cerdá et al. 2006 |
| **Key:**  Li mAge: Li DNA methylation age (epigenetic clock trained to predict chronological age); MPMs (microbial-derived polyphenol metabolites); COPS (chronic obstructive pulmonary disease)  ↓ Shows inverse correlation | | |
|  |  |  |
