# Supplementary material for: Clinical evidence for microbial-derived polyphenol metabolites in health and disease: a scoping review
Source: Front Nutr. 2026 Jun 17;13:1859472. doi: 10.3389/fnut.2026.1859472 (PMC13319019; doi:10.3389/fnut.2026.1859472)
Supplement: Supplementary file 3 [file Table_2.DOCX]

**Table of Contents for Supplementary Tables S2A-S2H**

Supplementary Table S2A Summary of studies assessing cardiometabolic outcomes – Healthy Populations………………………………………………………………..……Page 2

Supplementary Table S2B. Summary of studies assessing cardiometabolic outcomes – At-Risk Populations………………………………………………………………..Pages 3-4

Supplementary Table S2C. Summary of studies assessing cardiometabolic outcomes – Existing Conditions…………………………………………………………….…...…Page 5

Supplementary Table S2D. Summary of studies assessing inflammatory or oxidative outcomes ………………………………………………………..…………..…….….Pages 6-7

Supplementary Table S2E. Summary of studies assessing neurological outcomes ………………………..…………………………………………………….………..…….Pages 8-9

Supplementary Table S2F. Summary of studies assessing gastrointestinal or digestive health outcomes …………………………………………………………….……...…Page 10

Supplementary Table S2G. Summary of studies assessing cancer outcomes ………………………………………………………………………………………………...….Page 11

Supplementary Table S2H. Summary of studies assessing epigenetic, musculoskeletal, or respiratory outcomes …………………………….....……………..…………...….Page 11

Table S2A. Summary of studies assessing cardiometabolic outcomes – Healthy Populations

| **Reference** | **Location** | **Study Design** | **Population** | **Sample size** | **Intervention** | **Duration** |
| --- | --- | --- | --- | --- | --- | --- |
| Istas et al. 2018 | Germany | RCT – Crossover, Multi-arm | Healthy young non-obese males | Total: 10 | Two test groups: 200 g OR 400 g of frozen red raspberry drink; or placebo | Total: 17 days Acute Periods: 3 x 24 hours Washout: 1 week each |
| Istas et al. 2019 | UK | RCT – Three-arm | Healthy males | Total: 86; Extract: 23; Whole fruit: 23; Control: 20 | Two test groups: 500 mg capsules containing either aronia extract OR aronia whole fruit; or placebo | 12 weeks |
| Li et al. 2023 | UK | Cross-sectional | Healthy older female twins (sub-study of TwinsUK Cohort) | Total: 100 Twin pairs Monozygotic: 45 pairs Dizygotic: 55 pairs | N/A | N/A |
| Wood et al. 2023 | UK | RCT - Parallel | Healthy older adults | Total: 61; Wild blueberry (WBB): 32; Placebo: 29 | Freeze-dried WBB at 26 g/day; or placebo | Intervention: 12 weeks Follow-up: 4 weeks |
| Mostafa et al. 2023 | Spain | Cohort study - Prospective | Subsample from DCH-NG MAX study | Baseline: 624; 6 months: 380; 12 months: 349 | N/A | Follow-up of up to 12 months |
| Cortés-Martín et al. 2018a | Spain | Single-arm - Acute | Healthy adolescents and female adults | Total: 68; Adolescents: 40; Adults: 28 | Mixed nuts at 15 g unpeeled walnuts, 7.5 g almonds, 7.5 g hazelnuts per day | 3 days |
| Cortés-Martín et al. 2019 | Spain | Single-arm - Acute | Post-partum, breastfeeding mothers | Total: 40 | Peeled walnuts at 30 g/day | 3 days |
| Jamieson et al. 2024 | USA | RCT - Parallel | Healthy adults | Total: 27; Xanthohumol: 14; Placebo: 13 | Xanthohumol at 24 mg/day; or placebo | 8 weeks |
| Selma et al. 2016 | Spain | Single-arm – Acute (Two Studies) | Healthy adults | Combined: 69; Study 1: 20; Study 2: 49 | Study 1: walnuts at 30 g/day  Study 2: pomegranate extract at 450 mg/day | 3 days each |
| Pallister et al. 2017 | UK | Retrospective | Healthy adult twins | Total: 2218 | N/A | Food intake data collected between 1995 and 2001, in 2007 and 2014 to 2015 |
| Sun et al. 2015 | USA | Case-control | Female registered nurses who did not have T2DM at baseline (NHS I and II) | Total: 2222 NHS I - Cases T2D: 456; Controls: 456 NHS II - Cases T2D: 655; Controls: 655 | N/A | Early follow-up – median 4.6 years  Later follow-up –years not defined |
| NCT03713164 | USA | RCT - Crossover | Healthy adult males with low fiber/polyphenol consumption | Total: 19 | Two test groups: pomegranate juice at 8 oz OR 500 mg pure ellagic acid | Single dose (follow-up over 24 hours) |
| Mills et al. 2017 | UK, Switzerland | RCT – Multi-arm Crossover (Two Studies) | Healthy adult males | Efficacy Study: 15 Proof of Concept : 19 | -Efficacy (Two test groups):  high polyphenol coffee OR low -polyphenol coffee -Proof of Concept (Two test groups): 5-caffeoylquinic acid at 450 mg OR pure 5-caffeoylquinic acid at 900 mg + chlorogenic acids | Proof of concept periods: Single dose with 4 hour follow-up x 4 periods  Efficacy periods: Single dose with 5 hour follow-up x 3 periods  -Washout: 1 week each |
| Rodriguez-Mateos et al. 2019 | Germany | Multiple Trials:  Study 1 and 2: RCT - Crossover Study 3: Single-arm Study 4: RCT - Parallel | Healthy adult males | Total: 55 Study 1: 5 Study 2: 10 Study 3: 5 Study 4: 40 | -Study 1 (two test groups):  anthocyanins at 160 mg, OR a freeze-dried wild blueberry drink at 11 g; or control drinks -Study 2: Anthocyanin capsules at one of 5 difference doses (80, 160, 240, 320, OR 480 mg ACN); or placebo  -Study 3: 11 g wild blueberry powder, equivalent to 100 g fresh wild blueberries, and containing 150 mg of ACNs  -Study 4: 22g WBB per day (11 g wild blueberry powder, bi-daily); or placebo | Study 1: 1-day single dose x 5 periods x 1-week washouts Study 2: 1-day single dose x 6 periods x 1-week washouts Study 3: 28 days Study 4: 28 days |

Table S2B. Summary of studies assessing cardiometabolic outcomes –At-risk Populations

| **Reference** | **Geographical Location** | **Study Design** | **Population** | **Sample size** | **Intervention (if applicable)** | **Duration** |
| --- | --- | --- | --- | --- | --- | --- |
| González-Sarrías et al. 2017 | Spain | RCT – Multi-Arm Crossover | Healthy overweight-obese adults | Total: 49 | Pomegranate extract (Two test groups):  Dose 1 = 450 mg/day, OR Dose 2 = 1800 mg/day;  Or placebo | Total: 24 weeks Intervention periods (2 periods per dose): 4 x 3 weeks each Washouts: 4 x 3 weeks each |
| Grohmann et al. 2023 | UK | RCT - Crossover | Male and post-menopausal female participants at risk of developing T2DM, or those who were diagnosed with pre-diabetes | Total: 14 | Bilberry extract at 250 mg/day + grape seed extract at 300 mg/day; or placebo | Total: 24 weeks Intervention periods: 2 x 12 weeks each |
| Cortés-Martín et al. 2024 | Spain | Secondary analysis of crossover trial | Adults with overweight/obesity | Total: 49 | Pomegranate extract (Two test groups):  Dose 1 = 450 mg/day, OR Dose 2 = 1800 mg/day; or placebo | Total: 24 weeks Intervention periods (2 periods per dose): 4 x 3 weeks each Washouts: 4 x 3 weeks each |
| Lanuza et al. 2023 | Denmark | Cohort Study - Longitudinal | Community-dwelling Danish adults with a range of cardiometabolic health, including healthy individuals, those at elevated risk, and a subgroup (~10%) meeting clinical criteria for metabolic syndrome | Baseline: 676 6 months: 380 12 months: 348 | N/A | Follow-up of up to 12 months |
| Le Sayec et al. 2022 | UK | RCT – Parallel with acute and chronic phases | Adults with prehypertension | Total: 102 Acute Consumption - Aronia berry: 51; Placebo: 51  Chronic Consumption - Aronia berry: 45; Placebo: 48 | Aronia berry extract at 500 mg/day; or placebo | Acute: 2 hours  Chronic: 12 weeks |
| Woolf et al. 2023 | USA | RCT - Parallel | Estrogen-deficient postmenopausal women, with elevated blood pressure or stage-1 hypertension | Total: 43  Blueberry: 22 Placebo: 21 | Freeze-dried highbush blueberry powder at 22 g/day; or placebo | 12 weeks |
| Zheng et al. 2013 | USA | Cohort Study - Prospective | Black adults without hypertension at baseline from the Atherosclerosis Risk in Communities (ARIC); considered in at-risk because follows hypertension risk over time | Total: 896 Non-incident hypertension: 552 Incident hypertension: 344 | N/A | Follow-up of up to 10 years |
| Coelho et al. 2021 | USA | RCT – Acute Crossover (Two Studies) | Adults with overweight/obesity | Total: 34 | Trial 1: Concord grape juice at 355 ml; or placebo  Trial 2: Concord grape juice at 355 ml + 28 g cream of wheat; or placebo | For each trial -  Intervention Periods: 3 x 1 day each (nested within 4-day sessions) Washout: 2 x 6 days each (in between each 4-day session) |
| Flynn 2021 | UK | RCT – Acute Crossover | Older adults with overweight/obesity | Total: 8 (22 Completed at least 1 arm) | Single dose of freeze-dried blueberries at 36 g in 500 ml semi-skimmed milk + test meal; or placebo | Single dose (follow-up over 48 hrs) |
| Marhuenda-Muñoz et al. 2022 | Spain | Case-control | Adults at high CVD risk, but without CVD or diabetes at baseline | Total: 172 Cases: 46 Controls: 126 | N/A | Follow-up of up to one year |
| Zhang et al. 2020 | USA | Single-arm - Acute | Non-smoking adults with pre-diabetes and insulin resistance | Main analysis:  prediabetes and insulin resistance (PreDM-IR): n = 26  healthy Reference group: n = 10  subgroup analysis:  PreDM Lean: 7  Reference-Lean: 8 | Single dose of a red-raspberry drink with equivalent of 250 g fresh raspberry | Single dose (follow-up over 24 hours) |
| Zelicha et al. 2022 | Israel | RCT | Adults with abdominal obesity or dyslipidemia | Total: 286 Healthy Dietary Guidelines (HDG): 98 Mediterranean Diet (MED): 96 Green-MED: 92 | HDG Group: Health-promoting guidelines diet (control)  MED Group: Mediterranean diet +  Walnuts at 28 g/day  Green-MED Group: Mediterranean diet +  Walnuts at 28 g/day +  Green tea at 3-4 cups/day + Mankai at 500 mL/day | 18 months |
| NCT06347094 | Italy | RCT | Adults with cardiometabolic risk factors | Total: 330 | Personalized dietary plan with increased polyphenols; or general dietary guidelines (control) | 24 weeks |
| Huang et al. 2021 | USA | RCT - Crossover | Overweight or obese adults with moderate hypercholesterolemia | Total: 34 | 50 g freeze-dried whole-fruit strawberry powder consumed as 2 beverages/day; or placebo | Total: 12 weeks Intervention periods: 2 x 4 weeks each Washout: 4 weeks |
| Laveriano-Santos et al. 2022 | Spain | Cross-sectional | Adolescents, majority metabolically healthy; crude MetS prevalence 3% | Total: 560 | N/A | N/A |

Table S2C. Summary of studies assessing cardiometabolic outcomes –Existing Conditions

| **Reference** | **Geographical Location** | **Study Design** | **Population** | **Sample size** | **Intervention** | **Control** | **Duration** |
| --- | --- | --- | --- | --- | --- | --- | --- |
| Cortés-Martín et al. 2021 | Spain | RCT - Crossover | Poly-medicated adults with MetS | Total: 50 | pomegranate extract nutraceutical at 900 mg/day; or placebo | Microcrystalline cellulose | Intervention Periods: 2 x 1 month each Washout: 1 month |
| Curtis et al. 2022 | UK | RCT - Acute | Overweight or obese middle-aged to older adults with MetS, and without self-reported cognitive dysfunction | Total: 45  Freeze-dried blueberry powder (BBP): 23  Placebo: 22 | 26 g freeze-dried blueberry powder + challenge meal (500 g milkshake-like emulsion with Protifar) mixture shake; or placebo | Isocaloric and carbohydrate matched purple powder + challenge meal mixture shake | 24 hours |
| Domínguez-López et al. 2023 | Spain | Cross-sectional | Elderly Mediterranean population with at least 3 cardiovascular risk factors: current smoking, HTN, dyslipidemia, overweight/obesity, family history of premature CVD. | Total: 200 | N/A | N/A | N/A |
| Khan et al. 2012 | Spain | RCT - Crossover | Adults with diabetes mellitus or at high risk for cardiovascular disease | Total: 42 | Cocoa powder at 40 g/day with 500 ml skimmed milk; or skim millk (control) | Skimmed milk at 500 ml/day | Intervention Periods: 2 x 4 weeks each |
| Rienks et al. 2017 | Denmark | A Systematic Review and Meta-Analysis of Observational Studies | Adults with acute coronary syndrome | Total: 1179 Acute Coronary Syndrome: 393 Controls: 786 | N/A | N/A | N/A |
| Mora-Cubillos et al. 2015 | Spain | RCT - Parallel | Adults with at least three MetS risk factors | Total: 47 Nuts: 24 Control: 23 | Raw mixed nuts with skin at 30 g/day + low-fat diet following dietary recommendations according to the AHA dietary guidelines; or avoid nuts (control) | Avoid consumption of nuts (+ follow same low-fat diet as intervention) | 12 weeks |
| Meroño et al. 2022 | Italy | RCT - Crossover | Older adults (≥60 yr) living in a residential care setting, with increased intestinal permeability* | Total: 51 | 3 small portions/day polyphenol-rich snacks; or low polyphenol control snacks | 3 small portions/day control snacks (low in polyphenols) | Total: 3 months Test periods: 2 x 8 weeks Washout: 8 weeks |
| Bullón-Vela et al. 2020 | Spain | Cross-sectional | Individuals with overweight/obesity and at least three MetS components | Total: 266 | N/A | N/A | N/A |
| Cortés-Martín et al. 2018b | Spain | Single-Arm (Multiple studies - including current and secondary analysis) | Current study included healthy children, adolescents, and adults; Past studies included adults with MetS, prostate cancer, and colorectal cancer | Current study - Healthy Children (5-12y): 202 Healthy Adolescents (13-18y): 221 Healthy Adults (19-72y): 221  Secondary analysis -  Healthy Adults: 109 Metabolic Syndrome Adults: 23 Prostate Cancer Adults: 28 Colorectal Cancer Adults: 35 | Children/Adolescents: peeled walnuts at 25 g/day, OR pomegranate juice at 250 mL  Adults: Pomegranate extract at 450 mg/day OR peeled walnuts 30 g/day | N/A | 3 days |

Table S2D. Summary of studies assessing immunological or oxidative outcomes

| **Reference** | **Geographical Location** | **Study Design** | **Population** | **Sample size** | **Intervention** | **Control** | **Duration** |
| --- | --- | --- | --- | --- | --- | --- | --- |
| Cortés-Martín et al. 2021 | Spain | RCT - Crossover | Adults with metabolic Syndrome (MetS) under polypharmacological treatment | Total: 50 Arm 1: 23 Arm 2: 27 | Pomegranate extract nutraceutical 900 mg/day | 900 mg/day microcrystalline cellulose | Phase 1: 1 month Washout: 1 month Phase 2: 1 month |
| Barnes et al. 2019 | USA | RCT – Three arm | Healthy lean and obese adults without digestive disorders | Total: 32 Lean (BMI<25), Control: 11 Lean (BMI<25), Mango: 12 Obese (BMI>30), Mango: 9 | Mango pulp 400 g/day | Lean control - 400g mango only on Days 0 and 42 | 6 weeks |
| Angelino et al. 2020 | UK, Ireland, Italy | Protocol for Cohort Study | Community-dwelling older adults without diagnosed dementia | Total: 6186  Original TUDA: 5186  TUDA5+: 1000 | N/A | N/A | Follow-up of up to 7 years |
| Cerdá et al. 2006 | Spain | RCT – Parallel | Adults with stable COPD | Total: 30 Pomegranate juice: 15 Placebo: 15 | Pomegranate juice 400mL/day | 400 mL matched placebo drink | 5 weeks |
| González‐Sarrías et al. 2018 | Spain | RCT - Crossover | Overweight-obese adults | Total: 49 Overweight: 29 Obese: 20 | Pomegranate extract at:  Dose 1 = 450 mg/day, OR Dose 2 = 1800 mg/day | maltodextrin | Total: 24 weeks Intervention periods (2 periods per dose): 4 x 3 weeks each Washouts: 4 x 3 weeks each |
| Gutiérrez-Díaz et al. 2018 | Spain | Cross-sectional | Healthy, older adults | Total: 71 | N/A | N/A | N/A |
| Guo et al. 2023 | China | Cross-sectional | Adults with pemphigus | Total: 69 Pemphigus: 43 Controls: 26 | N/A | N/A | N/A |
| Langley et al. 2022 | USA | Protocol for RCT | Adults with clinically active Crohn’s disease | Total: 32 Intervention: 16 Control: 16 | Xanthohumol at 24 mg/day | Encapsulated rice protein placebo | 8 weeks |
| Kim et al. 2017 | USA | Single-arm | Lean and obese human adults | Total: 21  Lean: 12  Obese: 9 | Mango 400g/day | N/A | 6 weeks |
| Vitaglione et al. 2015 | Italy | RCT – Parallel | Healthy overweight or obese adults with low intake of fruit and vegetables and sedentary lifestyle | Total: 68 Whole grain (WG): 36 Control (CTR): 32 | Whole grain biscuits at 70 g/day | Low polyphenol replacement: 1 package (33 g) of crackers and 3 slices of toasted bread (~27 g) per day | 8 weeks |
| Meroño et al. 2022 | Italy | RCT - Crossover | Older adults (≥60 yr) living in a residential care setting, , with increased intestinal permeability | Total: 51 | 3 small portions/day polyphenol-rich snacks | 3 small portions/day control snacks (low in polyphenols) | Total: 3 months Test periods: 2 x 8 weeks Washout: 8 weeks |
| Harms et al. 2020 | Europe (France, Italy, Spain, the UK, The Netherlands, Greece, Germany, Sweden, Denmark and Norway) | Cross-sectional | Adults >35 years old from European countries, free of major chronic diseases | Total: 315 | N/A | N/A | N/A |
| Dorjgochoo et al., 2012 | China | Cross-Sectional | Healthy middle-aged and elderly Chinese women | Total: 845 | N/A | N/A | N/A |
| Bullón-Vela et al., 2023 | Spain | Cross-sectional | Overweight/obese adults diagnosed with MetS | Total: 267 | N/A | N/A | N/A |
| Lanuza et al. 2023 | Denmark | Longitudinal Cohort | Adults from Copenhagen and their biological children, spouses, and grandchildren | Baseline: 676 6 months: 380  12 months: 348 | N/A | N/A | N/A |

Table S2E. Summary of studies assessing neurological outcomes

| **Reference** | **Geographical location** | **Study Design** | **Population** | **Sample Size** | **Intervention** | **Control** | **Duration** |
| --- | --- | --- | --- | --- | --- | --- | --- |
| Angelino et al., 2020 | UK, Ireland, Italy | Protocol for Cohort Study | Community-dwelling older adults without diagnosed dementia | Total: 6186 Original TUDA: 5186 TUDA5+: 1000 | N/A | N/A | Follow-up of up to 7 years |
| Bensalem et al., 2018 | Canada and France | RCT - Parallel | Healthy older adults with mild memory decline | Total: 215 PEGB Group: 101 Placebo Group: 105 | Combined grape and wild blueberry extracts at 600 mg/day | Maltodextrin | 6 months |
| González-Domínguez et al., 2020 | France | Cohort Study - Prospective | Cognitive decline in older adults | Bordeaux sample set (training) Cases: 209 Controls: 209  Dijon sample set (validation) Cases: 212 Controls: 212 | N/A | N/A | Follow-up of up to 12 years |
| Curtis et al., 2024 | UK | RCT – Multi-arm with acute and chronic phases | Overweight or obese middle-aged to older adults with metabolic syndrome, but without self-reported cognitive dysfunction | Chronic Study -  Total: 115 Blueberry Powder (BBP) Dose 1 (26g): 37 BBP Dose 2 (13g): 39 Placebo: 39  Acute (Postprandial) Study -  Total: 33 BBP Dose 1 (26g): 16 Placebo: 17 | Chronic study (two test groups):  BBP at 26 g/day OR  BBP at 13 g/day  Acute (Postprandial) sub-study: BBP at 26 g/day + challenge meal at 500 g/day | Isocaloric and carbohydrate-matched powder (26 g/day) | Chronic: 6 months Acute (Postprandial) sub-study: 24 hours |
| Domínguez-López et al., 2024 | Spain | Cross-sectional | Overweight or obese middle-aged to older adults with metabolic syndrome | Total: 400 | N/A | N/A | N/A |
| Flanagan, 2021 | UK | RCT - Parallel | Middle-aged to older adults with no subjective or objective memory complaints (cognitive impairment) | Total: 60  Cranberry: 29 Placebo: 31 | Freeze-dried cranberry powder at 9 g/day | Isocaloric, macronutrient, taste and color-matched powder | 12 weeks |
| Parilli-Moser et al., 2023 | Spain | RCT – Three-arm | Healthy adults | Total: 63  Skin-roasted peanuts (SRP): 21  Peanut butter (PB): 23  Control butter (CB): 19 | Two test groups - Skin-roasted peanuts at 25 g/day OR  peanut butter at two tablespoons (~32 g)/day | Control butter based on peanut oil and free of fiber and polyphenols | 6 months |
| Rabassa et al., 2020 | Italy | Cohort Study - Prospective | Dementia-free older adults | Total: 119 Non-consumers: 72 Regular consumers: 47 | N/A | N/A | Follow-up of up to 3 years |
| Romo-Vaquero et al., 2022 | Spain | Single-Arm – Acute (with Parallel Condition Comparison) | Patients with Parkinson's disease | Total: 169 Parkinson's disease: 52 Healthy controls: 117 | Walnuts at 30 g/day | N/A | 3 days |
| Volpp et al., 2020 | Slovakia | RCT - Parallel | Female adults with a medical indication for vaginal hysterectomy | Total: 66 Oak wood extract  (Robuvit®): 33 Placebo: 33 | Proprietary oak wood extract (Robuvit®) at 300 mg/day | Appearance and taste-matched placebo capsule, but without oak-wood extract | 8-weeks post-surgery |
| Wood et al., 2023 | UK | RCT - Parallel | Healthy older adults | Total: 61 Wild blueberry (WBB): 32 Placebo: 29 | Freeze-dried WBB powder at 26 g/day | Appearance, taste, and macronutrient, fiber and vitamin C–matched placebo powder | Intervention: 12 weeks  Follow-up: 4 weeks |

Table S2F. Summary of studies assessing gastrointestinal or digestive health outcomes

| **Reference** | **Geographical Location** | **Study Design** | **Population** | **Sample size** | **Intervention** | **Control** | **Duration** |
| --- | --- | --- | --- | --- | --- | --- | --- |
| Hidalgo-Liberona et al., 2020 | Italy | RCT - Crossover | Older adults (≥60 yr) living in a residential care setting, with increased intestinal permeability | Total: 51  Lower serum zonulin at baseline (LSZ): 26 Higher serum zonulin at baseline (HSZ): 25 | 3 small portions/day polyphenol-rich snacks | 3 small portions/day control snacks (low in polyphenols) | Total: 3 months Test periods: 2 x 8 weeks Washout: 8 weeks |
| Peron et al., 2021 | Italy | RCT - Crossover | Older adults (≥60 yr) living in a residential care setting, with increased intestinal permeability | Total: 51 | 3 small portions/day polyphenol-rich snacks | 3 small portions/day control snacks (low in polyphenols) | Total: 3 months Test periods: 2 x 8 weeks Washout: 8 weeks |
| Langley et al., 2022 | USA | Protocol for RCT | Adults with clinically active Crohn’s disease | Total: 32 Intervention: 16 Control: 16 | Xanthohumol at 24mg/day | Encapsulated rice protein placebo | 8 weeks |
| Karl et al. 2022 | USA | Secondary analysis of RCT | Healthy adults, including both military and civilians | Total: 54 Control diet (CON): 27 Meal, Ready-to-Eat (MRE): 27 | 2-3 MRE meals/day | Habitual diet | 3 phases:  Baseline Period: 9 days Intervention Period: 21 days Washout Period: 10 days |
| Meroño et al., 2022 | Italy | RCT - Crossover | Older adults (≥60 yr) living in a residential care setting | Total: 51 | 3 small portions/day polyphenol-rich snacks | 3 small portions/day control snacks (low in polyphenols) | Total: 3 months Test periods: 2 x 8 weeks Washout: 8 weeks |
| Nishioka et al., 2021 | Brazil | RCT – Acute Crossover | Women, obese or non-obese | Total: 27 Non-obese: 17 Obese: 10 | Pera orange juice OR blood orange juice at 600 mL | N/A | Single dose per period;  Washout: 1 week |
| Coelho et al. 2021 | USA | RCT – Acute Crossover (Two Studies) | Adults with overweight/obesity | Total: 34 | Trial 1: Concord grape juice at 355 ml  Trial 2: Concord grape juice at 355 ml + 28 g cream of wheat | Placebo | For each trial -  Intervention Periods: 3 x 1 day each (nested within 4-day sessions) Washout: 2 x 6 days each (in between each 4-day session) |

Table S2G. Summary of studies assessing cancer outcomes

| **Reference** | **Geographical Location** | **Study Design** | **Population** | **Sample size** | **Intervention** | **Control** | **Duration of Intervention** |
| --- | --- | --- | --- | --- | --- | --- | --- |
| González-Sarrías et al., 2010 | Spain | RCT - Parallel | Adult males with prostate cancer or benign prostatic hyperplasia | Total: 63 Controls: 30 Walnuts: 14 Pomegranate juice: 19 | Walnuts at 35 g/day OR pomegranate juice at 200 mL/day | No PJ or walnut intake | 3 days |
| Luo et al., 2010 | China | Nested case-control | Adult women in China without breast cancer diagnosis at baseline | Total: 1053 Cases: 352 Controls: 701 | N/A | N/A | N/A |
| Sun et al., 2002 | China | Nested case-control | Middle-aged Chinese males with and without gastric and esophageal cancers | Total: 1004  Gastric cancer: 190  Esophageal cancer: 42  Controls: 772 | N/A | N/A | N/A |
| Pan et al., 2015 | USA | Single-arm | Patients with diagnosed colorectal cancer scheduled for surgical resection | Total: 28 | Freeze-dried black raspberries at 60 g/day | N/A | From 1-9 weeks Mean: 4 weeks |

Table S2H. Summary of study assessing epigenetic, musculoskeletal, or respiratory outcomes

| **Reference** | | **Geographical Location** | **Study Design** | | **Population** | | **Sample Size (n)** | | **Intervention** | | **Control** | | **Duration of Intervention** | | **Outcomes** | |
| --- | --- | --- | --- | --- | --- | --- | --- | --- | --- | --- | --- | --- | --- | --- | --- | --- |
| Meir et al., 2023 | | Israel | RCT - Three-arm | | Adults with abdominal obesity and/or dyslipidemia | | Total: 294   HDG: 88  MED: 81  Green-MED: 87 | | HDG Group: Health-promoting guidelines diet  MED Group: Mediterranean diet +  Walnuts at 28 g/day  Green-MED Group: Mediterranean diet +  Walnuts at 28 g/day +  Green tea at 3-4 cups/day + Mankai at 500 mL/day | | N/A | | 18 months | | Epigenetic - Li mAge | |
| Greenbaum et al., 2022 | | China | Cross-sectional | | Healthy post-menopausal and peri-menopausal females | | Total: 499 Postmenopausal: 419 Perimenopausal: 80 | | N/A | | N/A | | N/A | | Musculoskeletal - Bone mineral density (BMD) | |
| Cerdá et al. 2005 | Spain | | | RCT – Parallel | | Adults with stable COPD | | Total: 30 Pomegranate juice: 15 Placebo: 15 | | Pomegranate juice 400mL/day | | 400 mL matched placebo drink | | 5 weeks | | Respiratory |
