# Supplementary material for: Clinical evidence for microbial-derived polyphenol metabolites in health and disease: a scoping review
Source: Front Nutr. 2026 Jun 17;13:1859472. doi: 10.3389/fnut.2026.1859472 (PMC13319019; doi:10.3389/fnut.2026.1859472)
Supplement: Supplementary file 4 [file Table_3.DOCX]

**Supplementary Tables 3A-E: Microbiome Characterization and MPM–Microbiota Relationships**

The following tables summarize studies included in this review that statistically evaluated relationships between microbial-derived polyphenol metabolites (MPMs) and/or metabotypes and gut microbiota features. For each outcome domain, tables report study design, microbiome characterization methods, microbial outcomes assessed (taxa, diversity, and/or function), and reported associations between MPMs or metabotypes and gut microbiota characteristics. If an outcome domain is not represented, no studies within that domain assessed relationships between MPMs and gut microbiota.

**Supplementary Table S3A.** Microbiome characterization and MPM–microbiota relationships in studies reporting cardiometabolic outcomes ……………………………………….……. Page 2

**Supplementary Table S3B.** Microbiome characterization and MPM–microbiota relationships in studies reporting immunological and oxidative outcomes ………………………………. Page 3

**Supplementary Table S3C.** Microbiome characterization and MPM–microbiota relationships in studies reporting neurological outcomes …………………………………………………. Page 4

**Supplementary Table S3D.** Microbiome characterization and MPM–microbiota relationships in studies reporting gastrointestinal and digestive health outcomes …………………..……. Page 5

**Supplementary Table S3F.** Microbiome characterization and MPM–microbiota relationships in studies reporting musculoskeletal outcomes ……………………………………………. Page 5

**Supplemental Table S3A.** Summary of microbiome characterization approaches used in studies reporting cardiometabolic outcomes.

| **Study Design** | **Sequencing Methods** | **Microbial Outcomes Measured (Taxa, Diversity, and/or Function)** | **Were MPMs associated with microbial outcomes?** |
| --- | --- | --- | --- |
| **Observational (n=2)** |  |  |  |
| **Cross-sectional (n=2)** | **16s rRNA: 2** |  |  |
| Li et al., 2023 | 16s rRNA | Taxa, α-diversity (Shannon index), β-diversity (PCA) | Phenolic acids and simple phenols ↑&↓ Taxa |
| Pallister et al., 2017 | 16s rRNA | Taxa only | Hippuric acid derivatives ↑&↓ Taxa |
| **Interventional (n= 10)** |  |  |  |
| **Single Arm - Acute (<14 days) (n=4)** | **16s rRNA: 2; MetaG: 1; qPCR: 1** |  |  |
| Cortés-Martín et al., 2019 | 16s rRNA | Taxa, α-diversity (Chao1, Shannon index) | Taxa abundance and taxa re-stabilization postpartum differs by urolithin metabotypes |
| Selma et al., 2016 | qPCR | Taxa only | Taxa abundance differs by urolithin metabotypes |
| Zhang et al., 2020 | MetaG | Taxa, α-diversity (Shannon index), β-diversity (metric mot specified) | Phenolic Acids, simple phenols, and hippuric acid derivatives ↑&↓ Taxa |
| **RCT-Parallel >14 days (n=4)** | **16s rRNA: 3; MetaG: 1** |  |  |
| Istas et al., 2019 | 16s rRNA | Taxa, α-diversity (metric not specified), β Diversity (metric not specified) | Phenolic Acids, simple phenols, and hippuric acid derivatives ↑&↓ Taxa |
| Jamieson et al., 2024 | 16s rRNA | Taxa, α-diversity (Shannon and Simpson indices, Richness) & β diversity (Bray Curtis and Jaccard) | Enterotypes associated with xanthoumol MPMs |
| Le Sayec et al., 2022 | MetaG | Taxa, α-diversity (Shannon and Simpson indices), β-diversity (Bray-Curtis) | Phenolic Acids, simple phenols, and hippuric acid derivatives ↑&↓ Taxa |
| Wood et al., 2023 | 16s rRNA | Taxa, α-diversity (metric not specified), β-Diversity (Bray–Curtis) | Association between MPMs and taxa not assessed |
| **RCT - Crossover, >14 days (n=3)** | **16s rRNA: 2; qPCR: 1** |  |  |
| Cortés-Martín et al., 2021 | 16s rRNA | Taxa only | Taxa abundance differed by urolithin metabotype |
| González-Sarrías et al., 2017 | qPCR | Taxa, α-diversity (Shannon Index and Chao1), β-diversity (Bray-Curtis) | Taxa abundance differed by urolithin metabotype |
| Meroño et al., 2022 | 16s rRNA | Taxa, α-diversity (Shannon, Pielou’s evenness, and Faith’s phylogenetic diversity indices), β-diversity (UniFrac weighted and unweighted) | Phenylpropionic acids ↑ Taxa |
| **Secondary Analyses (n=1)** | **16s rRNA:1** |  |  |
| Cortés-Martín et al., 2024 | 16s rRNA: 1 | Taxa only | Urolithin-A ↓ Taxa |
| **Table Totals: 14 studies** | **Seq. Method Totals** | **Outcomes Totals** |  |
| **Observational: 2 (14.29%); Interventional: 12 (85.71%)** | **16s rRNA: 10; MetaG:2; qPCR: 2** | **Taxa: 11; α-Diversity: 9; β-diversity: 7** |  |

**Supplemental Table S3A.** Summary of microbiome characterization approaches used in studies reporting cardiometabolic outcomes. Studies are grouped by study design and categorized according to microbiome sequencing or detection methods, microbial outcomes assessed (taxonomic composition, α-diversity, β-diversity), and an overview of relationships between MPMs or metabotypes and gut microbiota features. Counts provided in section headers represent the number of studies within each study design category. Table totals summarize the frequency of microbiome characterization methods and microbial outcomes assessed across included studies. 16S rRNA = 16S ribosomal RNA gene sequencing; MetaG = metagenomic shotgun sequencing; qPCR = quantitative polymerase chain reaction. Upward arrows (↑) indicate positive associations and downward arrows (↓) indicate negative associations between MPMs, metabotypes, and reported microbial outcomes. Studies reporting both positive and negative associations are denoted by ↑&↓.

**Supplemental Table S3B.** Summary of microbiome characterization approaches used in studies reporting inflammatory and oxidative stress outcomes.

| **Study Design** | **Sequencing Methods** | **Microbial Outcomes Measured (Taxa, Diversity, and/or Function)** | **Were MPMs associated with microbial outcomes?** |
| --- | --- | --- | --- |
| **Observational (n=1)** |  |  |  |
| **Cross-sectional (n=1)** | **16s rRNA: 1** |  |  |
| Gutiérrez-Díaz et al., 2018 | 16s rRNA | Taxa only | Phenolic acids ↓ Taxa |
| **Interventional (n= 5)** |  |  |  |
| **RCT - Crossover, >14 days (n=4)** | **16s rRNA: 4** |  |  |
| Cortés-Martín et al., 2021 | 16s rRNA | Taxa only | Taxa abundance differed by urolithin metabotype |
| Vitaglione et al., 2015 | 16s rRNA | Taxa, β-diversity (UniFrac weighted and unweighted) | Hippuric acid derivatives ↑&↓ Taxa |
| Meroño et al., 2022 | 16s rRNA | Taxa, α-diversity (Shannon, Pielou’s evenness, and Faith’s phylogenetic diversity indices), β-diversity (UniFrac weighted and unweighted) | Phenylpropionic acids ↑ Taxa |
| **RCT - Multi-arm, >14 days (n=1)** | **16s rRNA: 1** |  |  |
| Barnes et al., 2019 | 16s rRNA | Taxa only | Gallotannin metabolites ↑ Taxa |
| **Table Totals: 6 studies** | **Seq. Method Totals** | **Outcomes Totals** |  |
| **Observational: 1; Interventional: 5** | **16s rRNA: 6** | **Taxa only: 6; α-Diversity: 2; β-diversity: 2** |  |

**Supplemental Table S3B.** Summary of microbiome characterization approaches used in studies reporting inflammatory and oxidative stress outcomes. Studies are grouped by study design and categorized according to microbiome sequencing or detection methods, microbial outcomes assessed (taxonomic composition, α-diversity, β-diversity), and an overview of relationships between MPMs or metabotypes and gut microbiota features. Counts provided in section headers represent the number of studies within each study design category. Table totals summarize the frequency of microbiome characterization methods and microbial outcomes assessed across included studies. 16S rRNA = 16S ribosomal RNA gene sequencing; MetaG = metagenomic shotgun sequencing. Upward arrows (↑) indicate positive associations and downward arrows (↓) indicate negative associations between MPMs, metabotypes, and reported microbial outcomes. Studies reporting both positive and negative associations are denoted by ↑&↓.

**Supplemental Table S3C.** Summary of microbiome characterization approaches used in studies reporting neurological outcomes.

| **Study Design** | **Sequencing Methods** | **Microbial Outcomes Measured (Taxa, Diversity, and/or Function)** | **Were MPMs associated with microbial outcomes?** |
| --- | --- | --- | --- |
| **Interventional (n= 3)** |  |  |  |
| **Single Arm - Acute (<14 days) (n=1)** | **16s rRNA: 1** |  |  |
| Romo-Vaquero et al., 2022 | 16s rRNA | Taxa only | Taxa abundance differs by urolithin metabotypes & correlation with disease stage/severity |
| **RCT-Parallel >14 days (n=2)** | **16s rRNA: 1; MetaG: 1** |  |  |
| Flanagan, 2021 | MetaG | Taxa, α diversity (Shannon index), β diversity (Bray-Curtis) | Hippuric acid derivatives ↑ Taxa |
| Wood et al., 2023 | 16s rRNA | Taxa, α-diversity (metric not specified), β-Diversity (Bray–Curtis) | Association between MPMs and taxa not assessed |
| **Table Totals: 3 studies** | **Seq. Methods Totals** | **Outcomes Totals** |  |
| **Interventional: 3** | **16s rRNA: 2; MetaG: 1** | **Taxa: 3; α-Diversity: 2; β-diversity: 2** |  |

**Supplemental Table S3C.** Summary of microbiome characterization approaches used in studies reporting neurological outcomes. Studies are grouped by study design and categorized according to microbiome sequencing or detection methods, microbial outcomes assessed (taxonomic composition, α-diversity, β-diversity), and an overview of relationships between MPMs or metabotypes and gut microbiota features. Counts provided in section headers represent the number of studies within each study design category. Table totals summarize the frequency of microbiome characterization methods and microbial outcomes assessed across included studies. 16S rRNA = 16S ribosomal RNA gene sequencing; MetaG = metagenomic shotgun sequencing. Upward arrows (↑) indicate positive associations and downward arrows (↓) indicate negative associations between MPMs, metabotypes, and reported microbial outcomes. Studies reporting both positive and negative associations are denoted by ↑&↓.

**Supplemental Table S3D.** Summary of microbiome characterization approaches used in studies reporting gastrointestinal and digestive health outcomes.

| **Study Design** | **Sequencing Methods** | **Microbial Outcomes Measured (Taxa, Diversity, and/or Function)** | **Were MPMs associated with microbial outcomes?** |
| --- | --- | --- | --- |
| **Interventional (n= 3)** |  |  |  |
| **RCT - Crossover, Acute (n=1)** | **16s rRNA: 1** |  |  |
| Nishioka et al., 2021 | 16s rRNA | Taxa only | Taxa abundance differed by MPM excretion profile |
| **RCT - Crossover, >14 days (n=2)** | **16s rRNA: 2** |  |  |
| Meroño et al., 2022 | 16s rRNA | Taxa, α-diversity (Shannon, Pielou’s evenness, and Faith’s phylogenetic diversity indices), β-diversity (UniFrac weighted and unweighted) | Phenylpropionic acids ↑ Taxa |
| Peron et al., 2021 | 16s rRNA | Taxa, α-diversity (Shannon, Pielou’s evenness, and Faith’s phylogenetic diversity indices), β-diversity (UniFrac weighted and unweighted) | Hippuric acid derivatives ↑&↓ Taxa |
| **Table Totals: 3 studies** | **Seq. Method Totals** | **Outcomes Totals** |  |
| **Interventional: 3** | **16s rRNA: 3** | **Taxa: 3; α-Diversity: 2; β-diversity: 2** |  |
|  |  |  |  |

**Supplemental Table S3D.** Summary of microbiome characterization approaches used in studies reporting gastrointestinal and digestive health outcomes. Studies are grouped by study design and categorized according to microbiome sequencing or detection methods, microbial outcomes assessed (taxonomic composition, α-diversity, β-diversity), and an overview of relationships between MPMs or metabotypes and gut microbiota features. Counts provided in section headers represent the number of studies within each study design category. Table totals summarize the frequency of microbiome characterization methods and microbial outcomes assessed across included studies. 16S rRNA = 16S ribosomal RNA gene sequencing; MetaG = metagenomic shotgun sequencing. Upward arrows (↑) indicate positive associations and downward arrows (↓) indicate negative associations between MPMs, metabotypes, and reported microbial outcomes. Studies reporting both positive and negative associations are denoted by ↑&↓.

**Supplemental Table S3E.** Summary of microbiome characterization approaches used in studies reporting musculoskeletal outcomes.

| **Study Design** | **Sequencing Methods** | **Microbial Outcomes Measured (Taxa, Diversity, and/or Function)** | **Were MPMs associated with microbial outcomes?** |
| --- | --- | --- | --- |
| **Observational (n=1)** |  |  |  |
| **Cross-sectional (n=1)** | **MetaG: 1** |  |  |
| Greenbaum et al., 2022 | MetaG | Taxa only | 3-Phenylpropanoic acid and Hippuric acid ↓ Taxa |
| **Table Totals: 1 study** | **Seq. Method Totals** | **Outcomes Totals** |  |
| **Observational: 1** | **MetaG: 1** | **Taxa: 1** |  |

**Supplemental Table S3B.** Summary of microbiome characterization approaches used in studies reporting musculoskeletal outcomes. Studies are grouped by study design and categorized according to microbiome sequencing or detection methods, microbial outcomes assessed (taxonomic composition, α-diversity, β-diversity), and an overview of relationships between MPMs or metabotypes and gut microbiota features. Counts provided in section headers represent the number of studies within each study design category. Table totals summarize the frequency of microbiome characterization methods and microbial outcomes assessed across included studies. 16S rRNA = 16S ribosomal RNA gene sequencing; MetaG = metagenomic shotgun sequencing. Upward arrows (↑) indicate positive associations and downward arrows (↓) indicate negative associations between MPMs, metabotypes, and reported microbial outcomes. Studies reporting both positive and negative associations are denoted by ↑&↓.
